# β-l-*Arabino*furano-cyclitol Aziridines Are Covalent Broad-Spectrum Inhibitors and Activity-Based Probes for Retaining β-l-Arabinofuranosidases

**DOI:** 10.1021/acschembio.3c00558

**Published:** 2023-12-05

**Authors:** Valentina Borlandelli, Wendy Offen, Olga Moroz, Alba Nin-Hill, Nicholas McGregor, Lars Binkhorst, Akihiro Ishiwata, Zachary Armstrong, Marta Artola, Carme Rovira, Gideon J. Davies, Herman S. Overkleeft

**Affiliations:** † Bio-organic Synthesis, Leiden Institute of Chemistry (LIC), Leiden University, Gorlaeus Laboratories, Einsteinweg 55, 2333 CC Leiden, The Netherlands; ‡ Department of Chemistry, York Structural Biology Laboratory, University of York, Heslington, York YO10 5DD, United Kingdom; § Departament de Química Inorgànica i Orgànica (Secció de Química Orgànica), Institut de Química Teòrica i Computacional (IQTCUB), Universitat de Barcelona, Martí i Franquès 1, 08028 Barcelona, Spain; ∥ RIKEN Cluster for Pioneering Research, 2-1 Hirosawa, Wako, Saitama 351-0198, Japan

## Abstract

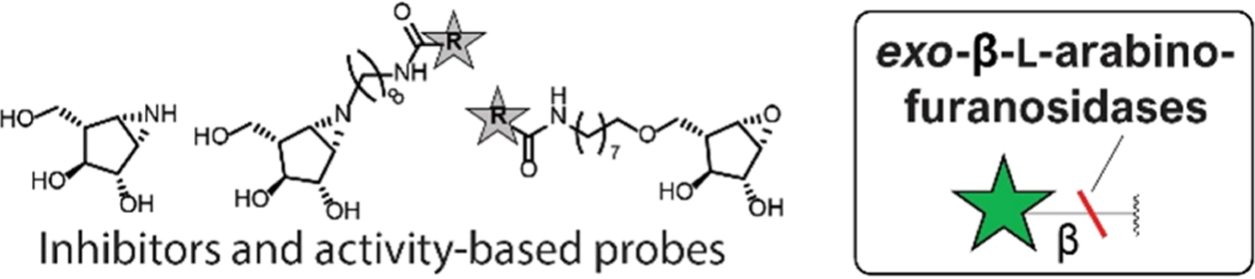

GH127 and GH146 microorganismal retaining β-l-arabinofuranosidases, expressed by human gut microbiomes, feature an atypical catalytic domain and an unusual mechanism of action. We recently reported that both *Bacteroides thetaiotaomicronBt*GH146 and *Bifidobacterium longum* HypBA1 are inhibited by β-l-*arabino*furanosyl cyclophellitol epoxide, supporting the action of a zinc-coordinated cysteine as a catalytic nucleophile, where in most retaining GH families, an aspartate or glutamate is employed. This work presents a panel of β-l-*arabino*furanosyl cyclophellitol epoxides and aziridines as mechanism-based *Bt*GH146/HypBA1 inhibitors and activity-based probes. The β-l-*arabino*furanosyl cyclophellitol aziridines both inhibit and label β-l-arabinofuranosidase efficiently (however with different activities), whereas the epoxide-derived probes favor *Bt*GH146 over HypBA1. These findings are accompanied by X-ray structural analysis of the unmodified β-l-*arabino*furanosyl cyclophellitol aziridine in complex with both isozymes, which were shown to react by nucleophilic opening of the aziridine, at the pseudoanomeric carbon, by the active site cysteine nucleophile to form a stable thioether bond. Altogether, our activity-based probes may serve as chemical tools for the detection and identification of low-abundance β-l-arabinofuranosidases in complex biological samples.

β-l-Arabinofuranosidases hydrolyze β-l-*arabino*furanosyl units from arabino-oligosaccharides (AOS), complex pectic dietary glycans,^1^ and plant-derived glycoproteins such as solanaceous lectins.^2,3^ As is the case for retaining α-l-arabinofuranosidases,^4−8^ known β-l-arabinofuranosidases have been identified in the proteome of prokaryotic micro-organismal species colonizing the human gut^1^ including the gut commensal bacteria *Bacteroides thetaiotaomicron* and *Bifidobacterium longum*. Gut microbiomes express several carbohydrate-active enzymes (CAZymes)^9^ for the utilization of specific plant-sourced polysaccharide substrates, which act as nutrients for bacteria and fungi in the gastrointestinal tract.

β-l-Arabinofuranosidases are encoded in AOS utilization genes by several *Bifidobacterium* species,^10^ whereas some *Bacteroides* gut microbial species express these enzymes specifically for pectin degradation.^1^ Two representative examples of gut microbiome β-l-arabinofuranosidases and the subject of the herein-presented studies are HypBA1 (classified within GH127 and expressed by *B. longum*) and *Bt*GH146 (GH146, expressed by *B. thetaiotaomicron*). We here show that tagged β-l-*arabino*furanosyl cyclophellitol aziridines are versatile reagents for studying HypBA1 and *Bt*GH146 by activity-based protein profiling.

Biochemical and structural studies on HypBA1 established the retaining activity of this GH,^11^ which was found to be capable of hydrolyzing terminal β-l-*arabino*furanosides with retention of the configuration at the anomeric center. Crystallographic evidence for the distance (4.9 Å)^12^ between two carboxylates (E322 and E338) initially supported a classical two-carboxylate mechanism of action. However, the presence of a coordination system made of three cysteines and one glutamate centered around a zinc cation within the catalytic domain of HypBA1^12,14^ suggested alternative mechanisms to be in play. Site-directed mutagenesis^11^ and crystallographic^12,14^ experiments revealed the presence of a cysteine nucleophile, rather than a glutamate or aspartate as in canonical glycosidases.

By making use of the mechanism-based inhibitor, β-l-*arabino*furanosyl cyclophellitol epoxide^16^ (**1**, Figure 1), we recently obtained structural and biochemical evidence for the nature of the nucleophilic residue in HypBA1 and *Bt*GH146, which in both cases turned out to be a cysteine (C417 in HypBA1, C416 in *Bt*GH146) as the nucleophile. The crystal structure of **1** in complex with *Bt*GH146 revealed an unexpected regiochemical reactivity: the catalytic nucleophile C416 appeared to have formed a covalent bond with the pseudoanomeric carbon (that is, C6 in **1**), instead of the expected carbon^17−21^ corresponding to the anomeric center of the parent substrate (C1 in 1), while the HypBA1 catalytic nucleophilic cysteine reacted at the carbon mimicking the anomeric center (C1). This difference in the regiochemical outcome for the enzymatic reaction with **1** by the two enzymes was attributed to the slow inactivation rate of the epoxide scaffold and to structural differences between the active sites of the two enzymes.

**Scheme 1:**
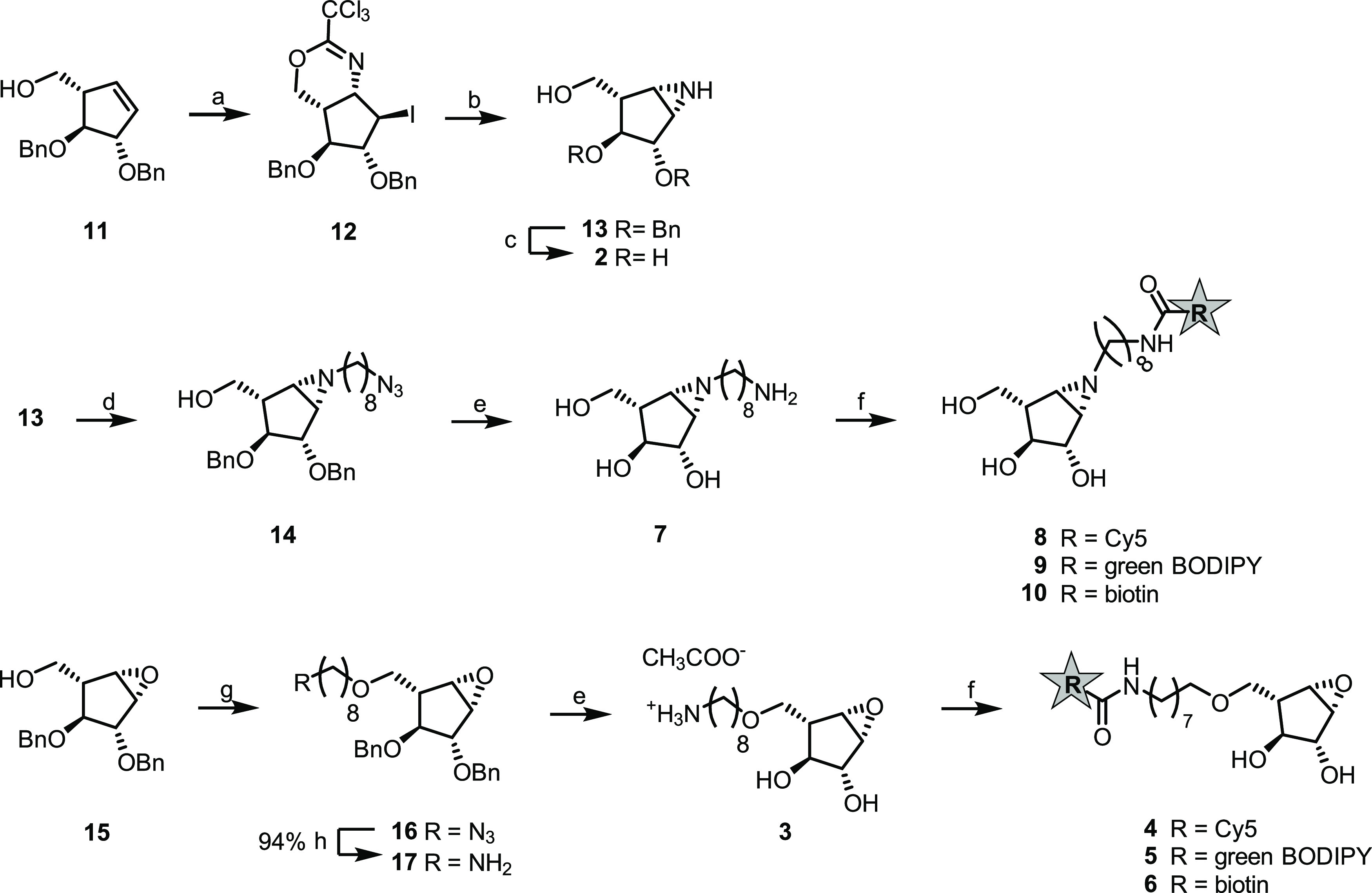
Synthesis of **2**–**10** Reagents and conditions: (a) i. CCl_3_CN, DBU, DCM, 0 °C → rt, 3 h; ii. NIS, CHCl_3_, 0 °C → rt, 17 h, and quant. over two steps. (b) i. HCl in CH_3_OH, DCM/CH_3_OH, and 6 h and ii. Amberlite IRA-67, 1 day; and 63% over 2 steps. (c) Na(s), NH_3_, THF/^*t*^BuOH, −60 °C, 45 min, and 23%; (d) 8-azido-octyl-1-*O*-triflate,^24^ DIPEA, DCM, −15 °C → rt, 20 h, and 44%; (e) Na(s), NH_3_, THF/^*t*^BuOH, - 60 °C, 45 min, **3**: 84%, and **7**: 77%; (f) reporter-COOH, Pfp-TFA, DMF; then **3** or **7**, DIPEA, DMF, 24 h. **4**: 7%; **5**: 41%; **6**: 23%; **8**: 8%; **9**: 45%; and **10**: 8%; (g) 8-azido-octyl-1-*O*-triflate,^24^ NaH, THF, −15 °C → rt, 28 h, and 84%; and (h) polymer-bound PPh_3_, AcCN/H_2_O, 70 °C, 5h, and 94%.

To study the reactivity of HypBA1 and *Bt*GH146 toward cyclophellitol aziridines and with the aim to generate activity-based probes with which gut microbiome β-l-arabinofuranosidase activity can be profiled, we decided to prepare β-l-*arabino*furanose-configured cyclophellitol aziridine (**2**) and equip this with various reporters to give a series of potential β-l-arabinofuranosidase ABPs (**8**–**10**, Figure 1). These ABPs are accompanied by the synthesis of O5-modified epoxide probes **4**–**6**. Our studies, which include *in silico* conformational behavior of the epoxide and aziridine scaffolds, structural studies of both enzymes reacted with the nontagged mechanism-based inhibitors, enzyme inhibition kinetic studies, and *in vitro* ABPP experiments, reveal that both enzymes react in the expected fashion with the aziridines (aziridine opening at C1) and that the aziridines are valid ABPs that label both GH families with about equal efficiency.

**Figure 1:**
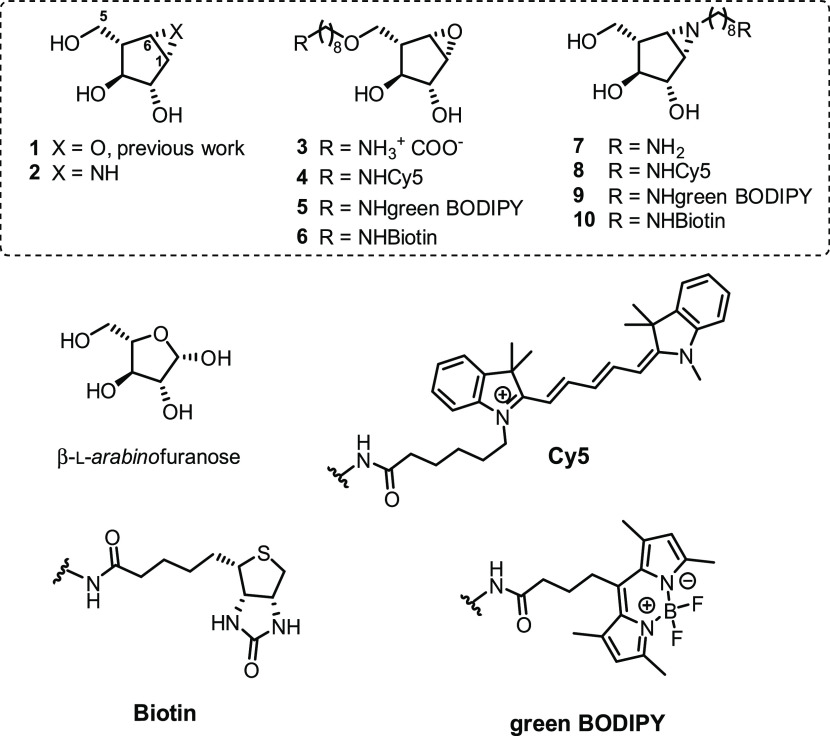
Chemical structures of β-l-*arabino*furanose, β-l-*arabino*furanosyl cyclophellitol epoxides (**3**–**6**) and aziridines (**2** and **7**–**10**) presented in this work and structure of unmodified β-l-*arabino*furanosyl cyclophellitol epoxide **1**(16) as reported previously.

## Results and Discussion

### Conformational Analysis

**Figure 2:**
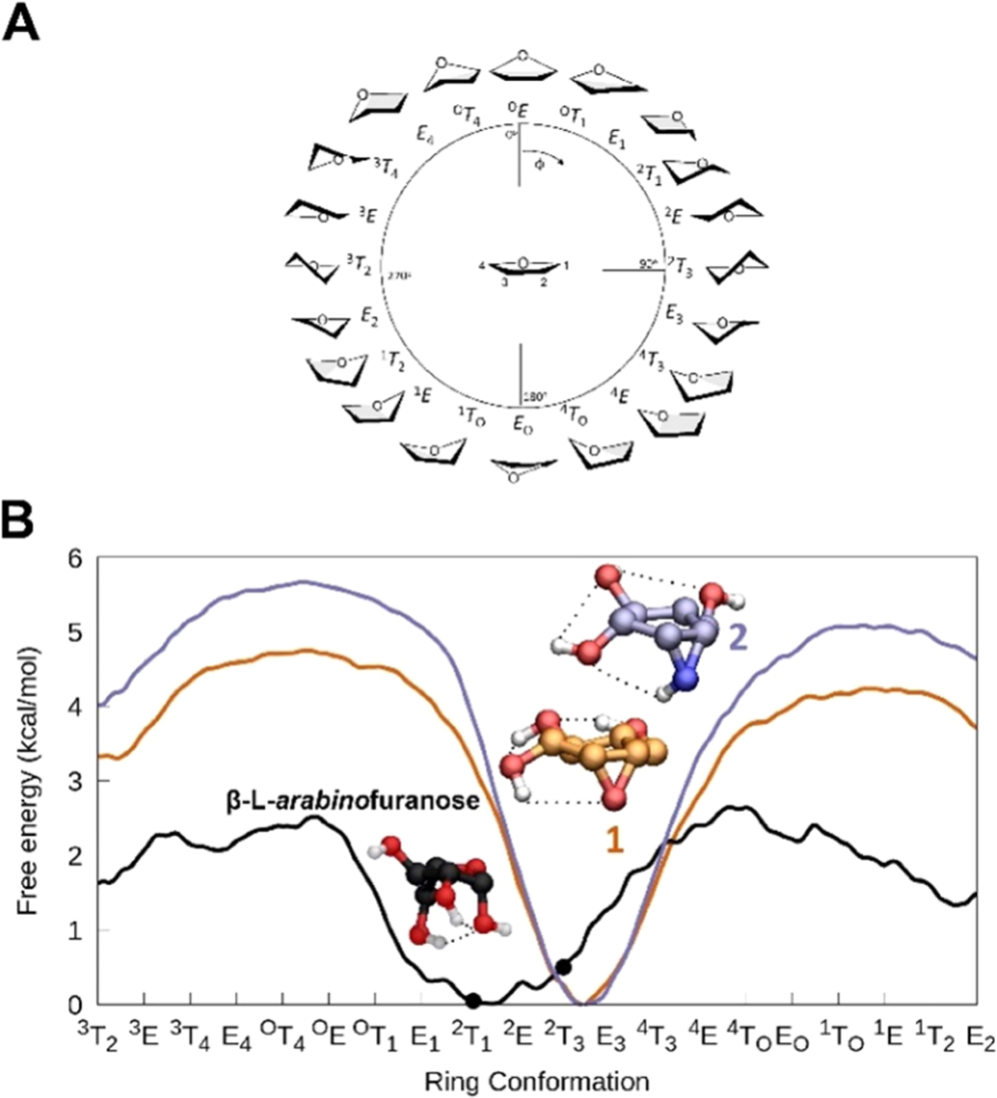
(A) Graphical representation of the conformations of a 5-membered ring according to the Cremer–Pople angle Φ. (B) Conformational free energy profile of isolated β-l-*arabino*furanose (black line), compound **1** (orange line) as previously published,^16^ and compound **2** (blue line). Conformations observed in product complexes of β-l-arabinofuranosidases are represented with a black circle (PDB entry 3WKX for HypBA1; PDB entry 5OPJ for *Bt*GH146).

In the first instance, the conformational free energy profile of the β-l-*arabino*furanosyl cyclitol aziridine (**2**) was computed with *ab initio* metadynamics (Figure 2B) and compared to the ones previously reported for β-l-*arabino*furanosyl cyclophellitol epoxide **1** and for the natural substrate β-l-*arabino*furanose. As illustrated in Figure 2B, the conformational behavior of aziridine **2** is similar to the epoxide (**1**). Both molecules adopt preferably a conformation closely resembling *E*_3_. More energy is needed to twist the conformation of **1** and **2** (free energy = 4.7 and 5.7 kcal/mol, for epoxide and aziridine, respectively) compared to the natural β-l-*arabino*furanose (free energy = ca. 2.6 kcal/mol).

### Synthesis

The synthesis of both epoxide-based and aziridine-based β-l-*arabino*furanosyl cyclitols **2**–**10** involved the installation of the electrophilic trap prior to incorporation of the linker (Scheme 1) and diverged for the two types of warheads at the stage of L-*arabino*furanosyl cyclopentene intermediate **11**. Starting from commercially available α-D-*galacto*pyranoside, bisbenzylated l-*arabino*furanosyl cyclopentene **11** was obtained in nine steps following the synthetic strategy recently reported.^22^l-*Arabino*furanosyl cyclopentene **11** was transformed into the aziridine following a set of stereospecific reactions previously applied to six-membered ring cyclitol structures.^23−25^ Specifically, compound **11** was subjected to trichloroimidation of the unprotected primary hydroxyl functionality followed by stereospecific NIS-induced iodocyclization. The resulting iodotrichloroimidate **12** was hydrolyzed under mild acidic conditions to generate *in situ* the unprotected iodo-ammonium salt, which under mild basic conditions and micromolar concentrations underwent one-pot intramolecular nucleophilic displacement by the primary amine of the adjacent iodine leaving group, affording aziridine **13**.

With bisbenzylated aziridine **13** available, this intermediate was globally deprotected under Birch conditions generating the final β-l-*arabino*furanosyl cyclophellitol aziridine **2**, the aziridine equivalent to unmodified epoxide **1**.^16^ Unsubstituted secondary aziridine **13** was modified by *N*-alkylation with freshly prepared 8-azido-octyl-1-*O*-triflate.^24^ This step was followed by Birch reduction of *N*-alkyl aziridine **14**, which was converted into the water-soluble primary amine **7** by azide reduction and concomitant debenzylation. Probes **8**–**10** were obtained upon formation of an amide linkage between amine **3** and the desired reporter carboxylic acid prior to activation with pentafluorophenyl-trifluoroacetate (Pfp-TFA). The final compounds were purified by HPLC, with the exception of green BODIPY probe **9**, whose relative lipophilicity allowed purification by silica gel flash chromatography.

The synthesis of epoxides **4**–**6** commenced from late-stage epoxide **15** obtained according to the published procedures.^16^ The azido-octyl linker was introduced on the primary hydroxyl functionality by *O*-alkylation with 8-azido-octyl-1-*O*-triflate^24^ under basic conditions, generating azide **16**, which was reduced to primary amine **17** by Staudinger reduction. Reductive cleavage of the benzyl protecting groups in **17** under Birch conditions afforded primary amine **3**, which served both as a prospective inhibitor and as a starting point for the synthesis of ABPs **4**–**6**. Following the same protocol used for the installation of reporters onto aziridine warhead **7**, ABPs **4**–**6** were obtained.

### Inhibition Assays

To assess the effects of the two different electrophilic traps on the inhibitory kinetics of L-*arabino*furanoside-mimicking scaffolds against retaining β-l-arabinofuranosidases, the inhibitory activity of newly developed inhibitors **2** and **7** and probe **4** was assessed and compared with the findings reported for unmodified epoxide **1**. Inhibition kinetics were measured for recombinant HypBA1 and *Bt*GH146 at their optimal pH for catalysis (pH 4.5 for rHypBA1 and pH 7.5 for r*Bt*GH146), using hydrolysis of chromogenic *p*-nitrophenyl β-l-*arabino*furanoside^26^ as a measure of residual enzymatic activity (Table 1).

**Table 1:** rHypBA1 and r*Bt*GH146 Inactivation Rates and Inhibition Constants (*k*_inact_ and *K*_I_) of β-l-*Arabino*furanosyl compounds **1**, **2**, **4**, and **7**

r*Bt*GH146				rHypBA1		
compound	*k*_inact_ (min^–1^)	*K*_I_ (μM)	*k*_inact_/*K*_I_ (M^–1^·s^–1^)	*k*_inact_ (min^–1^)	*K*_I_ (μM)	*k*_inact_/*K*_I_ (M^–1^·s^–1^)
**1**(16)	n.d.^a^	n.d.^a^	<0.5	n.d.^a^	n.d.^a^	14.7
**2**	1.3 ± 0.1	350 ± 40	62	>1	n.d.^b^	>4200
**4**	3.2 × 10^–2^ ± 0.3 × 10^–2^	140 ± 10	3.8	n.d.^a^	n.d.^a^	<0.1
**7**	0.14 ± 0.01	190 ± 20	12	1.3 ± 0.3	280 ± 90	76

a n.d.: not determined due to the limited inhibition observed.

b n.d.: not determined due to the rapid and complete inactivation observed with the lowest concentration of inhibitor tested under the experimental conditions of the assay. To ensure the validity of the pseudo-first-order inhibition kinetic assumption, the lowest concentration of the inhibitor tested was maintained as a ∼ 50-fold excess of inhibitor over enzyme.

Aziridine **2** displayed the highest inhibitory efficacy toward both rHypBA1 and r*Bt*GH146, indicating the broad-spectrum reactivity of β-l-*arabino*furanosyl cyclophellitol aziridines toward β-l-arabinofuranosidases. The different degrees of inhibition rates of **2** with rHypBA1 (GH127) and r*Bt*GH146 may be attributed to structural differences of their binding sites but also to the different pH values at which these enzymes act optimally (and at which the inhibition assays were executed) in relation to the p*K*_aH_ of the aziridine. This p*K*_aH_ is expected to be around pH 7.4, as aziridines in general are at about three points lower p*K*_aH_ compared to nonconstrained aliphatic secondary amines. Also, the presence of three electronegative hydroxyls appended to the pentane ring will lower the p*K*_aH_. Therefore, the expected aziridine p*K*_aH_ likely lies within the physiological pH range (6.0–7.5)^27^ of the human intestine. The intestinal tract represents the predominant colonization site for *B. thetaiotaomicron* and *Bifidobacterium*, which are reported to optimally grow *in vitro* at pH 6.5–7.0.^28,29^ Recently, in growing *B. longum* cultures, a two-unit pH reduction from the typical growth conditions (pH 6.5–7.0) for *Bifidobacterium* was reported upon supplementation of fructooligosaccharide (FOS) in various sugar systems, as a function of FOS concentration and bacterial strain.^29^ This growth habit of *B. longum* suggests that the optimal pH for HypBA1 enzymatic activity may be achieved by the addition of FOS or equivalent nutrients in bacterial growth medium. Irrespective of this hypothesis, the enhanced rate of inhibition of HypBA1 over *Bt*GH146 in the study presented here (Table 1) may also be due to the greater degree of protonation (and so activation) of the aziridine, already outside the enzyme active site, under the significantly more acidic conditions at which HypBA1 (pH optimum of 4.5) activity is measured than that of *Bt*GH146 (pH optimum of 7.5).

To complement the kinetic assessment, epoxides **3**–**6** and aziridines **2** and **7** were analyzed by intact mass spectrometry (Supporting Figure S2) following incubation with rHypBA1 and r*Bt*GH146. Covalent adduct formation was observed for all tested compounds after 16 h of incubation, and a covalent adduct was detected with aziridines **2** and **7** after 1 h for rHypBA1. RHypBA1 thus reacted faster with β-l-*arabino*furanosyl cyclitol aziridines compared with the epoxides. For aziridines **2** and **7**, intact MS analysis upon incubation with rHypBA1 resulted in an observed mass difference (+146.2 for **2** and +273.8 for **7**) differing by one unit from the expected values (+145 for **2** and +272 for **7**). The HypBA1 binding cleft differs from that of *Bt*GH146 in having a nonconserved cysteine residue (C415) positioned in proximity to the catalytic nucleophile C417. The reported mass difference for rHypBA1 might account for a change in the protonation state of this additional nonconserved thiol, which may undergo protonation under the test conditions. An alternative hypothesis is that one of the noncovalently modified conserved cysteines (C418, C340) may lose coordination to the zinc cation, as a result of protein denaturation during the intact mass spectrometry analysis.

### Ligand–Enzyme Interactions by **2** with rHypBA1 and r*Bt*GH146

**Figure 3:**
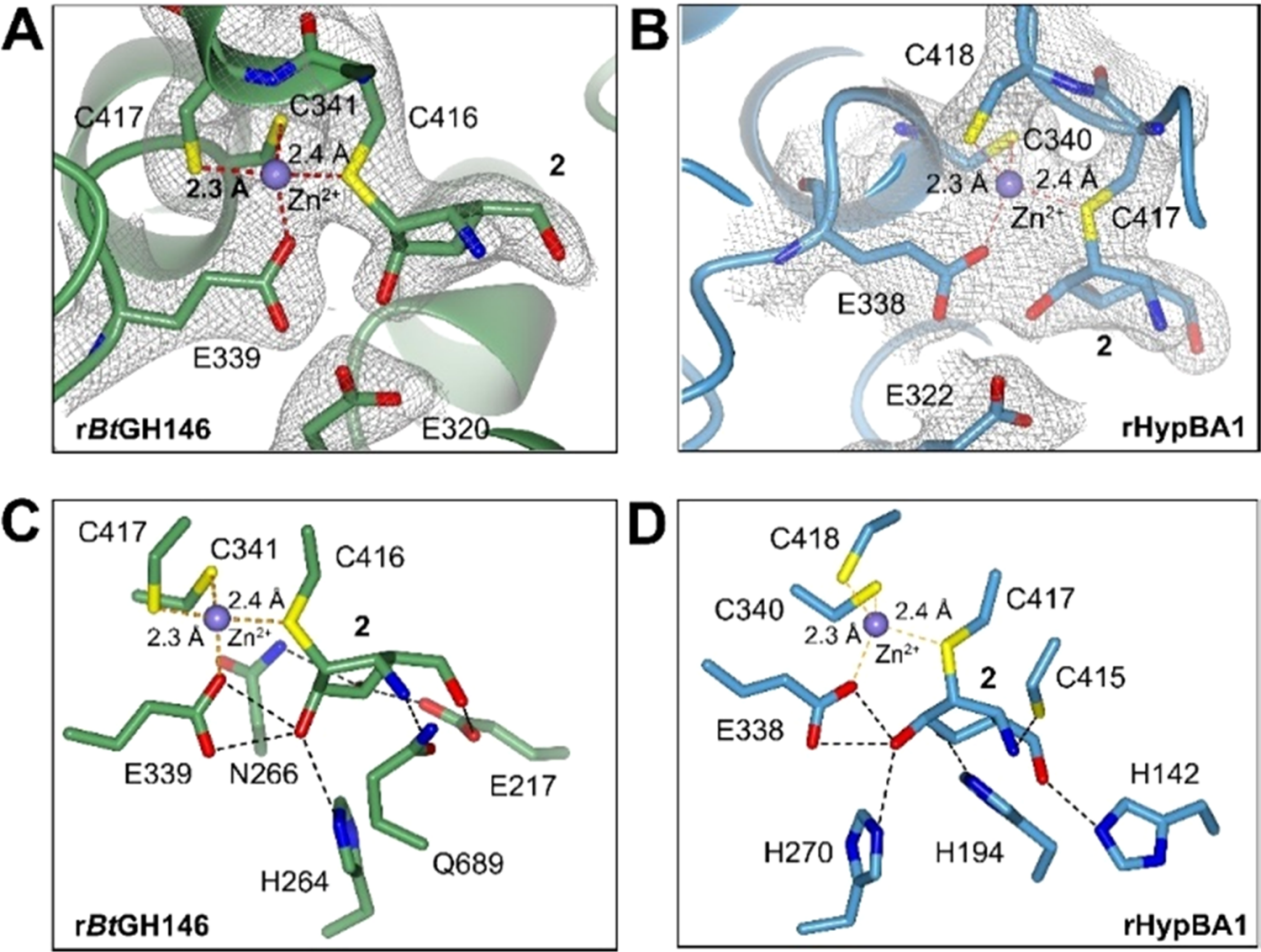
(A) Active site structure of r*Bt*GH146 upon reaction with **2** revealing electron density indicative of a covalent bond between C1 and C416. The 2*F*_0_–*F*_c_ map is shown contoured to 1σ for key residues, and bonds coordinating the zinc ion are depicted as dashed orange lines. (Please see notes on residue numbering for r*Bt*GH146 in the Supporting Information.) (B) Active site structure of rHypBA1 bound to **2**, with density calculated for **2** and C417, and coordination shown as in panel A. (C) Active site structure of r*Bt*GH146 upon reaction with **2** showing hydrogen bond interactions between **2** and the active site of r*Bt*GH146, with hydrogen bonds shown as black dashed lines and coordination to the zinc ion as dashed orange lines with distances in angstroms. (D) Active site structure of rHypBA1 bound to **2**, with hydrogen bonds and coordination shown as in panel C.

In order to gain structural insights into the reactivity of aziridine **2** with rHypBA1 and r*Bt*GH146, it was cocrystallized with both enzymes, and the resulting complexes were studied by X-ray diffraction. Electron density for the carbon emulating the anomeric center (C1) in aziridine **2** was observed at covalent bond distance from the catalytic nucleophile residue in both isozymes (C416 for r*Bt*GH146 in Figure 3A; C417 for rHypBA1 in Figure 3B). Inspection of the network of H-bond interactions by **2** in complex with rHypBA1 and r*Bt*GH146 revealed that the primary hydroxyl O5 and the transdiaxially opened aziridine group interact differently with the active sites of the two isozymes. In particular, the primary hydroxyl O5 in **2** was involved in H-bond interactions with distinct H-bond acceptor residues within the binding pocket—these are E217 in r*Bt*GH146:**2** (Figure 3C) and H142 in rHypBA1:**2** (Figure 3D). The amino group of the opened aziridine in **2** displayed H-bond interactions with the thiol group of nonconserved cysteine C415 in rHypBA1:**2**. However, no cysteine equivalent to C415 of rHypBA1 is present in the active site of r*Bt*GH146. In the r*Bt*GH146:**2** complex, N1 of **2** interacts exclusively with a proximal glutamine (Q689) in the active site. The presence of a nonconserved cysteine in rHypBA1, which interacts with the amino moiety of the opened aziridine, may explain the superior reactivity of **2** with rHypBA1 over r*Bt*GH146.

The covalent complex of r*Bt*GH146 with aziridine **2** displayed the anomeric-mimicking carbon C1 at covalent bond distance to the thiol group of C416. This points to the expected regiochemical outcome as for conventional retaining glycosidases^30−34^ and provides further evidence supporting annotation of this enzyme, like rHypBA1, as a retaining β-l-arabinofuranosidase utilizing a cysteine catalytic site nucleophile. However, there is a significant difference in the mode of action toward aziridine **2** and epoxide **1**. When combining the inhibitory kinetic findings with the predicted *in silico* conformational analysis, we conclude that the observed differences in inactivation kinetics and regiochemical outcomes between **1** and **2** with r*Bt*GH146 cannot be ascribed to their conformational behavior. Finally and as also discussed above (enzyme inhibition assay section), the difference in reactivity may also originate in the p*K*_aH_ values of the respective inhibitors, in combination with the enzyme pH optimum.

### Activity-Based Protein Profiling

**Figure 4:**
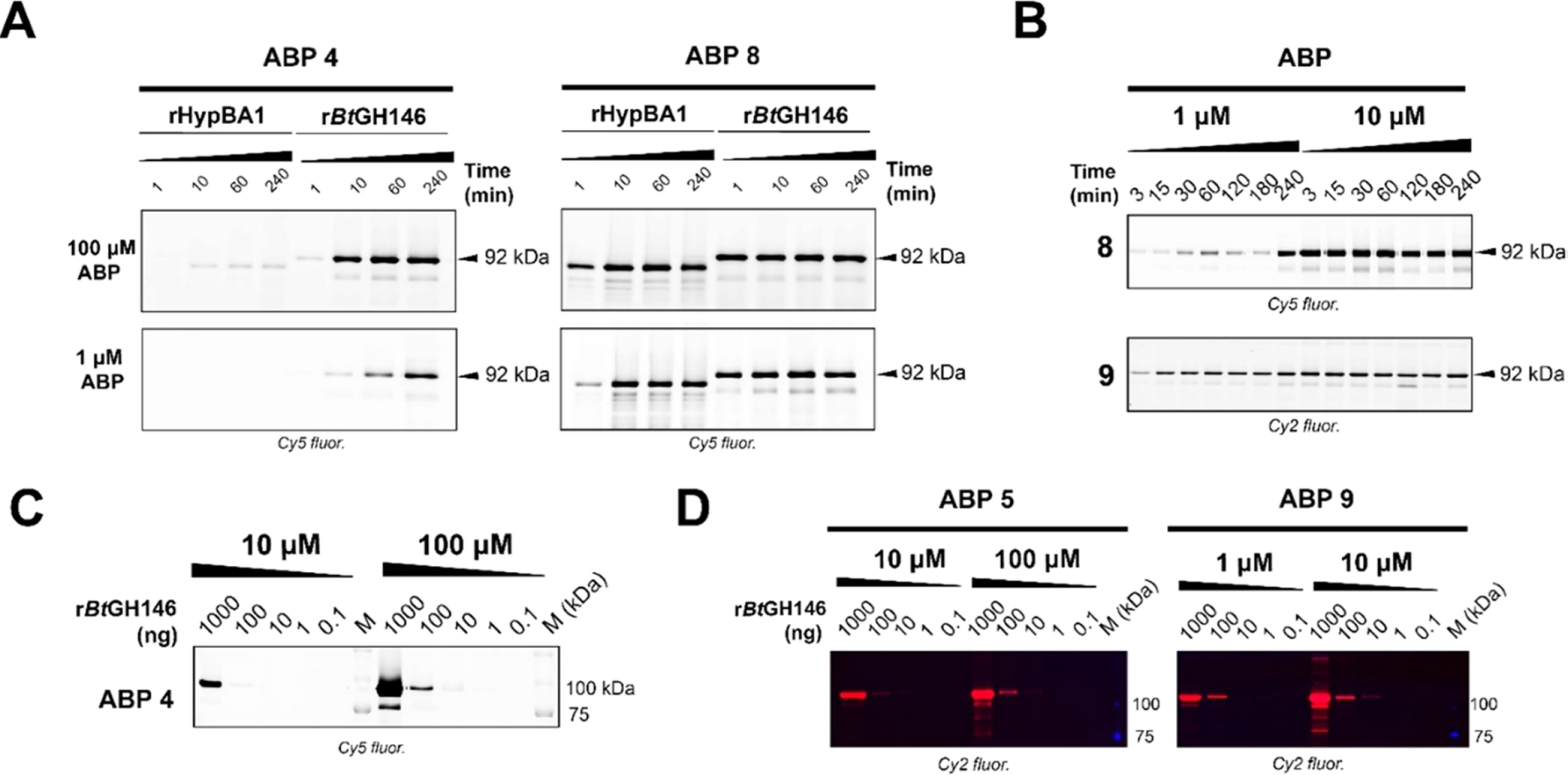
(A) Time-dependent fluorescence labeling of rHypBA1 and r*Bt*GH146 by epoxide-armed Cy5-tagged ABP **4** (left) or aziridine-armed Cy5-tagged ABP **8** (right) at 1 or 100 μM final ABP concentration during 4 h of incubation at optimal enzyme pH (rHypBA1: pH 4.5 NaOAc, 1 mM DTT; r*Bt*GH146: pH 7.0 NaPi, 1 mM DTT), showing a faster and more effective labeling of both rHypBA1 and r*Bt*GH146 by **8** compared to epoxide-based ABP **4** within the tested time window. (B) Fluorescence labeling of r*Bt*GH146 (100 ng) by Cy5-tagged ABP **8** (above) and green Bodipy-tagged ABP **9** (below) after 1 h of incubation at pH 7.4 (PBS/NaCl) and 1 or 10 μM final probe concentration, with ABP **9** displaying faster and more effective labeling of the tested enzyme than **8** at 1 μM ABP concentration. (C) Detection sensitivity of Cy5-tagged ABP **4** toward r*Bt*GH146 after 1 h of incubation at pH 7.5 (HEPES/NaCl), with 10 and 100 μM final probe concentrations. (D) Detection sensitivity of green Bodipy-tagged epoxide-type ABP **5** and aziridine-type ABP **9** toward r*Bt*GH146 after 1 h of incubation (with **5**: at pH 7.5 HEPES/NaCl; with **9**: pH 7.4 PBS/NaCl), showing the lower r*Bt*GH146 detection limit of aziridine-type probe **9** with respect to **5** under the tested conditions. We note the presence of a lower running (kDa of about 75) band in the r*Bt*GH146 labeling experiments. While we have no evidence, we hypothesize that these bands may be partially proteolyzed (by the expression host, *E. coli*) yet still active enzyme, considering that the full-length enzyme has a floppy, proteolysis-prone lid domain.

We next investigated the labeling efficiency of both recombinant isozymes (Figure 4A) for Cy5 aziridine **8** and Cy5 epoxide ABP **4**. For this purpose, samples of the recombinant enzyme were treated with either of the probes at varying concentrations and times, after which the samples were denatured and resolved on SDS-PAGE and the ABP-labeled proteins visualized by fluorescence scanning of the wet gel slabs. In agreement with the difference in kinetic parameters determined for aziridine **7** and epoxide **4**, ABP **8** labeled both tested isozymes more rapidly and more potently than ABP **4**. ABP **4** labeled r*Bt*GH146 effectively after 4h of ABP incubation, whereas no equivalent band was detected for rHypBA1 (74 kDa) within the tested incubation time window. These results are in accordance with the *in vitro* inhibition assessments. As shown in Figure 4B, complete fluorescent labeling of r*Bt*GH146 (92 kDa) can be achieved both by Cy5 ABP **8** and green BODIPY ABP **9** upon 4 h of ABP incubation at 1 μM ABP, and fluorescence bands could be detected after as little as 3 min of incubation with either ABPs by increasing the ABP concentration 10-fold. In contrast, no significant fluorescence band corresponding to the expected mass of r*Bt*GH146 (92 kDa) was observed after 4 h of incubation with epoxide ABPs **4** or **5** at 10 μM concentration using lower amounts than 1000 ng of enzyme. However, increasing the ABP concentration by 1 order of magnitude (100 μM) afforded an approximately 10-fold decrease in the detection limit (100 ng rBtGH146). Green BODIPY-tagged aziridine **9** (1 μM ABP) enabled fluorescence detection of r*Bt*GH146 down to 100 ng of enzyme (Figure 4D), that is, 10-fold lower enzyme amounts than with an epoxide ABP. This points to the utility of aziridine-type chemical tools for profiling of retaining β-l-arabinofuranosidases, where no selectivity for either GH127 or GH146 isozymes needs to be applied. In contrast, β-l-*arabino*furanosyl-configured epoxide-type probes were found to react preferentially with r*Bt*GH146, which may be due to the different reactivity (or p*K*_aH_) of the epoxide versus that of the aziridine but also because of the position of the reporter tag (O5 versus aziridine).

## Conclusions

This work reports on the synthesis and *in vitro* biochemical evaluation of a panel of β-l-*arabino*furanosyl cyclophellitol epoxides and aziridines as covalent inhibitors and activity-based probes for retaining β-l-arabinofuranosidases from GH families 127 and 146. Development of these chemical scaffolds builds on the recent discovery^16^ of the enzymatic mechanism adopted by r*Bt*GH146 and rHypBA1, as elucidated by the use of β-l-*arabino*furanosyl cyclophellitol epoxide **1**. Building on the mechanism of action inferred from the previous crystallographic studies conducted with epoxide **1** reacted with r*Bt*GH146 and rHypBA1,^16^ the regiochemical outcome of the catalytic reactions of both enzymes with aziridine **2** was explored by 3D crystallographic studies. We found that aziridine **2** reacts at the conventional position, that is, the carbon equivalent to the anomeric center in β-l-arabinofuranoside, with r*Bt*GH146 as well as rHypBA1. This result contrasts with our previous results on epoxide **1**, which when reacted with r*Bt*GH146 was found to ring-open at the “wrong” epoxide carbon. We believe the enhanced reactivity of the aziridines, when compared to epoxide **1**, may be behind these findings, and while we have no satisfactory explanation for this finding, we do believe that the *Bt*GH146 reaction with the aziridines better reflects its mode of action on natural substrates. Our gel-based ABPP experiments further show the utility of our β-l-*arabino*furanosyl cyclophellitol aziridines as *bonafide* β-l-arabinofuranosidase probes and form, in our opinion, a valuable addition to the growing activity-based glycosidase probe portfolio for the discovery of such enzymes in an unbiased fashion from various biological sources.

## Methods

### Chemical Synthesis

All chemicals were purchased from Sigma-Aldrich unless otherwise specified. L-*Arabino*cyclopentene **11**(22) and bisbenzylated β-l-*arabino*furanosyl epoxide **15**(16) and were synthesized at the Bio-organic Synthesis, Leiden Institute of Chemistry at Leiden University, according to the published methods. Synthetic methods and NMR characterization of newly synthesized compounds can be found in the Supporting Information.

### Recombinant Enzyme Production

*Bt*GH146 and HypBA1 were produced and purified according to the published procedures.^13,16^

### Intact Mass Spectrometry

r*Bt*GH146 was diluted to 1 mg mL^–1^ in an SEC buffer. Compounds **2** – **7** (0.1 mM final concentration) were added to the enzyme solution, and the reactions were incubated at 37 °C for different time periods. 2 μL samples were taken after 1 or 16 h. These samples (2 μL) were then diluted with 48 μL of 1% formic acid and 10% acetonitrile and analyzed as described previously. rHypBA1 was diluted to 0.1 mg mL^–1^ in 50 mM NaPi (pH 4.5), and compounds **2**–**7** (0.1 mM final concentration) were added. 10 μL of samples was taken after 60 and 960 min. These aliquots (10 μL) were diluted with 10 μL of 1% formic acid and 10% acetonitrile and stored at −20 °C until intact MS could be performed in the same manner as for the samples treated with r*Bt*GH146.

### Enzyme Inhibition Kinetics

rHypBA1 (20 mg mL^–1^ stored at −80 °C) was freshly thawed and diluted to 1 mg mL^–1^ in 50 mM acetate (pH 4.5) supplemented with 1 mM DTT (assay buffer). Inhibitors **2**, **4**, and **7** were dissolved in water at 5 mM concentration and used to prepare a dilution series from 1 mM to 16 μM in the assay buffer alongside a buffer control without an inhibitor. A working enzyme solution was prepared at 20 μg/mL in assay buffer. 35 μL of enzyme solution was added to 35 μL of prewarmed inhibitor solution, and the inhibition reactions were incubated at 37 °C. Aliquots (7.5 μL) of these inactivation mixtures were removed at time intervals (5, 10, 20, 30, 40, and 60 min of incubation) and diluted with 142.5 μL of prewarmed 0.25 mM 4-nitrophenyl-β-l-*arabino*furanoside^26^ in the assay buffer and then incubated at 37 °C. Aliquots (40 μL) of the resulting samples were taken from each substrate hydrolysis reaction at defined time intervals (1, 3, and 8 min of incubation) and mixed with 40 μL of stop solution (200 μM Na_2_CO_3_) in a 384-well plate. Absorbance change at 405 nm wavelength (A405) was read by using an Epoch plate reader (Biotek). Hydrolysis rates were determined as the slope of a linear fit of A405 versus time. Slope values were converted into rates using a 40 μL calibration series of 4-nitrophenol in assay buffer mixed with 40 μL of stop solution. To account for slow enzyme activity loss in the no-inhibitor control, rates were converted into residual activity by dividing each measured rate by the inhibitor-free hydrolysis rate at that incubation point. Residual activities were then plotted against incubation time using OriginPro graphing software (OriginLab) for each inhibitor concentration and fitted with exponential decay curves having y offset (y0) values fixed at 0 (with the exception of the uninhibited rates where y0 = 1). Extracted apparent decay constant (*k*_app_) values were then plotted against the concentration of compounds **2**, **4**, and **7** in each inhibition reaction with error estimates taken as the standard error from the exponential decay fit. Since no inflection was observed in the *k*_app_ vs [I] curve, an error-weighted linear fit was performed to determine *k*_inact_/*K*_I_.

r*Bt*GH146 (20 mg mL^–1^ stored at −80 °C) was freshly thawed and diluted to 1 mg mL^–1^ in 50 mM NaPi at pH 7.0 with the assay buffer (1 mM DTT, 20 mM HEPES (pH 7.5) supplemented with 200 mM NaCl). Inhibitors **2**, **4**, and **7** were dissolved in water at 5 mM concentration and used to prepare a dilution series from 1 mM to 16 μM in the assay buffer alongside a buffer control without an inhibitor. A working enzyme solution was prepared at 20 μg/mL in assay buffer. The subsequent steps were the same as those for the procedure described for HypBA1 mentioned above.

### ABP Labeling of rHypBA1 and r*Bt*GH146 by **4**

rHypBA1 and r*Bt*GH146 were labeled under optimum conditions. ABP **4** (10 mM stock, 100% w/v DMSO) was diluted with Milli-Q water to prepare a 1 mM ABP working solution (10% w/v). The enzyme stock (HypBA1:20 mg mL^–1^ HypBA1 in 20 mM MOPS, pH 7.0, 1 mM DTT; *Bt*GH146:1 mg mL^–1^ in 20 mM MOPS, pH 7.5) was thawed on ice. The assay buffer for rHypBA1 was prepared by mixing 500 μL of 1 M NaOAc, 50 μL of 1 M DTT, and 9.45 mL of deionized water. For *Bt*GH146 labeling, the assay buffer (pH 7.0, NaPi, 1 mM DTT) was prepared with deionized water. The enzyme stock (10 μL) was diluted to 0.01 mg mL^–1^ enzyme concentration with the assay buffer (990 μL assay buffer). Aliquots (100 μL) of the enzyme working solution were heated to 95 °C for 5 min to inactivate the enzyme solution. To both the working and inactivated enzyme solutions (90 μL each) was added the ABP working solution (**4**, 10 μL) to achieve a final ABP concentration of 0.1 mM. The samples were incubated at 37 °C. At regular time intervals (0, 10, 60, 240 min), 15 μL samples were taken and mixed with 5 μL of 4× SDS-PAGE loading buffer (40% glycerol, 4% SDS, 250 mM Tris-HCl, pH 6.8, 10% 2-mercaptoethanol, 0.2 mg of mL^–1^ bromophenol blue). The samples were immediately heated to 95 °C for 2 min and then stored frozen (−20 °C) until ready for analysis. Wet slab gels were scanned for fluorescence using a Typhoon FLA 9500 (GE Healthcare) at λ_EX_ 635 nm and λ_EM_ ≥ 665 nm for Cy5-tagged ABP **4**.

### ABP Labeling of r*Bt*GH146 by **4**, **5**, or **9**

To prepare for labeling, recombinant *Bt*GH146 stock (1 mg mL^–1^ in 20 mM MOPS pH 7.5) was diluted with assay buffer (for ABPs **4**: 20 mM HEPES 150 mM NaCl, pH 7.5; for ABPs **5** and **9**: PBS 150 mM NaCl, pH 7.4) to varying enzyme concentrations (111, 11, 1, 0.1, and 0.01 μg/mL). ABP **4**, **5**, and **9** stocks (10 mM in DMSO) were diluted with assay buffer to 1 mM and 0.1 mM ABP concentrations (for **4** and **5**) or to 100 μM and 10 μM (for ABP **9**). For labeling, 9 μL of each of the enzyme working solutions was loaded in separate Eppendorf tubes on ice to yield a total final amount of 1000, 100, 10, 1, and 0.1 ng of *Bt*GH146 per reaction. The enzyme solution was thus incubated with 1 μL of ABP (**4**, **5**, or **9**) at 37 °C for 1 h (Figure 4B and 4C). Samples were then denatured with 2.66 μL of sample buffer (5x Laemmli buffer, containing 50% (v/v) 1 M Tris-HCl pH 6.8, 50% (v/v) glycerol, 10% (w/v) dithiothreitol (DTT), 10% (w/v) sodium dodecyl sulfate (SDS), 0.01% bromophenol blue) and heated at 98 °C for 5 min. Proteins were resolved by electrophoresis in sodium dodecyl sulfate (SDS-PAGE) 10% polyacrylamide gels, and wet slab gels were scanned as described above. Wet slab gels were scanned for fluorescence using a Typhoon FLA 9500 (GE Healthcare) at λ_EX_ = 635 and λ_EM_ ≥ 665 nm for Cy5-tagged ABP **4** and **9** and at λ_EX_ = 473 and λ_EM_ ≥ 510 nm for green BODIPY-tagged ABP **5**.

### Time-Dependent Labeling of r*Bt*GH146 by **8**

Recombinant *Bt*GH146 stock (1 mg mL^–1^ in 20 mM MOPS pH 7.5) was diluted with assay buffer (PBS 150 mM NaCl, pH 7.4), giving a 11 μg/mL working enzyme solution. Cy5-tagged ABP **8** (10 mM in DMSO) was diluted with the assay buffer to 100 and 10 μM ABP concentrations. For labeling, 9 μL of enzyme working solution (100 ng of total *Bt*GH146 in the final reaction mixture) was loaded in separate Eppendorf tubes on ice, and 1 μL of ABP working solution was added to each tube. Reactions were incubated at 37 °C while shaking. At time intervals (3, 15, 30, 60, 120, 180, 240 min), samples were denatured with 5× Laemmli buffer, boiled at 98 °C for 5 min, and subjected to SDS-PAGE and fluorescence scan as described above.

### Crystallization of rHypBA1 or r*Bt*GH146 and Soaking with **2**

Information on the crystallization of the purified enzymes and soaking of the crystals with inhibitor **2** can be found in the Supporting Information, together with the procedures used for X-ray data collection and structure solution.^35−48^

### Conformational Free Energy Landscape of **2** in Vacuum

The conformational free energy landscape (FEL) was computed for β-l-*arabino*furanose cyclophellitol aziridine (**2**) using density functional theory-based molecular dynamics (MD), according to the Car–Parrinello (CP) method.^49^ The molecule was enclosed in an isolated cubic box of 12.5 Å^3^. A fictitious electron mass of 500 atomic units (au) was used for the CP Lagrangian, and a time step of 0.12 fs was used in all CPMD simulations. This is the same setup used in previous work on β- and α-l-*arabino*furanose inhibitors.^16,22^ The Kohn–Sham orbitals were expanded in a plane wave basis set with a kinetic energy cutoff of 70 Ry. Ab initio pseudopotentials, generated within the Troullier–Martins scheme,^50^ were employed. The Perdew, Burke, and Ernzerhoff (PBE)-generalized gradient-corrected approximation^51^ was selected. The metadynamics algorithm,^52^ provided by the Plumed 2 plugin,^53^ was used to explore the conformational FEL of the systems, taking as collective variables (CVs) the pseudorotational-phase (φ) puckering coordinate^54^ as well as a dihedral angle accounting for the rotation of the sugar hydroxymethyl group. The energy was projected into the φ coordinate for representation purposes. The height of these Gaussian terms was set at 0.6 kcal/mol, and a new Gaussian-like potential was added every 500 MD steps. To ensure convergence at the last 100 ps, the Gaussian height was decreased to 0.2 kcal/mol and the pace was set to 1000 MD steps. The widths of the CVs were set to 0.035 and 0.1 rad for φ and the hydroxymethyl dihedral angle, respectively, according to the oscillations of the CVs in the free dynamics. The simulations were stopped when energy differences among wells remain constant and a diffusive behavior was observed in both CVs, which was further confirmed by a time-independent free energy estimator.^55^ The energy error, taken from the standard deviation within the last 30 ps, is below 0.6 kcal/mol. The exploration of the phase space was extended up to 300 ps, which corresponds to 4000 added Gaussian functions.

## Supporting Information Available

Synthetic procedures, characterization of compounds, biochemical inhibition data, intact mass spectrometry, ^1^H and ^13^C NMR spectra for all newly synthesized compounds, X-ray data processing, and refinement statistics (PDF)

## Accession Codes

Coordinates and structure factors have been deposited with the Protein Data Bank under the accession numbers 8QF2 and 8QF8.

## Abbreviations

*Bt*: *Bacteroides thetaiotaomicron*
GH: glycosyl hydrolase
RG-II: rhamnogalacturonan II
FOS: fructooligosaccharide

## References

[ref1] Ndeh D.; Rogowski A.; Cartmell A.; Luis A. S.; Baslé A.; Gray J.; Venditto I.; Briggs J.; Zhang X.; Labourel A.; Terrapon N.; Buffetto F.; Nepogodiev S.; Xiao Y.; Field R. A.; Zhu Y.; O’Neil M. A.; Urbanowicz B. R.; York W. S.; Davies G. J.; Abbott D. W.; Ralet M.-C.; Martens E. C.; Henrissat B.; Gilbert H. J. Complex pectin metabolism by gut bacteria reveals novel catalytic functions. Nature 2017, 544, 65–70. 10.1038/nature21725. 28329766 PMC5388186

[ref2] Kieliszewski M. J.; Lamport D. T. Extensin: repetitive motifs, functional sites, post-translational codes, and phylogeny. Plant J. 1994, 5, 157–172. 10.1046/j.1365-313X.1994.05020157.x. 8148875

[ref3] Kieliszewski M. J.; Showalter A. M.; Leykam J. F. Potato lectin: a modular protein sharing sequence similarities with the extensin family, the hevein lectin family, and snake venom disintegrins (platelet aggregation inhibitors). Plant J. 1994, 5, 849–861. 10.1046/j.1365-313X.1994.5060849.x. 8054990

[ref4] Kaneko S.; Sano M.; Kusakabe I. Purification and some properties of alpha-L-arabinofuranosidase from *Bacillus subtilis* 3–6. Appl. Environ. Microbiol. 1994, 60, 3425–3428. 10.1128/aem.60.9.3425-3428.1994. 7944374 PMC201823

[ref5] Komeno M.; Hayamizu H.; Fujita K.; Ashida H. Two Novel α-l-Arabinofuranosidases from *Bifidobacterium longum* subsp. longum Belonging to Glycoside Hydrolase Family 43 Cooperatively Degrade Arabinan. Appl. Environ. Microbiol. 2019, 85, e02582–18. 10.1128/AEM.02582-18. 30635377 PMC6414367

[ref6] Schell M. A.; Karmirantzou M.; Snel B.; Vilanova D.; Ber-ger B.; Pessi G.; Zwahlen M.-C.; Desiere F.; Bork P.; Delley M.; Prid-more R. D.; Arigoni F. The genome sequence of Bifidobacterium longum reflects its adaptation to the human gastrointestinal tract. Proc. Natl. Acad. Sci. U.S.A. 2002, 99, 14422–14427. 10.1073/pnas.212527599. 12381787 PMC137899

[ref7] Margolles A.; de los Reyes-Gavilán C. G. Purification and Functional Characterization of a Novel α-L-Arabinofuranosidase from *Bifidobacterium longum* B667. Appl. Environ. Microbiol. 2003, 69, 5096–5103. 10.1128/AEM.69.9.5096-5103.2003. 12957891 PMC194971

[ref8] Komeno M.; Yoshihara Y.; Kawasaki J.; Nabeshima W.; Maeda K.; Sasaki Y.; Fujita K.; Ashida H. Two α-l-arabinofuranosidases from *Bifidobacterium longum* subsp.*longum* are involved in arabinoxylan utilization. Appl. Microbiol. Biotechnol. 2022, 106, 1957–1965. 10.1007/s00253-022-11845-x. 35235007

[ref9] Wardman J. F.; Bains R. K.; Rahfeld P.; Withers S. G. Carbohydrate-active enzymes (CAZymes) in the gut microbiome. Nat. Rev. Microbiol. 2022, 20, 542–556. 10.1038/s41579-022-00712-1. 35347288

[ref10] Arzamasov A. A.; van Sinderen D.; Rodionov D. A. Comparative genomics reveals the regulatory complexity of bifidobacterial arabinose and arabino-oligosaccharide utilization. Front. Microbiol. 2018, 9, 776 10.3389/fmicb.2018.00776. 29740413 PMC5928203

[ref11] Fujita K.; Takashi Y.; Obuchi E.; Kitahara K.; Suganuma T. Characterization of a novel β-L-arabinofuranosidase in *Bifidobacterium longum*: functional elucidation of a DUF1680 protein family member. J. Biol. Chem. 2014, 289, 5240–5249. 10.1074/jbc.M113.528711. 24385433 PMC3931080

[ref12] Ito T.; Saikawa K.; Kim S.; Fujita K.; Ishiwata A.; Kaeothip S.; Arakawa T.; Wakagi T.; Beckham G. T.; Ito Y.; Fushinobu S. Crystal structure of glycoside hydrolase family 127 β-l-arabinofuranosidase from *Bifidobacterium longum*. Biochem. Biophys. Res. Commun. 2014, 447, 32–37. 10.1016/j.bbrc.2014.03.096. 24680821

[ref13] Maruyama S.; Sawano K.; Amaki S.; Suzuki T.; Narita S.; Kimura K.; Arakawa T.; Yamada C.; Ito Y.; Dohmae N.; Fujita K.; Ishiwata A.; Fushinobu S. Substrate complex structure, active site labeling and catalytic role of the zinc ion in cysteine glycosidase. Glycobiology 2022, 32, 171–180. 10.1093/glycob/cwab103. 34735571

[ref14] Zhu Z.; He M.; Huang C.-H.; Ko T.-P.; Zeng Y.-F.; Huang Y.-N.; Shiru Jia F.; Lu J.-R.; Liu R.-T.; Guo Crystal structure of glycoside hydrolase family 127 β-l-arabinofuranosidase from *Bifidobacterium longum*. Acta Crystallogr., Sect. F: Struct. Biol. Commun. 2014, 70, 636–638. 10.1107/S2053230X14001812. 24817727 PMC4014336

[ref16] McGregor N. G. S.; Coines J.; Borlandelli V.; Amaki S.; Artola M.; Nin-Hill A.; Linzel D.; Yamada C.; Arakawa T.; Ishiwata A.; Ito Y.; van der Marel G. A.; Codée J. D. C.; Fushinobu S.; Overkleeft H. S.; Rovira C.; Davies G. J. Cysteine Nucleophiles in Glycosidase Catalysis: Application of a Covalent β-l-Arabinofuranosidase Inhibitor. Angew. Chem., Int. Ed. 2021, 60, 5754–5758. 10.1002/anie.202013920. 33528085

[ref17] Speciale G.; Thompson A. J.; Davies G. J.; Williams S. J. Dissecting conformational contributions to glycosidase catalysis and inhibition. Curr. Opin. Struct. Biol. 2014, 28, 1–13. 10.1016/j.sbi.2014.06.003. 25016573 PMC4220041

[ref18] Davies G. J.; Planas A.; Rovira C. Conformational analyses of the reaction coordinate of glycosidases. Acc. Chem. Res. 2012, 45, 308–316. 10.1021/ar2001765. 21923088

[ref19] Wu L.; Armstrong Z.; Schröder S. P.; de Boer C.; Artola M.; Aerts J. M. F. G.; Overkleeft H. S.; Davies G. J. An overview of activity-based probes for glycosidases. Curr. Opin. Chem. Biol. 2019, 53, 25–36. 10.1016/j.cbpa.2019.05.030. 31419756

[ref20] Rempel B. P.; Withers S. G. Covalent inhibitors of glycosidases and their applications in biochemistry and biology. Glycobiology 2008, 18, 570–586. 10.1093/glycob/cwn041. 18499865

[ref21] Premkumar L.; Sawkar A. R.; Boldin-Adamsky S.; Toker L.; Silman I.; Kelly J. W.; Futerman A. H.; Sussman J. L. X-ray Structure of Human Acid-β-Glucosidase Covalently Bound to Conduritol-B-Epoxide. J. Biol. Chem. 2005, 280, 23815–23819. 10.1074/jbc.M502799200. 15817452

[ref22] McGregor N. G. S.; Artola M.; Nin-Hill A.; Linzel D.; Haon M.; Reijngoud J.; Ram A.; Rosso M.-N.; van der Marel G. A.; Codée J. D. C.; van Wezel G. P.; Berrin J.-G.; Rovira C.; Overkleeft H. S.; Davies G. J. Rational Design of Mechanism-Based Inhibitors and Activity-Based Probes for the Identification of Retaining α-l-Arabinofuranosidases. J. Am. Chem. Soc. 2020, 142, 4648–4662. 10.1021/jacs.9b11351. 32053363 PMC7068720

[ref23] Li K.; Jiang J.; Witte M.; Kallemeijn W. W.; van den Elst H.; Wong C.; Chander S.; Hoogendoorn S.; Beenakker T. J. M.; Codée J. D. C.; Aerts J. M. F. G.; van der Marel G. A.; Overkleeft H. S. Eur. J. Org. Chem. 2014, 2014, 6030–6043. 10.1002/ejoc.201402588.

[ref24] Schröder S. P.; van de Sande J. W.; Kallemeijn W. W.; Kuo C.-L.; Artola M.; van Rooden E. J.; Jiang J.; Beenakker T. J. M.; Florea B. I.; Offen W. A.; Davies G. J.; Minnaard A. J.; Aerts J. M. F. G.; Codée J. D. C.; van der Marel G. A.; Overkleeft H. S. Synthesis of cyclophellitol, cyclophellitol aziridine, and their tagged derivatives. Chem. Commun. 2017, 53, 12528–12531. 10.1039/C7CC07730K. 29116266

[ref25] Jiang J. B.; Kallemeijn W. W.; Wright D. W.; van den Nieuwendijk A. N. C. H.; Rohde V. C.; Folch E. C.; van den Elst H.; Florea B. I.; Scheij S.; Donker-Koopman W. E.; Verhoek M.; Li N.; Schurmann M.; Mink D.; Boot R. G.; Codée J. D. C.; van der Marel G. A.; Davies G. J.; Aerts J. M. F. G.; Overkleeft H. S. In vitro and in vivo comparative and competitive activity-based protein profiling of GH29 α-l-fucosidases. Chem. Sci. 2015, 6, 2782–2789. 10.1039/C4SC03739A. 29142681 PMC5654414

[ref26] Kaeothip S.; Ishiwata A.; Ito T.; Fushinobu S.; Fujita K.; Ito Y. Preparation of p-nitrophenyl β-L-arabinofuranoside as a substrate of β-L-arabinofuranosidase. Carbohydr. Res. 2013, 382, 95–100. 10.1016/j.carres.2013.10.005. 24239541

[ref27] Yamamura R.; Inoue K. Y.; Nishino K.; Yamasaki S. Intestinal and fecal pH in human health. Front. Microbiomes 2023, 2, 1192316 10.3389/frmbi.2023.1192316. PMC12993532 41853365

[ref28] Duncan S. H.; Louis P.; Thomson J. M.; Flint H. J. Role of pH in determining the species composition of the human colonic microbiota. Environ. Microbiol. 2009, 8, 2112–2122. 10.1111/j.1462-2920.2009.01931.x. 19397676

[ref29] Parhi P.; Song K. P.; Choo W. S. Growth and survival of *Bifidobacterium breve* and *Bifidobacterium longum* in various sugar systems with fructooligosaccharide supplementation. J. Food Sci. Technol. 2022, 59, 3775–3786. 10.1007/s13197-022-05361-z. 36193365 PMC9525548

[ref30] Gloster T. M.; Madsen R.; Davies G. J. Structural basis for cyclophellitol inhibition of a β-glucosidase. Org. Biomol. Chem. 2007, 5, 444–446. 10.1039/B616590G. 17252125

[ref31] Gloster T. M.; Davies G. J. Glycosidase inhibition: assessing mimicry of the transition state. Org. Biomol. Chem. 2010, 8, 305–320. 10.1039/B915870G. 20066263 PMC2822703

[ref32] Wu L.; Jiang J.; Jin Y.; Kallemeijn W. W.; Kuo C.-L.; Artola M.; Dai W.; van Elk C.; van Eijk M.; van der Marel G. A.; Codée J. D. C.; Florea B. I.; Aerts J. M. F. G.; Overkleeft H. S.; Davies G. J. Activity-based probes for functional interrogation of retaining β-glucuronidases. Nat. Chem. Biol. 2017, 13, 867–873. 10.1038/nchembio.2395. 28581485

[ref33] Willems L. I.; Beenakker T. J. M.; Murray B.; Scheij S.; Kallemeijn W. W.; Boot R. G.; Verhoek M.; Donker-Koopman W. E.; Ferraz‡ M. J.; van Rijssel E. R.; Florea B. I.; Codée J. D. C.; van der Marel G. A.; Aerts J. M. F. G.; Overkleeft H. S. Potent and selective activity-based probes for GH27 human retaining α-galactosidases. J. Am. Chem. Soc. 2014, 136, 11622–11625. 10.1021/ja507040n. 25105979

[ref34] Witte M. D.; Kallemeijn W. W.; Aten J.; Li K.-Y.; Strijland A.; Donker-Koopman W. E.; van den Nieuwendijk A. M. C. H.; Bleijlevens B.; Kramer G.; Florea B. I.; Hooibrink B.; Hollak C. E. M.; Ottenhoff R.; Boot R. G.; van der Marel G. A.; Overkleeft H. S.; Aerts J. M. F. G. Ultrasensitive in situ visualization of active glucocerebrosidase molecules. Nat. Chem. Biol. 2010, 6, 907–913. 10.1038/nchembio.466. 21079602

[ref35] D’Arcy A.; Bergfors T.; Cowan-Jacob S. W.; Marsh M. Mi-croseed matrix screening for optimization in protein crystallization: what have we learned?. Acta Crystallogr., Sect. F: Struct. Biol. Commun. 2014, 70, 1117–1126. 10.1107/S2053230X14015507. 25195878 PMC4157405

[ref36] Shaw Stewart P. D.; Kolek S. A.; Briggs R. A.; Chayen N. E.; Baldock P. F. M. Random microseeding: a theoretical and practical exploration of seed stability and seeding techniques for successful protein crystallization. Cryst. Growth Des. 2011, 11, 3432–3441. 10.1021/cg2001442.

[ref37] Shah A. K.; Liu Z.-J.; Stewart P. D.; Schubot F. D.; Rose J. P.; Newton M. G.; Wang B.-C. On increasing protein-crystallization throughput for X-ray diffraction studies. Acta Crystallogr., Sect. D: Struct. Biol. 2005, 61, 123–129. 10.1107/S0907444904027064. 15681862

[ref38] Winter G.; Lobley C. M. C.; Prince S. M. Decision making in xia2. Acta Crystallogr., Sect. D: Biol. Crystallogr. 2013, 69, 1260–1273. 10.1107/S0907444913015308. 23793152 PMC3689529

[ref39] Lebedev A. A.; Young P.; Isupov M. N.; Moroz O. V.; Vagin A. A.; Murshudov G. N. JLigand: a graphical tool for the CCP4 tem-plate-restraint library. Acta Crystallogr., Sect. D: Biol. Crystallogr. 2012, 68, 431–440. 10.1107/S090744491200251X. 22505263 PMC3322602

[ref40] Murshudov G. N.; Skubák P.; Lebedev A. A.; Pannu N. S.; Steiner R. A.; Nicholls R. A.; Winn M. D.; Long F.; Vagin A. A. REFMAC5 for the refinement of macromolecular crystal structures. Acta Crystallogr., Sect. D: Biol. Crystallogr. 2011, 67, 355–367. 10.1107/S0907444911001314. 21460454 PMC3069751

[ref41] Vagin A.; Teplyakov A. Molecular replacement with MOLREP. Acta Crystallogr., Sect. D: Biol. Crystallogr. 2010, 66, 22–25. 10.1107/S0907444909042589. 20057045

[ref42] Kabsch W. XDS. Acta Crystallogr. 2010, D66, 125–132. 10.1107/S0907444909047337. PMC2815665 20124692

[ref43] Gildea R. J.; Beilsten-Edmands J.; Axford D.; Horrell S.; Aller P.; Sandy J.; Sanchez-Weatherby J.; Owen C. D.; Lukacik P.; Strain-Damerell C.; Owen R. L.; Walsh M. A.; Winter G. xia2.multiplex: a multi-crystal data-analysis pipeline. Acta Crystallogr., Sect. D: Struct. Biol. 2022, 78, 752–769. 10.1107/S2059798322004399. 35647922 PMC9159281

[ref44] Winn M. D.; Ballard C. C.; Cowtan K. D.; Dodson E. J.; Emsley P.; Evans P. R.; Keegan R. M.; Krissinel E. B.; Leslie A. G. W.; McCoy A.; McNicholas S. J.; Murshudov G. N.; Pannu N. S.; Potterton E. A.; Powell H. R.; Read R. J.; Vagin A.; Wilson K. S. Overview of the CCP4 suite and current developments. Acta Crystallogr., Sect. D: Biol. Crystallogr. 2011, 67, 235–242. 10.1107/S0907444910045749. 21460441 PMC3069738

[ref45] Krissinel E.; Lebedev A. A.; Uski V.; Ballard C. B.; Kee-gan R. M.; Kovalevskiy O.; Nicholls R. A.; Pannu N. S.; Skubák P.; Berrisford J.; Fando M.; Lohkamp B.; Wojdyr M.; Simpkin A. J.; Thomas J. M. H.; Oliver C.; Vonrhein C.; Chojnowski G.; Basle A.; Purkiss A.; Isupov M. N.; McNicholas S.; Lowe E.; Triviño J.; Cowtan K.; Agirre J.; Rigden D. J.; Uson I.; Lamzin V.; Tews I.; Bricogne G.; Leslie A. G. W.; Brown D. G. CCP4 Cloud for structure determination and project management in macromolecular crystallography. Acta Crystallogr., Sect. D: Struct. Biol. 2022, 78, 1079–1089. 10.1107/S2059798322007987. 36048148 PMC9435598

[ref46] Emsley P.; Cowtan K. Coot: model-building tools for mo-lecular graphics. Acta Crystallogr., Sect. D: Biol. Crystallogr. 2004, 60, 2126–2132. 10.1107/S0907444904019158. 15572765

[ref47] Long F.; Nicholls R. A.; Emsley P.; Graǽulis S.; Merkys A.; Vaitkus A.; Murshudov G. N. AceDRG: a stereochemical description generator for ligands. Acta Crystallogr., Sect. D: Struct. Biol. 2017, 73, 112–122. 10.1107/S2059798317000067. 28177307 PMC5297914

[ref48] Potterton L.; Agirre J.; Ballard C.; Cowtan K.; Dodson E.; Evans P. R.; Jenkins H. T.; Keegan R.; Krissinel E.; Stevenson K.; Lebedev A.; McNicholas S. J.; Nicholls R. A.; Noble M.; Pannu N. S.; Roth C.; Shel-drick G.; Skubak P.; Turkenburg J.; Uski V.; von Delft F.; Waterman D.; Wilson K.; Winn M.; Wojdyr M. CCP4i2: the new graphical user interface to the CCP4 program suite. Acta Crystallogr., Sect. D: Struct. Biol. 2018, 74, 68–84. 10.1107/S2059798317016035. 29533233 PMC5947771

[ref49] Car R.; Parrinello M. Unified approach for molecular dy-namics and density-functional theory. Phys. Rev. Lett. 1985, 55, 2471–2474. 10.1103/PhysRevLett.55.2471. 10032153

[ref50] Troullier N.; Martins J. L. Efficient pseudopotentials for plane-wave calculations. II. Operators for fast iterative diagonalization. Phys. Rev. B 1991, 43, 8861–8869. 10.1103/PhysRevB.43.8861. 9996554

[ref51] Perdew J. P.; Burke K.; Ernzerhof M. Generalized Gradient Approximation Made Simple. Phys. Rev. Lett. 1996, 77, 3865–3868. 10.1103/PhysRevLett.77.3865. 10062328

[ref52] Laio A.; Parrinello M. Escaping free-energy minima. Proc. Natl. Acad. Sci. U.S.A. 2002, 99, 12562–12566. 10.1073/pnas.202427399. 12271136 PMC130499

[ref53] Tribello G. A.; Bonomi M.; Branduardi D.; Camilloni C.; Bussi G. PLUMED 2: New feathers for an old bird. Comput. Phys. Commun. 2014, 185, 604–613. 10.1016/j.cpc.2013.09.018.

[ref54] Huang M.; Giese T. J.; Lee T. S.; York D. M. Improvement of DNA and RNA Sugar Pucker Profiles from Semiempirical Quantum Methods. J. Chem. Theory Comput. 2014, 10, 1538–1545. 10.1021/ct401013s. 24803866 PMC3985690

[ref55] Tiwary P.; Parrinello M. A Time-Independent Free Energy Estimator for Metadynamics. J. Phys. Chem. B 2015, 119, 736–742. 10.1021/jp504920s. 25046020

