# Supplementary material for: β-l-Arabinofurano-cyclitol Aziridines Are Covalent Broad-Spectrum Inhibitors and Activity-Based Probes for Retaining β-l-Arabinofuranosidases
Source: ACS Chem Biol. 2023 Dec 5;18(12):2564–73. doi: 10.1021/acschembio.3c00558 (PMC10728902; doi:10.1021/acschembio.3c00558)
Supplement: Supplementary file 1 — cb3c00558_si_001.pdf [file cb3c00558_si_001.pdf]

# Supporting Information

## β-L-*Arabino*furano-cyclitol Aziridines are Covalent Broad-spectrum Inhibitors and Activity-based Probes for Retaining β- L-arabinofuranosidases

Valentina Borlandelli,[1] Wendy Offen,[2] Olga Moroz,[2] Alba Nin-Hill,[3] Nicholas McGregor,[2] Lars Binkhorst,[1] Akihiro Ishiwata,[4] Zachary Armstrong,[1] Marta Artola,[1] Carme Rovira,[3] Gideon J. Davies,[2] Herman S. Overkleeft[1]

[1]Bio-organic Synthesis, Leiden Institute of Chemistry (LIC), Leiden University, Gorlaeus Laboratories, Einsteinweg 55, 2333 CC Leiden, The Netherlands.

[2]Department of Chemistry, York Structural Biology Laboratory, University of York, Heslington, York YO10 5DD, United Kingdom.

[3]Departament de Química Inorgànica i Orgànica (Secció de Química Orgànica) and Institut de Química Teòrica i Computacional (IQTCUB), Universitat de Barcelona, Martí i Franquès 1, 08028 Barcelona, Spain.

[4]RIKEN Cluster for Pioneering Research, 2-1 Hirosawa, Wako, Saitama, 351-0198 Japan.

Email of corresponding authors: h.s.overkleeft@lic.leidenuniv.nl; gideon.davies@york.ac.uk

**TABLE OF CONTENTS**


1. **Supporting Figures** **S3**
2. **Materials and Methods** **S5**
   - **2.1 Chemical Synthesis** **S5**
     - **2.1.1 General Experimental Details** **S5**
     - **2.1.2 Experimental Procedures and Characterisation Data of Compounds** **S6**
   - **2.2 Crystallographic Data Collection and Refinement Statistics** **S14**
3. **NMR Spectra** **S17**
4. **References** **S31**

## 1. Supporting Figures

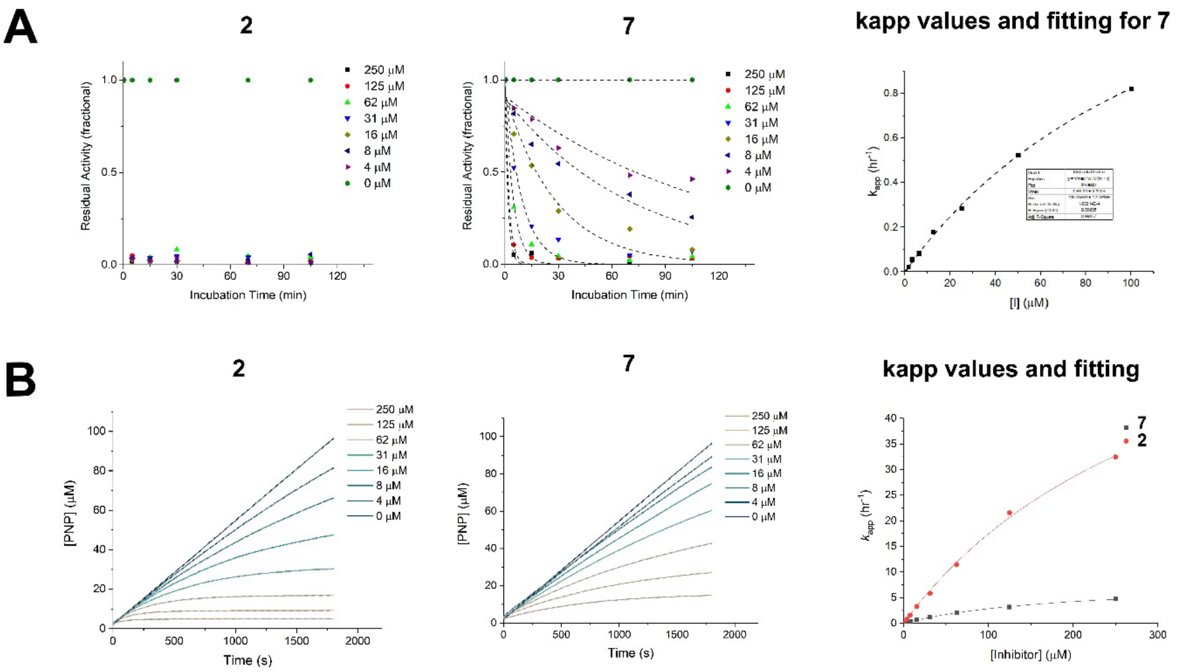


**Figure S1.** Inhibition kinetics for compounds **2**, **4** and **7** against HypBA1 and *Bt*GH146. (A) Inhibition of HypBA1. Plots of residual enzyme activity *vs* incubation time at different concentration of inhibitors **2** and **7**. Parameters for irreversible inhibition by **7** is modelled by plotting measured inactivation rate constants ($k_{app}$) *vs* [Inhibitor]. (B) Inhibition of *Bt*GH146. Plots of measured residual absorbance at 405 nm (A405) corresponding to *p*-nitrophenol (PNP) concentration [PNP] *vs* incubation time at different concentration of inhibitors **2**, **4** and **7**. Parameters for irreversible inhibition by **2**, **4** and **7** are modelled by plotting measured inactivation rate constants ($k_{app}$) *vs* [Inhibitor].

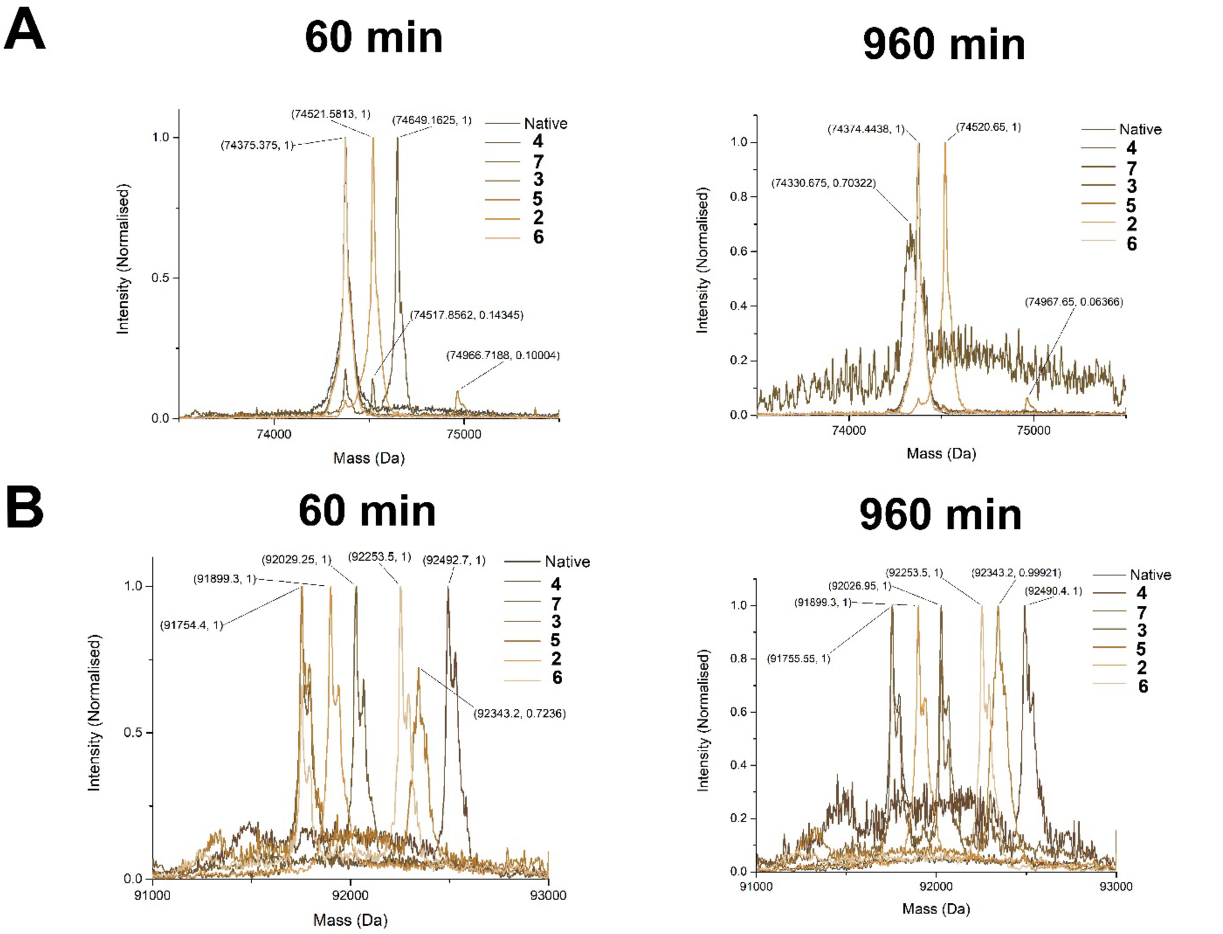


**Figure S2.** Intact mass spectrometry (MS) of tested compounds **2** – **7** with *Bt*GH146 and HypBA1. (A) Intact MS of HypBA1 following incubation with with 0.1 mM compound in pH 4.5 Na-acetate buffer at 22 °C for different lengths of time (60 or 960 minutes). Expected mass (native): 74376 Da, with **2** : 74521 Da, with **3** : 74649 Da, with **4** : 75113 Da, with **5** : 74988 Da, with **6** : 74875 Da, with **7** : 74648 Da. (B) Intact MS of *Bt*GH146 following incubation with with 0.1 mM compound in pH 7.5 HEPES buffer at 37 °C for different lengths of time (60 or 960 minutes). Expected mass (native): 91512 Da, with **2** : 91657 Da, with **3** : 91785 Da, with **4** : 92250 Da, with **5** : 92123 Da, with **6** : 92011 Da, with **7** : 91784 Da.

# 2. Materials and Methods

## 2.1 Chemical Synthesis

### 2.1.1. General Experimental Details

Chemicals were purchased from Sigma Aldrich, unless stated otherwise. Tetrahydrofuran (THF), dichloromethane (DCM), *N*,*N*-dimethylformamide (DMF) and toluene were stored over molecular sieves before use. Traces of water from reagents were removed by co-evaporation with toluene in reactions that required anhydrous conditions. All reactions were performed under an argon atmosphere unless stated otherwise. TLC analysis was conducted using Merck aluminum sheets (Silica gel 60 F254) with detection by UV absorption (254 nm), by spraying with a solution of $(NH_4)_6Mo_7O_{24}\cdot 4H_2O$ (25 g/L) and $(NH_4)_4Ce(SO_4)_4\cdot 2H_2O$ (10 g/L) in 10% sulfuric acid or a solution of $KMnO_4$ (20 g/L) and $K_2CO_3$ (10 g/L) in water, followed by charring at ~150 ˚C. Column chromatography was performed using Screening Device b.v. silica gel (particle size of 40 – 63 μm, pore diameter of 60 Å) with the indicated eluents. For reversed-phase HPLC purifications, an Agilent Technologies 1200 series instrument equipped with a semi-preparative column (Gemini C18, 250 x 10 mm, 5 μm particle size, Phenomenex) or with an HILIC column was used. LC/MS analysis was performed on a Surveyor HPLC system (Thermo Finnigan) equipped with a C18 column (Gemini, 4.6 mm x 50 mm, 5 μm particle size, Phenomenex), coupled to a LCQ Advantage Max (Thermo Finnigan) ion-trap spectrometer (ESI+). The applied buffers were: (A) $H_2O$, (B) $CH_3CN$ and (C) aqueous 20 mM or 50 mM $NH_4HCO_3$;. $^1H$ NMR and $^{13}C$ NMR spectra were recorded on a Brüker AV-400 (400 and 101 MHz respectively, Brüker AV-500 (500 MHz and 126 MHz) or a Brüker DMX-600 (600 and 151 MHz respectively) spectrometer in the given solvent. Chemical shifts are given in ppm (*δ*) relative to the residual solvent peak or tetramethylsilane (0 ppm) as internal standard. Coupling constants are given in Hz. $^{13}C$ NMR spectra are Attached Proton Test (APT) spectra with phase inverted (180 degrees). High-resolution mass spectrometry (HRMS) analysis was performed with a LTQ Orbitrap mass spectrometer (Thermo Finnigan), equipped with an electronspray ion source in positive mode (source voltage 3.5 kV, sheath gas flow 10 mL/min, capillary temperature 250 °C) with resolution R = 60000 at m/z 400 (mass range m/z = 150 – 2000) and dioctyl phthalate (m/z = 391.28428) as a “lock mass”. The high-resolution mass spectrometer was calibrated prior to measurements with a calibration mixture (Thermo Finnigan).

### 2.1.2. Experimental Procedures and Characterisation Data of Compounds

### General Procedure A | Birch reaction

Ammonia (5–10 mL) was condensed in an oven-dried flask cooled to –60 °C. Freshly-cut $Na_{(s)}$ wires (22 equiv.) were added to the liquid ammonia and stirred with a glass-coated stirring bar at –60 °C until complete dissolution (20 minutes). Next, a bisbenzylated compound (**13**, **14** or **17**, 20 – 50 µmol, 1 equiv.) was dissolved in dry THF (1 mL) and $^t$BuOH (28 equiv.). The resulting solution was added drop-wise along the cold walls of the flask containing liquid ammonia at -60 °C. Upon addition, the solution remained dark blue and was stirred for additional 45 min at –60 °C. Next, the reaction was quenched (with 1 mL milli-Q $H_2O$ for aziridine-based compounds **13** and **14**; with 28 equiv. of 3.1 M $NH_4Cl$ aqueous solution (180 – 452 µL) for epoxide-based compound **17**). The reaction mixture was allowed to attain gradually room temperature over 2 h. Crude was co-evaporated thrice with milli-Q water, and purified as described for each distinct product.

### General Procedure B | Amide coupling of reporter tag

To a solution of tag-COOH (1 equiv.) in dry DMF (0.154 – 0.176 M), PFP-TFA (1.5 equiv.) and DIPEA (3.5 equiv.) were added under stirring. After 2 h, the reaction was quenched with Milli-Q $H_2O$ (2.1 equiv.), and the mixture was added at room temperature to a solution of the desired β-L-*arabino*furanosyl amine **3** or **7** (0.77 equiv.), either dissolved in dry DMF (250 µL) or used as dry solid. Additional DIPEA (2.5 equiv.) was added to the reaction mixture. Upon full conversion (as monitored by LC-MS), the solution was concentrated to dryness and the crude purified by reverse-phase HPLC or by silica gel chromatography as described. Volatiles were removed by lyophilization.

### *O*-(8'-azido-octyl)-2,3-bis-benzylated-β-L-*arabino*furanosyl cyclophellitol 16

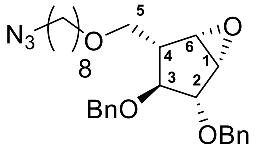


Epoxide **15**[1] (120 mg, 0.369 mmol) was dissolved in dry THF (0.700 mL, 0.21 M) under $N_2$ (g) atmosphere, and cooled to 0 °C. NaH (60% wt, 7.73 mg, 0.193 mmol) was added and the solution was stirred for 15 min at 0 °C. Next, the solution was cooled to –20 °C, and freshly prepared azido-octyltriflate[2] solution (0.383 M in THF, 0.500 mL, 0.193 mmol) was added dropwise along the cold flask walls. The solution was gradually warmed up to rt. After 5 h, TLC showed full consumption of the starting material. The reaction was quenched on ice with water, diluted with EtOAc (20 mL) and washed with water, and brine. Organic layer was dried over $Na_2SO_4$, filtered and volatiles were removed under reduced pressure. Purification by silica gel column chromatography (pentane/EtOAc 20:1→7:1) afforded titled product as an oil (149 mg, 0.310 mmol, 84%). $^1$H NMR (500 MHz, $CDCl_3$): $\delta$ = 7.44 – 7.23 (m, 10H, CH Ar), 4.77 (d, $J$ = 11.9

Hz, 1H, C*H*HPh), 4.71 – 4.62 (m, 2H, $CH_2Ph$), 4.54 (d, *J* = 11.6 Hz, 1H, CH*H*Ph), 4.09 (dd, *J* = 5.3, 1.4 Hz, 1H, H-2), 3.61 – 3.52 (m, 4H, H-1/H-6/$CH_2$-5), 3.45 (td, *J* = 6.6, 2.4 Hz, 2H, $OCH_2$linker), 3.38 (dd, *J* = 7.1, 5.3 Hz, 1H, H-3), 3.24 (t, *J* = 7.0 Hz, 2H, $CH_2N_3$), 2.29 (dddd, *J* = 9.0, 6.9, 5.4, 1.5 Hz, 1H, H-4), 1.58 (td, *J* = 7.5, 5.4 Hz, 5H, 5 CH*H*linker), 1.42 – 1.21 (m, 11H, 11 CH*H*linker) ppm. $^{13}$C NMR (126 MHz, $CDCl_3$): $\delta$ = 138.5, 138.2 (2$C_q$), 128.6, 128.5, 128.0, 127.9, 127.8 (6CH Ar), 85.8 (CH, C-2), 81.6 (CH, C-3), 72.6 ($OCH_2$linker), 71.8, 71.5 (2$CH_2$Ph), 69.6 ($CH_2$, C-5), 55.3 (CH, epoxide), 54.5 (CH, epoxide), 51.6 ($CH_2N_3$), 45.2 (CH, C-4), 29.8, 29.4, 29.2, 28.9, 26.8, 26.2 (6$CH_2$, linker) ppm. HR-MS (ESI): *m/z* calcd for $C_{28}H_{41}N_4O_4^+$: 497.31223 [*M*+$NH_4$]$^+$, found 497.31214; *m/z* calcd for $C_{28}H_{37}N_3O_4Na^+$ 502.26763 [*M*+Na]$^+$, found 502.26752.

***O*-(8'-amino-octyl)-2,3-bis-benzylated-β-L-*arabino*furanosyl cyclophellitol 17**

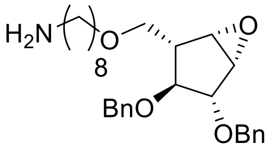


Azide **16** (149 mg, 0.311 mmol) was dissolved in dry acetonitrile (6.22 mL, 0.05 M) and transferred into a microwave tube. To this solution, water (56 µL, 3.11 mmol) and polymer-bound $PPh_3$ (163 mg, 0.622 mmol) were added at rt. The reaction mixture was heated to 70 °C, and stirred for 5 h. After 5 h, TLC showed full conversion; hence the solution was cooled to room temperature and filtered over a sintered glass filter. The filter was washed with acetonitrile (3x 13 mL), volatiles were removed under reduced pressure and the crude was used in the next step without further purification (133 mg, 0.292 mmol, 94%). $^1$H NMR (500 MHz, $CDCl_3$): $\delta$ = 7.46 – 7.22 (m, 10H, CH Ar), 4.77 (d, *J* = 11.9 Hz, 1H, ½ $CH_2Ph$), 4.70 – 4.62 (m, 2H, $CH_2Ph$), 4.54 (d, *J* = 11.6 Hz, 1H, ½ $CH_2Ph$), 4.09 (dd, *J* = 5.3, 1.4 Hz, 1H, H-2), 3.62 – 3.52 (m, 4H, H-1/H-6/$CH_2$-5), 3.44 (td, *J* = 6.7, 2.3 Hz, 2H, $OCH_2$linker), 3.38 (dd, *J* = 7.1, 5.3 Hz, 1H, H-3), 2.72 – 2.60 (m, 2H, C*H*$_2$$NH_2$), 2.29 (dddd, *J* = 9.0, 7.0, 5.4, 1.5 Hz, 1H, H-4), 1.82 (s, 2H, $NH_2$), 1.63 – 1.51 (m, 2H, $CH_2$linker), 1.49 – 1.37 (m, 2H, 2CH*H*linker), 1.36 – 1.21 (m, 10H, 8CH*H*linker, grease) ppm. $^{13}$C NMR (126 MHz, $CDCl_3$): $\delta$ = 138.4, 138.1 (2$C_q$), 128.7, 128.6, 128.4, 127.9, 127.7 (6CH Ar), 85.8 (CH, C-2), 81.6 (CH, C-3), 72.6 ($OCH_2$linker), 71.8, 71.5 (2$CH_2$Ar), 69.5 ($CH_2$, C-5), 55.3 (CH, epoxide), 54.5 (CH, epoxide), 45.2 (CH, C-4), 42.2 ($CH_2NH_2$), 33.6, 29.7, 29.5, 26.9, 26.8, 26.2 (6$CH_2$, linker) ppm. HR-MS (ESI): *m/z* calcd for $C_{28}H_{40}NO_4^+$: 454.29519 [*M*+H]$^+$, found 454.29501.

***O*-(8'-amino-octyl)-β-L-*arabino*furanosyl cyclophellitol 3**

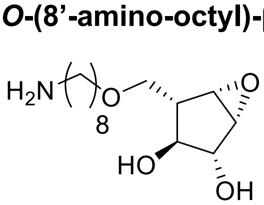


Ammonia was condensed in an oven-dried flask at –60 °C, and sodium (189 mg, 8.21 mmol) was freshly cut and added to the liquid ammonia solution at –60 °C. The solution was stirred at this temperature for 40 min. Amine **17** (133 mg, 0.292 mmol) was co-evaporated thrice with distilled toluene and then dissolved in dry THF (5 ml) and $^t$BuOH (1 mL, 10.46 mmol). The resulting solution was added drop-wise along the cold walls of the sodium/ammonia solution

at –60 °C, and the reaction was stirred at this temperature. After 45 min, the reaction was quenched with an aqueous solution of 3.11 M $NH_4Cl$ (3 ml, 9.35 mmol) and it was allowed to warm up to rt. Crude was co-evaporated thrice with Milli-Q water and desalted by size-exclusion chromatography over Bio-Gel P-2 resin eluting with 1% AcOH in water. The desalted fractions were purified by reverse-phase HPLC chromatography equipped with HILIC column (eluent A: 50 mM $NH_4HCO_3$ in water; eluent B: $CH_3CN$). The isolated product was lyophilised upon NMR analysis, to yield titled compound as pale yellow solid (79.5 mg, 0.245 mmol, 84%). $^1$H NMR (500 MHz, MeOD): $\delta$ = 3.95 (dd, $J$ = 6.0, 1.5 Hz, 1H, H-2), 3.64 (dd, $J$ = 9.3, 4.6 Hz, 1H, C*H*H-5), 3.56 (t, $J$ = 9.4 Hz, 1H, CH*H*-5), 3.52 (t, $J$ = 6.5 Hz, 1H, OC*H*$H_2$linker), 3.51 – 3.47 (m, 2H, H-epoxide/ ½ OCH*H*linker), 3.46 (dd, $J$ = 3.2, 1.5 Hz, 1H, H-epoxide), 3.19 (dd, $J$ = 7.6, 6.0 Hz, 1H, H-3), 2.89 (t, $J$ = 7.7 Hz, 2H, C*H*$_2$$NH_2$), 2.06 (dddd, $J$ = 9.5, 7.6, 4.6, 1.3 Hz, 1H, H-4), 1.90 (s, 3H, OAc), 1.71 – 1.55 (m, 4H, 2 $CH_2$linker), 1.49 – 1.35 (m, 8H, 4 $CH_2$linker), 1.22 (s, 2H) ppm. $^{13}$C NMR (126 MHz, MeOD): δ 80.2 (CH), 76.1 (CH), 72.3 ($CH_2$), 70.5 ($CH_2$), 57.8 (CH, epoxide), 55.1 (CH, epoxide), 47.4 (CH, C-4), 40.8 ($CH_2NH_2$), 31.1 (CH), 30.6, 30.3, 30.2, 28.9, 27.4, 27.1 (6 $CH_2$linker) ppm. HR-MS (ESI): *m/z* calcd for $C_{14}H_{28}NO_4^+$: 274.20128 [*M*+H]$^+$, found 274.20130.

**β-L-*arabino*furanosyl-cyclophellitol Cy5-tagged ABP 4**

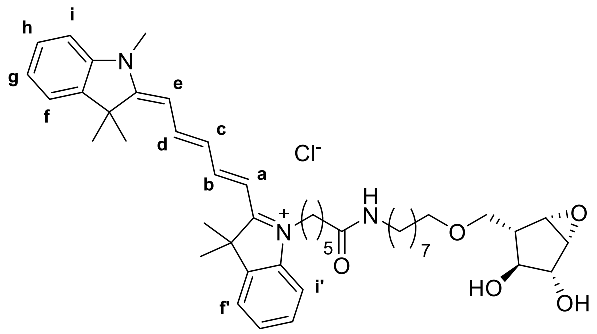


Titled product was obtained as a blue powder (2.2 mg, 2.9 µmol, 7.4% yield) starting from amine **3** (10 mg, 39 µmol) according to general procedure B, upon purification by reverse-phase HPLC chromatography (A: 50 mM $NH_4OAc$ in $H_2O$, B: $CH_3CN$). $^1$H NMR (850 MHz, MeOD): $\delta$ = 8.29 – 8.20 (m, 2H, H-d, H-b), 7.51 – 7.48 (m, 2H, H-i, H-i'), 7.41 (qd, $J$ = 7.4, 1.2 Hz, 2H, H-f, H-f'), 7.34 – 7.23 (m, 4H), 6.63 (t, $J$ = 12.4 Hz, 1H), 6.28 (dd, $J$ = 13.7, 10.1 Hz, 2H), 4.11 (t, $J$ = 7.5 Hz, 1H, $CH_2N^+$), 3.94 (dd, $J$ = 6.0, 1.5 Hz, 1H, H-2), 3.64 – 3.61 (m, 4H, $CH_3N$, C*H*H), 3.55 – 3.52 (m, 1H, CH*H*), 3.52 – 3.43 (m, 4H, $CH_2$, H-1, H-6), 3.18 (dd, $J$ = 7.7, 6.0 Hz, 1H, H-3), 3.12 (t, $J$ = 7.2 Hz, 2H, C*H*$_2$NHCO), 2.20 (t, $J$ = 7.3 Hz, 2H, $CH_2$C=O), 2.09 – 2.01 (m, 1H, H-4), 1.83 (p, $J$ = 7.8 Hz, 2H, C*H*$_2$$CH_2N^+$), 1.73 (s, 6H, 2$CH_3$), 1.69 (p, $J$ = 7.4 Hz, 2H, C*H*$_2$$CH_2$C=O), 1.63 – 1.54 (m, 2H, 2CH*H*linker), 1.49 – 1.42 (m, 4H, 4CH*H* linker), 1.39 – 1.27 (m, 10H, 10CH*H* linker), 1.22 (s, 3H, $CH_3$), 0.93 – 0.85 (m, 2H, 2CH*H* linker) ppm. $^{13}$C NMR (214 MHz, MeOD): $\delta$ = 175.7, 175.4, 174.7 (3$C_q$), 155.53 (CH Ar), 144.3, 143.6, 142.6, 142.5 (4$C_q$), 129.8, 126.6, 126.3, 126.2, 123.4, 123.3, 112.1, 111.8, 104.4, 104.3 (11CH Ar), 80.2 (CH, C-2), 76.1 (CH, C-3), 72.4 ($CH_2$), 70.5 ($CH_2$) 57.8 (CH, C-1/C-6), 55.1 (CH, C-1/C-6), 47.3 (CH, C-4), 44.8 ($CH_2N^+$), 40.4 ($CH_2$NH), 36.7 (*C*H$_2$C=O), 31.1 ($CH_3N$), 30.7, 30.5, 30.4, 28.2, 28.0 (6$CH_2$, linker), 27.9,

27.8 (2$CH_3$), 27.4, 27.2, 26.6 (3$CH_2$, linker) ppm. HR-MS (ESI): *m/z* calcd for $C_{46}H_{64}N_3O_5^+$: 738.48405 [*M*]$^+$, found 738.48412.

**β-L-*arabino*furanosyl-cyclophellitol greenBODIPY-tagged ABP 5**

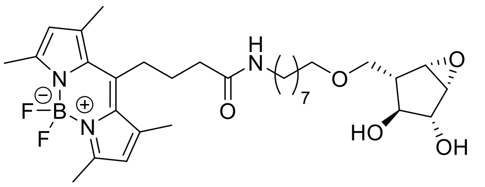

Titled product was obtained as an orange-red powder (14.1 mg, 23.9 µmol, 41% yield) according to general procedure B starting from amine **3** (30 mg, 59 µmol) with purification by silica gel chromatography (DCM/MeOH 99:1→95:5). $^1$H NMR (500 MHz, MeOD): $\delta$ = 6.14 (s, 2H, 2H Ar), 3.93 (d, *J* = 6.0 Hz, 1H, H-2), 3.62 (dd, *J* = 9.4, 4.7 Hz, 1H, C*H*H-5), 3.57 – 3.50 (m, 1H, CH*H*-5), 3.51 – 3.41 (m, 4H, $CH_2$OC(5), H-1, H-6), 3.18 (q, *J* = 6.9, 6.3 Hz, 3H, C*H*$_2$NH, H-3), 3.07 – 2.97 (m, 2H, C*H*$_2$$CH_2$$CH_2$C=O), 2.47 – 2.43 (m, 12H, 4$CH_3$), 2.38 (t, *J* = 7.1 Hz, 2H, $CH_2$C=O), 2.05 (td, *J* = 8.8, 4.5 Hz, 1H, H-4), 1.99 – 1.87 (m, 2H, C*H*$_2$$CH_2$C=O), 1.64 – 1.54 (m, 2H, 2CH*H* linker), 1.51 (t, *J* = 6.9 Hz, 2H, 2CH*H* linker), 1.43 – 1.32 (m, 10H, 8CH*H* linker) ppm. $^{13}$C NMR (126 MHz, MeOD): $\delta$ = 174.8, 155.2, 147.2, 142.4, 132.6 (5$C_q$), 122.7 (2CH Ar), 80.2 (CH, C-2), 76.1 (CH, C-3), 72.4 ($CH_2$O linker), 70.5 ($CH_2$, C-5), 57.8 (CH, C-1/C-6, epoxide), 55.1 (CH, C-1/C-6, epoxide), 47.4 (CH, C-4), 40.5 ($CH_2$NH), 37.1 (*C*H$_2$C=O), 30.7, 30.5, 30.4, 29.3, 28.6, 28.0, 27.2 (7$CH_2$ linker), 16.6 (4$CH_3$), 14.4 ppm. HR-MS (ESI): *m/z* calcd for $C_{31}H_{47}BF_2N_4O_4Na^+$: 612.33963 [*M*+Na]$^+$, found 612.33922.

**β-L-*arabino*furanosyl-cyclophellitol biotin-tagged ABP 6**

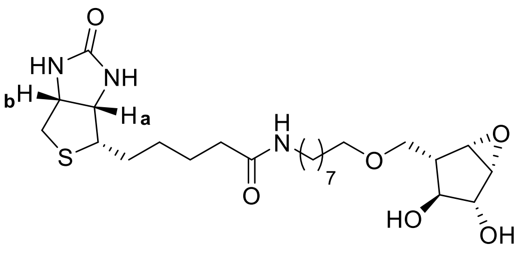

Amine **3** (9.89 mg, 36.3 µmol) was reacted according to general protocol B ensued by reverse-phase HPLC purification (A: 20 mM $NH_4OAc$ in $H_2O$, B: $CH_3CN$), to afford the titled product as a white powder (4.27 mg, 8.5 µmol, 23% yield). After NMR analysis, the desired product was lyophilised thrice to remove volatiles. $^1$H NMR (500 MHz, MeOD): $\delta$ = 4.49 (ddd, *J* = 7.9, 5.0, 0.9 Hz, 1H, H-b), 4.30 (dd, *J* = 7.9, 4.5 Hz, 1H, H-a), 3.94 (dd, *J* = 6.0, 1.5 Hz, 1H, H-2), 3.64 (dd, *J* = 9.3, 4.7 Hz, 1H, C*H*H-5), 3.59 – 3.44 (m, 5H, CH*H*-5, $CH_2$O linker, H-1, H-6), 3.24 – 3.10 (m, 4H, CHS, H-3, C*H*$_2$NHC=O), 2.93 (dd, *J* = 12.8, 5.0 Hz, 1H, C*H*HS), 2.71 (d, *J* = 12.7 Hz, 1H, CH*H*S), 2.24 – 2.15 (m, 2H, $CH_2$C=O), 2.06 (dddd, *J* = 9.7, 7.6, 4.6, 1.4 Hz, 1H, H-4), 1.79 – 1.55 (m, 6H, 6CH*H* linker), 1.53 – 1.31 (m, 12H, 12CH*H* linker) ppm. $^{13}$C NMR (126 MHz, MeOD): $\delta$ = 176.0, 166.1 (2$C_q$), 80.2 (CH, C-2), 76.1 (CH, C-3), 72.4 ($CH_2$O, linker), 70.5 ($CH_2$, C-5), 63.4 (CH, C-a), 61.6 (CH, C-b), 57.8 (CH, C-1), 57.0 (CHS), 55.1 (CH, C-6), 47.4 (CH, C-4), 41.1 ($CH_2$S), 40.4 ($CH_2$NH), 36.8 (*C*H$_2$C=O), 30.7, 30.5, 30.4, 30.4, 29.8, 29.5, 27.9, 27.2, 27.0 (9$CH_2$, linker) ppm. HR-MS (ESI): *m/z* calcd for $C_{24}H_{42}N_3O_6S^+$: 500.27888 [*M*+H]$^+$, found 500.27884.

**(4aS,5S,6R,7R,7aS)-5,6-bis(benzyloxy)-7-iodo-2-(trichloromethyl)-4,4a,5,6,7,7a-hexahydrocyclopenta[d][1,3]oxazine 12**

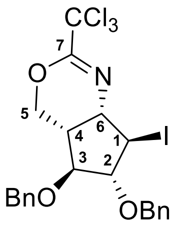

To a solution of cyclopentene **11** (2.73 g, 5.52 mmol) in $CHCl_3$ (138 mL) at 0 °C, NIS (310 g, 13.8 mmol) were added portion-wise under stirring. The solution was allowed to attain gradually rt. After 4 h, full conversion was observed and the solution was quenched with sat. aq. $NaHCO_3$ solution (70 mL), washed thrice with $CHCl_3$ (280 mL), dried over $Na_2SO_4$, filtered and concentrated to dryness. Crude was co-evaporated thrice with distilled toluene, and run through a short silica plug, yielding the desired product as an oil (3.2 g, 5.52 mmol, quant. yield). $^1$H NMR (400 MHz, $CDCl_3$): $\delta$ = 7.41 – 7.27 (m, 10H, 10CH Ar), 4.79 (d, $J$ = 11.6 Hz, 1H, 1CH*H*Ph), 4.67 – 4.48 (m, 3H, 3CH*H*Ph), 4.46 (ddd, $J$ = 4.6, 3.6, 1.1 Hz, 1H, H-1), 4.34 – 4.25 (m, 2H, $CH_2$-5), 4.22 – 4.17 (m, 2H, H-6, H-2), 3.79 (dd, $J$ = 8.4, 4.5 Hz, 1H, H-3), 2.81 – 2.74 (m, 1H, H-4) ppm. $^{13}$C NMR (101 MHz, $CDCl_3$): $\delta$ = 152.9 (C-7), 137.5, 129.1, 127.5, 127.1 (10CH Ar), 92.6 (CH, C-1), 83.6 (CH, C-3), 72.8, 72.3 (2$CH_2$Ph), 65.6 ($CH_2$, C-5), 64.1 (CH, C-2), 38.9 (CH, C-4), 26.8 (CH, C-6) ppm. HR-MS (ESI): *m/z* calcd for $C_{22}H_{22}Cl_3INO_3^+$: 579.97045 [*M*+H]$^+$, found 579.97026.

**2,3-bis-benzylated-β-L-*arabino*furanosyl-cyclitol aziridine 13**

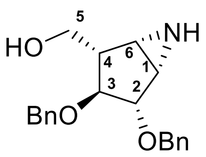

Cyclic trichloroacetimidate **12** (3.2 g, 5.52 mmol) was dissolved in a solution of MeOH/DCM (1:1 v/v, 110 mL) and the solution was cooled to 0 °C. A solution of HCl (1.25 M in MeOH, 39.7 mL, 49.7 mmol) was added dropwise at 0 °C. Upon addition, the solution was allowed to slowly warm up to rt and was stirred over three days. Upon full conversion, the reaction was quenched with $H_2O$ (0.5 mL). Afterwards, Amberlite IRA-67 was added under vigorous stirring until neutral pH. After 22 h full conversion was observed, and the reaction mixture was filtered over a glass filter and concentrated to dryness. Purification by silica gel chromatography (DCM/MeOH 99:1 + 0.05% TEA→98:2 + 0.05% TEA) afforded the titled product as a pale yellow oil (1.13 g, 3.47 mmol, 63% over 2 steps). $^1$H NMR (500 MHz, $CDCl_3$): $\delta$ = 7.47 – 7.23 (m, 10H, 10CH Ar), 4.76 (d, $J$ = 11.9 Hz, 1H, CH*H*Ph), 4.66 (q, $J$ = 23.5, 11.7 Hz, 2H, 2CH*H*Ph), 4.54 (d, $J$ = 11.5 Hz, 1H, CH*H*Ph), 4.12 (dd, $J$ = 5.5, 2.6 Hz, 1H, H-2), 3.90 (dd, $J$ = 10.7, 4.2 Hz, 1H, C*H*HOH), 3.79 (dd, $J$ = 10.7, 6.0 Hz, 1H, CH*H*OH), 3.64 (dd, $J$ = 7.5, 5.4 Hz, 1H, H-3), 2.80 – 2.54 (m, 2H, H-1, H-6), 2.24 – 2.16 (m, 1H, H-4) ppm. $^{13}$C NMR (126 MHz, $CDCl_3$): $\delta$ = 138.5, 138.4 (2$C_q$), 128.5, 128.0, 127.9, 127.8, 127.7 (6CH Ar), 85.9 (CH, C-2), 81.3 (CH, C-3), 72.6, 71.7 (2 $CH_2$Ph), 62.7 ($CH_2$, C-5), 46.0 (CH, C-4), 34.8 (CH, C-1/C-6), 33.4 (CH, C-1/C-6) ppm. HR-MS (ESI): *m/z* calcd for $C_{20}H_{24}NO_3^+$: 326.17507 [*M*+H]$^+$, found 326.17481.

**β-L-*arabino*furanosyl-cyclitol aziridine 2**

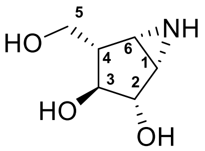


Aziridine **12** (31 mg, 95 μmol) was reacted according to general procedure A, yielding titled product (3.2 mg, 22 μmol, 23% yield) after HPLC purification with HILIC column (eluent A: 10 mM $NH_4OAc$ in $H_2O$; eluent B: $CH_3CN$). $^{1}H$ NMR (500 MHz, $D_2O$): $\delta$ = 3.71 (dd, $J$ = 6.4, 2.8 Hz, 1H, H-2), 3.43 (dd, $J$ = 11.2, 4.3 Hz, 1H, C*H*H-6), 3.29 (t, $J$ = 10.2 Hz, 1H, CH*H*-6), 2.79 (dd, $J$ = 8.0, 6.4 Hz, 1H, H-3), 2.37 – 2.28 (m, 2H, H-1, H-6), 1.73 – 1.62 (m, 1H, H-4) ppm. $^{13}C$ NMR (126 MHz, $D_2O$): $\delta$ = 78.8 (CH, C-2), 75.2 (CH, C-3), 61.2 ($CH_2OH$), 47.1 (CH, C-4), 36.4, 32.9 (2 CH, C-1, C-6) ppm. HR-MS (ESI): *m/z* calcd for $C_6H_{12}NO_3^+$: 146.08117 $[M+H]^+$, found 146.08121.

***N*-(8'-azido-octyl)-2,3-bis-benzylated-β-L-*arabino*furanosyl-cyclitol aziridine 14**

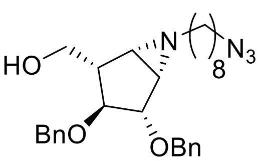


Aziridine **13** (673 mg, 2.13 mmol) was co-evaporated thrice at room temperature with dry distilled toluene. The compound was dissolved in dry DCM (20 mL, 0. M) and DIPEA (531 μL, 2.98 mmol) was added at room temperature. The solution was cooled to –20 °C. A solution of freshly prepared azido-octyltriflate[2] (0.49 M in DCM, 2.98 mmol) was added along the cold walls of the flask. The reaction was allowed to reach 0 °C, and stirred for 20 h. After full consumption of the starting material, the solution was diluted with DCM (150 mL), washed with a saturated $NaHCO_3$ solution (20 mL). The aqueous layer was extracted twice with DCM, and the combined organic layers were washed with brine, dried over $Na_2SO_4$, and volatiles were removed under reduced pressure. Purification by silica gel column chromatography (pentane/EtOAc 10:1→3:1) afforded the desired product as an oil (452 mg, 0.944 mmol, 44% yield). $^{1}H$ NMR (500 MHz, $CDCl_3$): $\delta$ = 7.50 – 7.15 (m, 10H, 10CH Ar), 4.81 – 4.63 (m, 3H, 3CH*H*Ph), 4.55 (d, $J$ = 11.6 Hz, 1H, CH*H*Ph), 4.03 (dd, $J$ = 5.6, 2.8 Hz, 1H, H-2), 3.96 (dd, $J$ = 10.5, 4.0 Hz, 1H, C*H*H-6), 3.84 (dd, $J$ = 10.5, 5.2 Hz, 1H, CH*H*-6), 3.75 (dd, $J$ = 7.4, 5.6 Hz, 1H, H-3), 3.24 (t, $J$ = 6.9 Hz, 2H, $CH_2N_3$), 2.92 – 2.66 (m, 1H, OH), 2.32 – 2.20 (m, 1H, $C\mathit{H}HN_{aziridine}$), 2.10 (h, $J$ = 4.4 Hz, 1H, H-4), 2.06 (dd, $J$ = 5.1, 3.0 Hz, 1H, H-6), 2.02 (dd, $J$ = 5.1, 2.9 Hz, 1H, H-1), 2.00 – 1.88 (m, 1H, $CH\mathit{H}N_{aziridine}$), 1.62 – 1.46 (m, 4H, 4CH*H* linker), 1.40 – 1.14 (m, 13H, 8CH*H* linker, grease/pentane trace) ppm. $^{13}C$ NMR (126 MHz, $CDCl_3$): $\delta$ = 138.7, 138.6 ($2C_q$), 128.5, 128.0, 127.9, 127.8, 127.7 (6CH Ar), 85.9 (CH, C-2), 82.3 (CH, C-3), 72.7, 71.7 (2 $CH_2Ph$), 63.3 ($CH_2$, C-5), 58.8 ($CH_2N_{aziridine}$), 51.6 ($CH_2N_3$), 45.7 (CH, C-4), 43.1 (CH, C-6), 41.8 (CH, C-1), 29.7, 29.5, 29.2, 28.9, 27.3, 26.8 (6$CH_2$linker) ppm. HR-MS (ESI): *m/z* calcd for $C_{28}H_{39}N_4O_3^+$: 479.30167 $[M+H]^+$, found 479.30139.

**N-(8’-amino-octyl)-β-L-*arabino*furanosyl-cyclitol aziridine 7**

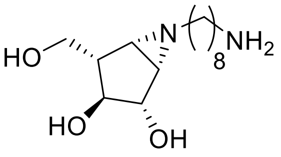

Azide **14** (136 mg, 0.284 mmol) was treated according to general procedure A, to yield titled product (59.5 mg, 0.219 mmol, 77% yield) as white powder upon lyophilisation. $^{1}$H NMR (500 MHz, $D_2O$): $\delta$ = 4.05 (dd, $J$ = 6.3, 2.2 Hz, 1H, H-2), 3.78 (dd, $J$ = 10.7, 4.9 Hz, 1H, C*H*HOH), 3.69 (dd, $J$ = 10.7, 9.6 Hz, 1H, CH*H*OH), 3.21 (dd, $J$ = 8.2, 6.5 Hz, 1H, H-3), 3.01 – 2.95 (m, 2H, C*H₂*NH₂), 2.35 – 2.26 (m, 3H, H-1, H-6, C*H*H$N_{aziridine}$), 2.11 – 2.00 (m, 2H, H-4, CH*H*$N_{aziridine}$), 1.64 (q, $J$ = 7.5 Hz, 2H, 2CH*H* linker), 1.55 – 1.46 (m, 2H, 2CH*H* linker), 1.41 – 1.26 (m, 8H, 8CH*H* linker) ppm. $^{13}$C NMR (126 MHz, $D_2O$): $\delta$ = 78.3 (CH, C-2), 75.9 (CH, C-3), 61.2 ($CH_2$, C-6), 57.6 ($CH_2N_{aziridine}$), 46.8 (CH, C-4), 44.5, 41.8 (2CH, C-1, C-6) 39.6 ($CH_2NH_2$), 28.6, 28.4, 28.1, 26.8, 26.3, 25.5 (6$CH_2$ linker) ppm. HR-MS (ESI): *m/z* calcd for $C_{14}H_{29}N_2O_3^{+}$: 273.21727 [*M*+H]$^{+}$, found 273.21710.

**β-L-*arabino*furanosyl-cyclitol aziridine Cy5-tagged ABP 8**

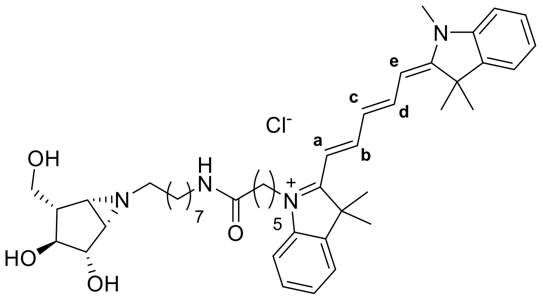

Amine **7** (27 mg, 100 μmol) was reacted according to general procedure B followed by reverse-phase HPLC purification with C18 column (A: 50 mM $NH_4HCO_3$; B: $CH_3CN$), to afford the titled product as a blue powder (5.8 mg, 7.5 μmol, 7.5% yield). After NMR analysis, the desired product was lyophilised to remove volatiles. $^{1}$H NMR (500 MHz, MeOD): $\delta$ = 8.25 (t, $J$ = 13.0 Hz, 2H, H-b, H-d), 7.50 (d, $J$ = 7.4 Hz, 2H, 2CH Ar), 7.42 (dddd, $J$ = 8.6, 7.5, 4.4, 1.2 Hz, 2H), 7.35 – 7.24 (m, 4H), 6.63 (t, $J$ = 12.4 Hz, 1H), 6.28 (dd, $J$ = 13.7, 5.7 Hz, 2H), 4.59 (s, 1H), 4.11 (t, $J$ = 7.4 Hz, 2H, $CH_2N^{+}$), 3.88 (dd, $J$ = 6.3, 3.0 Hz, 1H, H-2), 3.76 (dd, $J$ = 10.2, 4.4 Hz, 1H, C*H*H-5), 3.63 (s, 4H, $CH_3N$, CH*H*-5), 3.17 (dd, $J$ = 8.0, 6.3 Hz, 1H, H-3), 3.12 (t, $J$ = 7.2 Hz, 2H, C*H₂*NHC=O), 2.33 – 2.23 (m, 1H), 2.20 (t, $J$ = 7.3 Hz, 2H, $CH_2$C=O), 2.13 (dd, $J$ = 5.2, 2.8 Hz, 1H, H-6), 2.08 (dd, $J$ = 5.2, 3.0 Hz, 1H, H-1), 2.01 – 1.93 (m, 1H), 1.91 (s, 4H), 1.82 (q, $J$ = 7.6 Hz, 2H, 2CH*H* linker), 1.73 (s, 12H, 4$CH_3$), 1.71 – 1.64 (m, 2H, 2CH*H* linker), 1.59 – 1.50 (m, 2H, 2CH*H* linker), 1.49 – 1.40 (m, 4H, 4CH*H* linker), 1.37 – 1.26 (m, 8H, 8CH*H* linker) ppm. $^{13}$C NMR (126 MHz, MeOD): $\delta$ = 175.7, 175.4, 174.7 (3$C_q$), 155.5 (CH), 144.3, 143.6, 142.7, 142.5, 129.8 (5$C_q$), 129.7, 126.6, 126.3, 126.2, 123.4, 123.3, 112.0, 111.9, 104.4, 104.3 (10CHsp$^{2}$), 80.2 (CH, C-2), 77.4 (CH, C-3), 62.7 ($CH_2$, C-5), 59.7 ($CH_2N_{aziridine}$), 46.2 (CH, C-1), 44.8 ($CH_2N^{+}$), 43.0 (CH, C-6), 40.4 (*C*H₂NHC=O), 36.7 (*C*H₂C=O), 31.5 ($CH_3N$), 30.6, 30.4, 30.3, 28.3, 28.2, (6$CH_2$ linker), 27.9 ($CH_3$), 27.8 ($CH_3$), 27.4, 26.6, 23.4 (3$CH_2$ linker) ppm. HR-MS (ESI): *m/z* calcd for $C_{46}H_{65}N_4O_4^{+}$: 737.50003 [*M*]$^{+}$, found 737.49978.

**β-L-*arabino*furanosyl-cyclitol aziridine greenBODIPY-tagged ABP 9**

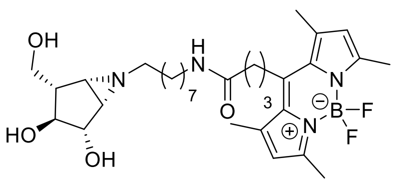

Amine **7** (11 mg, 40 µmol) was reacted according to general procedure B followed by silica gel column chromatography (DCM/MeOH 99:1→9:1 + 0.05% TEA), to afford the titled product as a red powder (11 mg, 18 µmol, 45% yield). After NMR analysis, the desired product was lyophilised to remove volatiles. $^1$H NMR (500 MHz, MeOD): *δ* = 6.14 (s, 2H, 2CH Ar), 3.87 (dd, *J* = 6.3, 3.0 Hz, 1H, H-2), 3.75 (dd, *J* = 10.2, 4.5 Hz, 1H, C*H*H-5), 3.65 (t, *J* = 9.9 Hz, 1H, CH*H*-5), 3.21 – 3.14 (m, 3H, H-3, C*H*$_2$NHC=O), 3.07 – 2.99 (m, 2H, CH$_2$), 2.45 (d, *J* = 4.3 Hz, 6H), 2.38 (t, *J* = 7.1 Hz, 2H, CH$_2$C=O), 2.30 – 2.20 (m, 1H, C*H*HN$_{aziridine}$), 2.11 (dd, *J* = 5.2, 2.8 Hz, 1H, H-6), 2.07 (dd, *J* = 5.2, 3.0 Hz, 1H, H-1), 1.99 – 1.83 (m, 3H, CH*H*N$_{aziridine}$, 2CH*H* linker), 1.53 (q, *J* = 12.3, 9.7 Hz, 4H, 4CH*H* linker), 1.41 – 1.28 (m, 8H, 8CH*H* linker) ppm. $^{13}$C NMR (126 MHz, MeOD): *δ* = 174.8, 155.2, 147.2, 142.4 (4C$_q$), 122.7 (CHsp$^2$), 80.2 (CH, C-2), 77.5 (CH, C-3), 62.7 (CH$_2$, C-5), 59.7 (CH$_2$N$_{aziridine}$), 46.2 (CH, C-1), 43.0 (CH, C-6), 40.5 (CH$_2$NH), 37.1 (*C*H$_2$C=O), 31.2 (CH, C-4), 30.6, 30.4, 30.3, 29.3, 28.6, 28.3, 28.0 (7CH$_2$ linker), 16.6 (CH$_3$), 14.4 (CH$_3$) ppm. HR-MS (ESI): *m/z* calcd for $C_{31}H_{48}BF_2N_4O_4^+$: 589.37367 [*M*+H]$^+$, found 589.37328.

**β-L-*arabino*furanosyl-cyclitol aziridine biotin-tagged ABP 10**

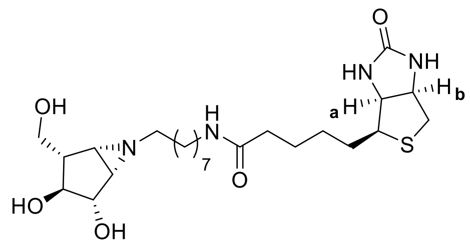

Amine **7** (29 mg, 108 µmol) was reacted according to general procedure B followed by reverse-phase HPLC purification (A: 50 mM $NH_4HCO_3$: B: $CH_3CN$), to afford the titled product as a white powder (4.24 mg, 8.5 µmol, 7.9% yield). After NMR analysis, the desired product was lyophilised to remove volatiles. $^1$H NMR (850 MHz, D$_2$O): *δ* = 4.54 – 4.50 (m, 1H, H-b), 4.34 (dd, *J* = 8.0, 4.5 Hz, 1H, H-a), 3.97 (dd, *J* = 6.5, 2.4 Hz, 1H, H-2), 3.69 (dd, *J* = 10.7, 4.8 Hz, 1H, C*H*H-5), 3.61 (dd, *J* = 10.7, 9.5 Hz, 1H, CH*H*-5), 3.24 (q, *J* = 4.4 Hz, 1H, CHS), 3.14 (dd, *J* = 8.1, 6.5 Hz, 1H, H-3), 3.13 – 3.04 (m, 2H, C*H*$_2$NHC=O), 2.91 (dd, *J* = 13.4, 5.9 Hz, 1H, C*H*HS), 2.70 (d, *J* = 13.0 Hz, 1H, CH*H*S), 2.29 – 2.21 (m, 3H, H-1, H-6, C*H*HN$_{aziridine}$), 2.16 (t, *J* = 7.1 Hz, 2H, CH$_2$C=O), 2.02 – 1.95 (m, 2H, H-5, CH*H*N$_{aziridine}$), 1.67 – 1.60 (m, 1H, C*H*HCHS), 1.61 – 1.56 (m, 1H, CH*H*CHS), 1.56 – 1.47 (m, 2H, C*H*$_2$CH$_2$C=O), 1.44 – 1.38 (m, 4H, C*H*$_2$CH$_2$NHC=O, C*H*$_2$CH$_2$N$_{aziridine}$), 1.35 – 1.28 (m, 2H, C*H*$_2$CH$_2$CHS), 1.25 – 1.17 (m, 8H, 8CH*H* linker) ppm. $^{13}$C NMR (214 MHz, D$_2$O): *δ* = 177.5, 166.2 (2C=O), 79.1 (CH, C-2), 76.6 (CH, C-3), 63.0 (CH, C-a), 62.0 (CH$_2$, C-5), 61.2 (CH, C-b), 58.4 (CH$_2$N$_{aziridine}$), 56.3 (CHS), 47.6 (CH, C-5), 45.5 (CH, C-6), 42.9 (CH, C-1), 40.6 (CH$_2$S), 40.2 (*C*H$_2$NHC=O), 36.5 (*C*H$_2$C=O), 29.5, 29.2, 28.7, 28.6, 27.3, 26.9, 26.1 (8CH$_2$ linker) ppm. HR-MS (ESI): *m/z* calcd for $C_{24}H_{43}N_4O_5S^+$: 499.29487 [*M*+H]$^+$, found 499.29486.

## 2.2 Crystallographic Data Collection and Refinement Statistics

### 2.2.1 Crystallisation and Soaking of 2 with *Bt*GH146 or HypBA1

***Bt*GH146** – *Bt*GH146 was initially co-crystallised with 2.5% (w/v) arabinose, as crystals grown without arabinose were found to be unsuitable for X-ray analysis, using an Oryx robot (Douglas instruments) with 150 nL protein solution plus 150 nL reservoir solution in 96-well format plates (MRC 2-well crystallization microplate, Swissci, Switzerland) equilibrated against 60 µL reservoir solution which consisted of 19% PEG 3350, 2-(*N*-morpholino)ethanesulfonic acid (MES)/Bis-Tris-propane mix with pH 6.0 – 6.25, 0.2 M ammonium formate. The crystals obtained were in the form of clusters of plates; these were used for microseed matrix screening (MMS)[3], which was carried out using the Oryx robot according to published protocols.[4-5] Briefly, the crystals were vortexed for 1 min with a Seed Bead (Hampton Research) with 50 µL well solution added. Crystals suitable for soak experiments were obtained after several rounds of seeding using 150 nL protein solution plus 50 nL seeding stock plus 100 nL well solution, with the latter consisting of 15 – 17% PEG 3350, MES pH 6.25-6.5, 0.2 M ammonium formate. They were soaked with **2** for 2 – 4 h as follows: 2 µL of a 5 mM solution of **2** were diluted with 8 µL mother liquor of the corresponding crystallisation condition giving a final concentration of 1 mM; approximately 0.2 – 0.5 µL of this solution were added to the drop containing crystals.

**HypBA1** – HypBA1 crystals were grown by the sitting drop vapour diffusion method, using protein at 37 mg/mL in 10 mM HEPES pH 7.5, in a volume ratio of 0.6:0.5 with the well solution, which consisted of 0.7 M sodium citrate, 0.1 M MES pH 6.5, 10 mM dithiothreitol. The crystal was soaked with **2** which had been dissolved at 20 mM in water, diluted with well solution to 4 mM, and added to the protein drop to a final concentration of 2 mM. The crystal was fished after 22 h, via a cryoprotectant solution comprised of the well solution components supplemented with 25% (v/v) glycerol, into liquid nitrogen.

**X-ray data collection and structure solution**

Diffraction data for HypBA1:**2** and *Bt*GH146:**2** complexes were collected at Diamond Light Source (UK) to 2.35 Å and 2.0 Å resolution, respectively, processed with XDS[6] and DIALS, respectively, as incorporated in Xia2[7,8] pipeline, and scaled with *AIMLESS*. For *Bt*GH146:**2**, the space group was $P2_12_12_1$, with unit cell dimensions of $a$ = 93.84, $b$ = 98.549, $c$ = 196.584 Å, and $\alpha = \beta = \gamma = 90.0°$. All computations were carried out using programs from the CCP4 suite[9] unless otherwise stated, as incorporated in CCP4Cloud user interface.[10] The structure was solved using MOLREP[11] with PDB entry 6YQH as an initial model and refined with cycles of REFMAC[12] alternating with manual model correction in COOT.[13] The dictionaries for the ligand and its covalent linkage were created in JLigand[14], and the ligand was built using COOT.

It should be noted that residue numbering for r*Bt*GH146: structure used in the present paper is shifted by +2 with respect to the sequence numbering in the corresponding Uniprot entry A0A6I0SRL5. This is an artificial numbering introduced for consistency when comparing with the PDB entry 5opj (the first reported structure of the enzyme), where the accidental sequence mismatch went unnoticed resulting in a gap between residues H23 and Q26 (that should have been Q24).

For HypBA1:**2**, the space group was $P3_2$ 2 1, with unit cell dimensions of 77.26, 77.26, 254.65 Å and $\alpha = \beta = 90.0°$ $\gamma = 120.0°$. As the structure was sufficiently isomorphous with PDB entry 7DIF it was solved using REFMAC[12] with the protein chain of this model. The ligand was built and dictionary restraints generated using *AceDRG*.[15] The structure was refined by performing cycles of manual building in COOT[13], interspersed with refinement using REFMAC5. The programs were run in the *CCP4i2* suite.[16]

**Table S3.** Data collection and refinement statistics (molecular replacement). Values for the outer shell are given in parentheses.

| | HypBA1 – **2** (PDBID: 8QF2) | *Bt*GH146 – **2** (PDBID: 8QF8) |
|---|---|---|
| **Data collection** | | |
| Space group | $P3_2$ 2 1 | $P2_12_12_1$ |
| Cell dimensions | | |
| *a*, *b*, *c* (Å) | 77.3, 77.3, 254.6 | 93.8, 98.5,196.6 |
| α, β, γ (°) | 90.0, 90.0, 120.0 | 90.0, 90.0, 90.0 |
| Resolution range (Å) | 85.03 – 2.35 (2.41 - 2.35) | 55.9 – 2.4 (2.45 – 2.4) |
| Total no. reflections | 758699 (73353) | 985632 (55562) |
| No. unique reflections | 35850 (3624) | 72064 (4369) |
| $R_{sym}$ or $R_{merge}$ | 0.200 (4.148) | 0.101 (1.138) |
| $R_{pim}$ | 0.046 (0.938) | 0.029 (0.343) |
| $CC_{1/2}$ | 0.995 (0.660) | 0.994 (0.830) |
| $I / \sigma I$ | 7.7 (0.7) | 16.8 (2.2) |
| Completeness (%) | 100.0 (100.0) | 100.0 (100.0) |
| Multiplicity | 20.0 (20.2) | 13.7 (12.7) |
| **Refinement** | | |
| No. reflections working set | 35892 | 71982 |

| | | |
|---|---|---|
| No. reflections test set | 1886 | 3622 |
| $R_{work}$ / $R_{free}$ | 0.21/0.25 | 0.19/0.22 |
| No. atoms | | |
| Protein | 5096 | 24162 |
| Ligand/ion | 11 | 44 |
| Water | 74 | 312 |
| Average *B*-factors (Å$^2$) | | |
| Protein | 72.8 | 65.1 |
| Ligand/ion | 63.1 | 49.5 |
| Water | 55.3 | 49.6 |
| R.m.s deviations | | |
| Bond lengths (Å) | 0.0064 | 0.0048 |
| Bond angles (°) | 1.407 | 1.259 |
| Molprobity score | 1.60 | 0.75 |
| Ramachandran plot residues | | |
| In most favorable regions (%) | 93.6 | 97.5 |
| In allowed regions (%) | 4.9 | 2.4 |
| Outliers (%) | 1.5 | 0.1 |

## 3. NMR Spectra

$^1H$ NMR and $^{13}C$ NMR of compound **12** in $CDCl_3$:

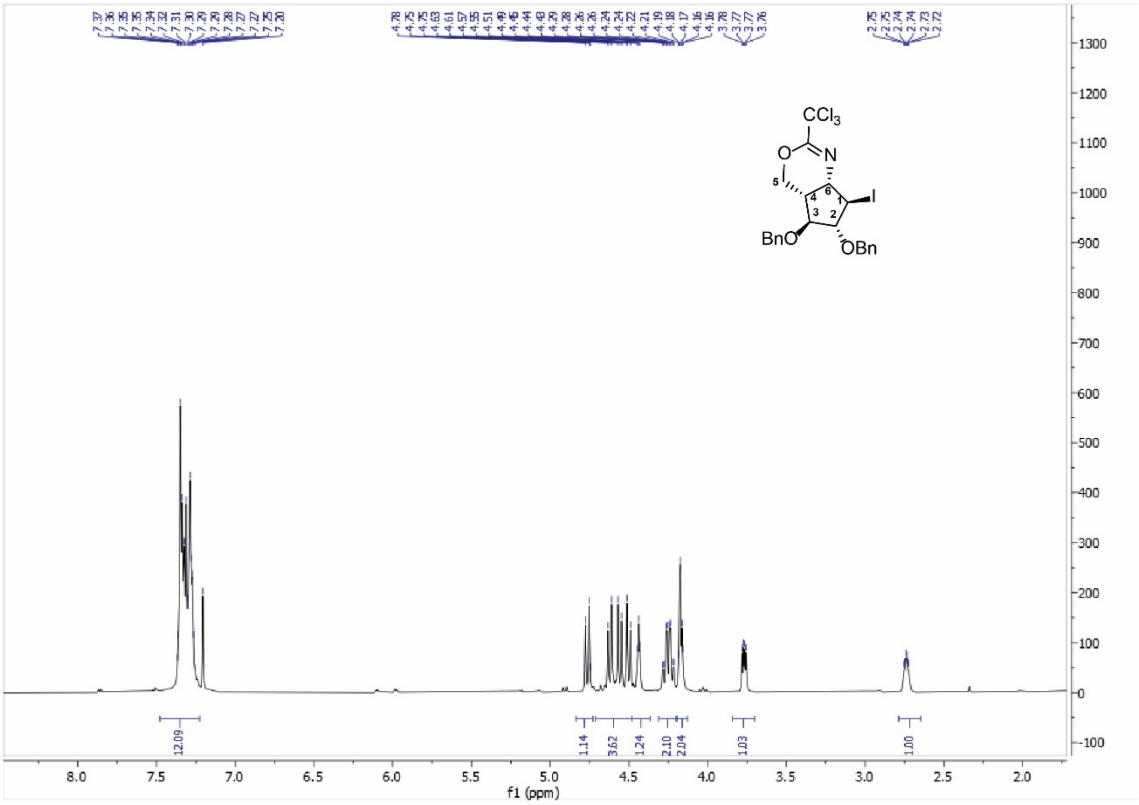

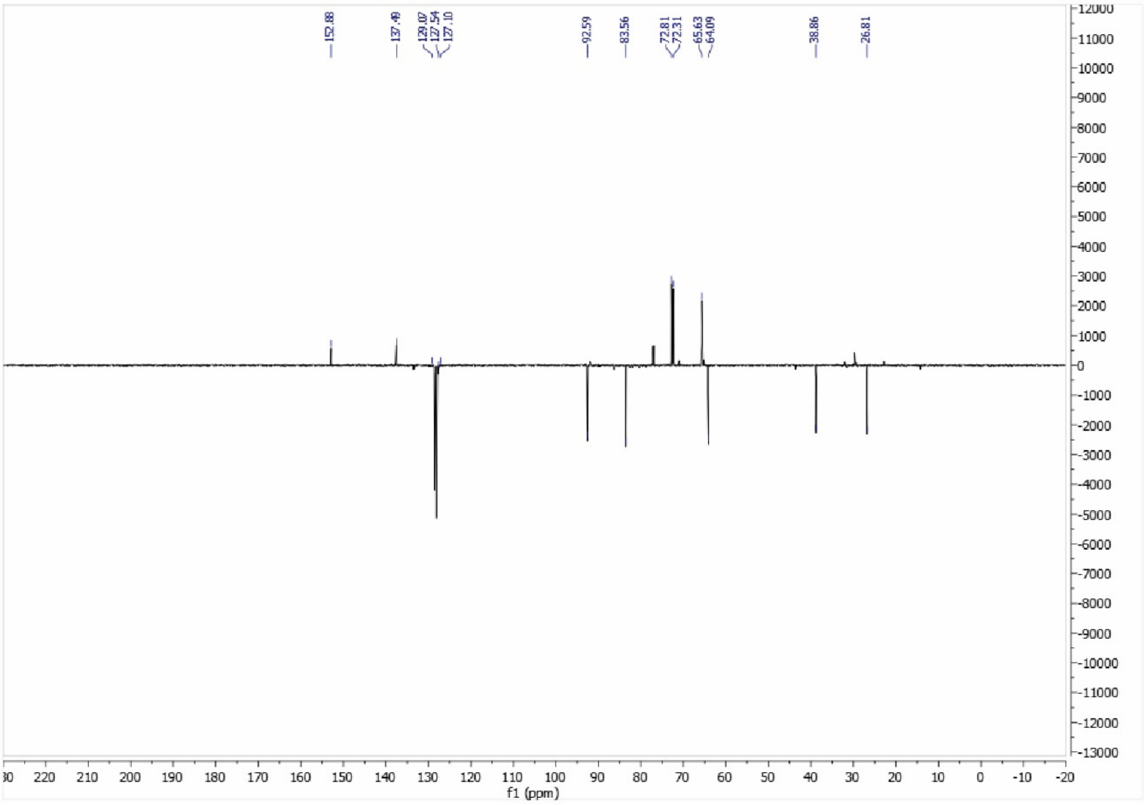

[1]H NMR and [13]C NMR of compound **13** in $CDCl_3$:

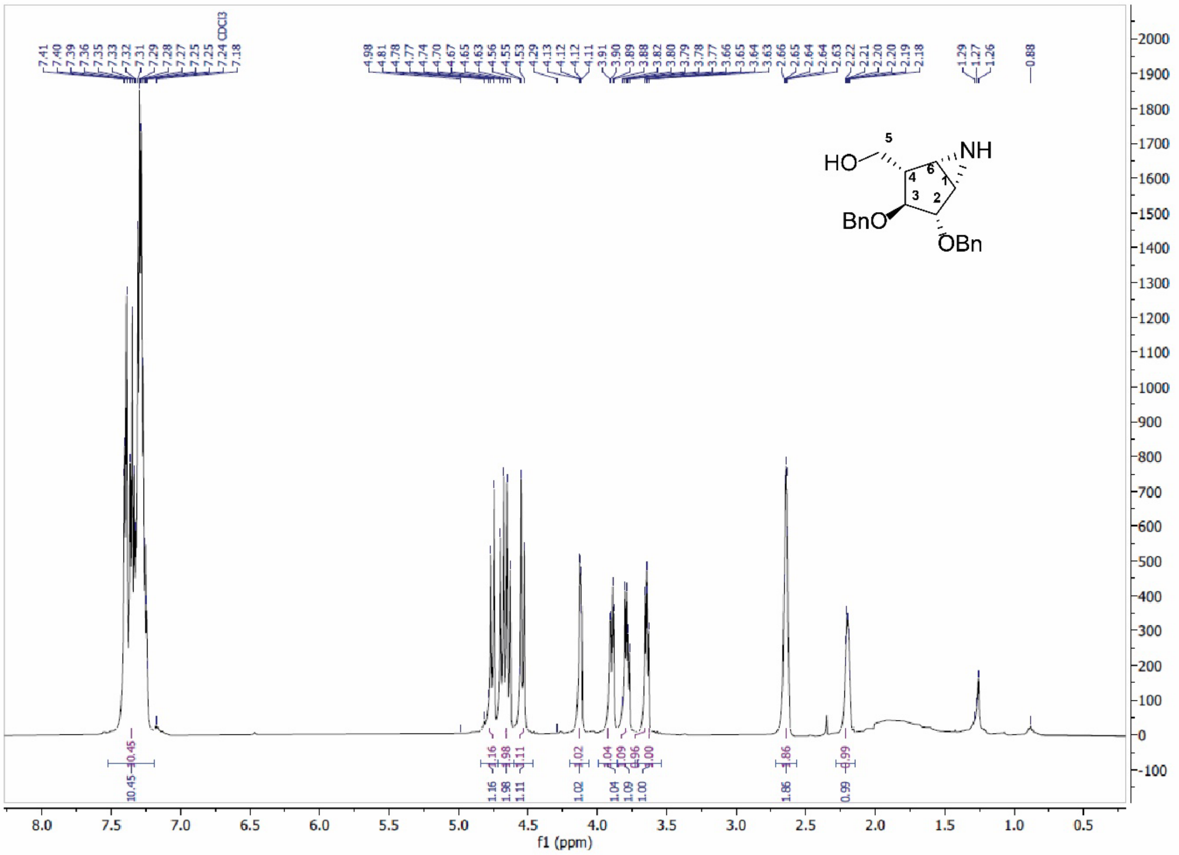


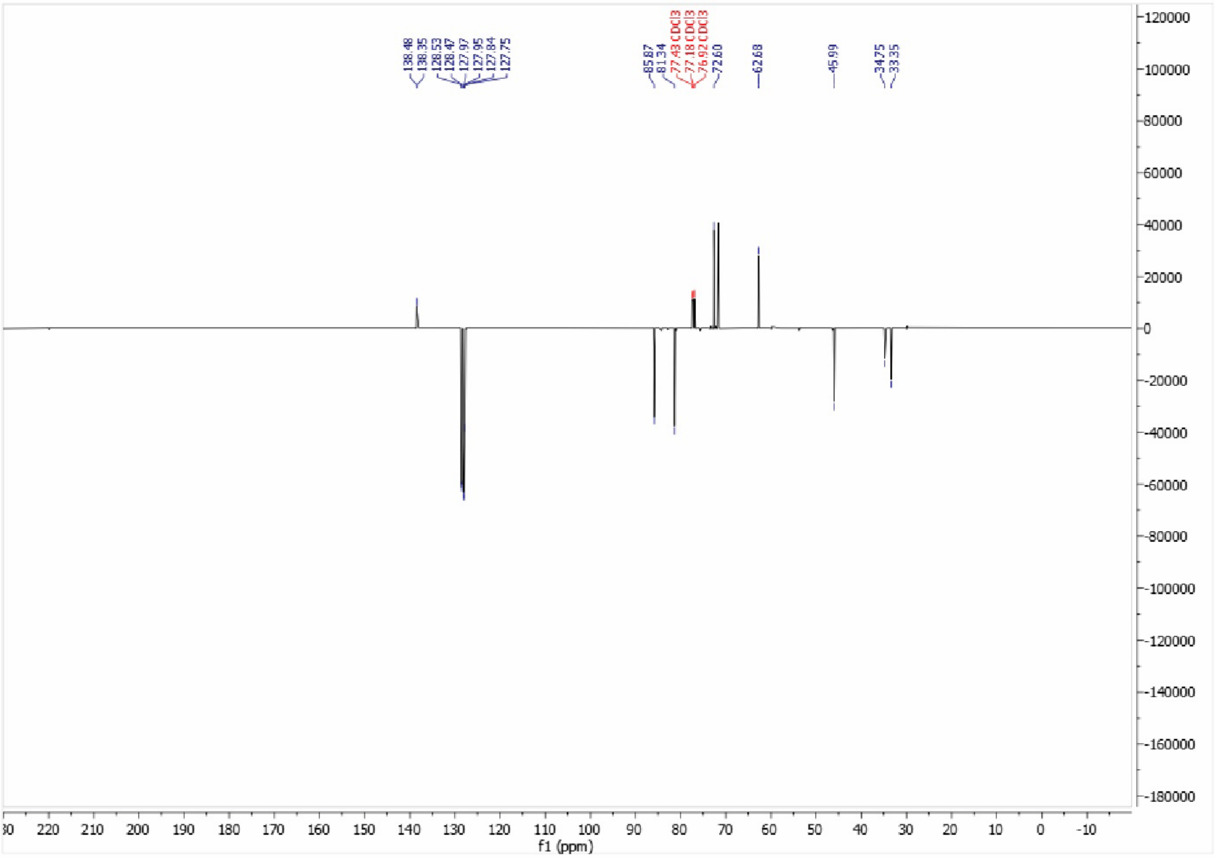

$^{1}H$ NMR and $^{13}C$ NMR of compound **2** in $D_2O$:

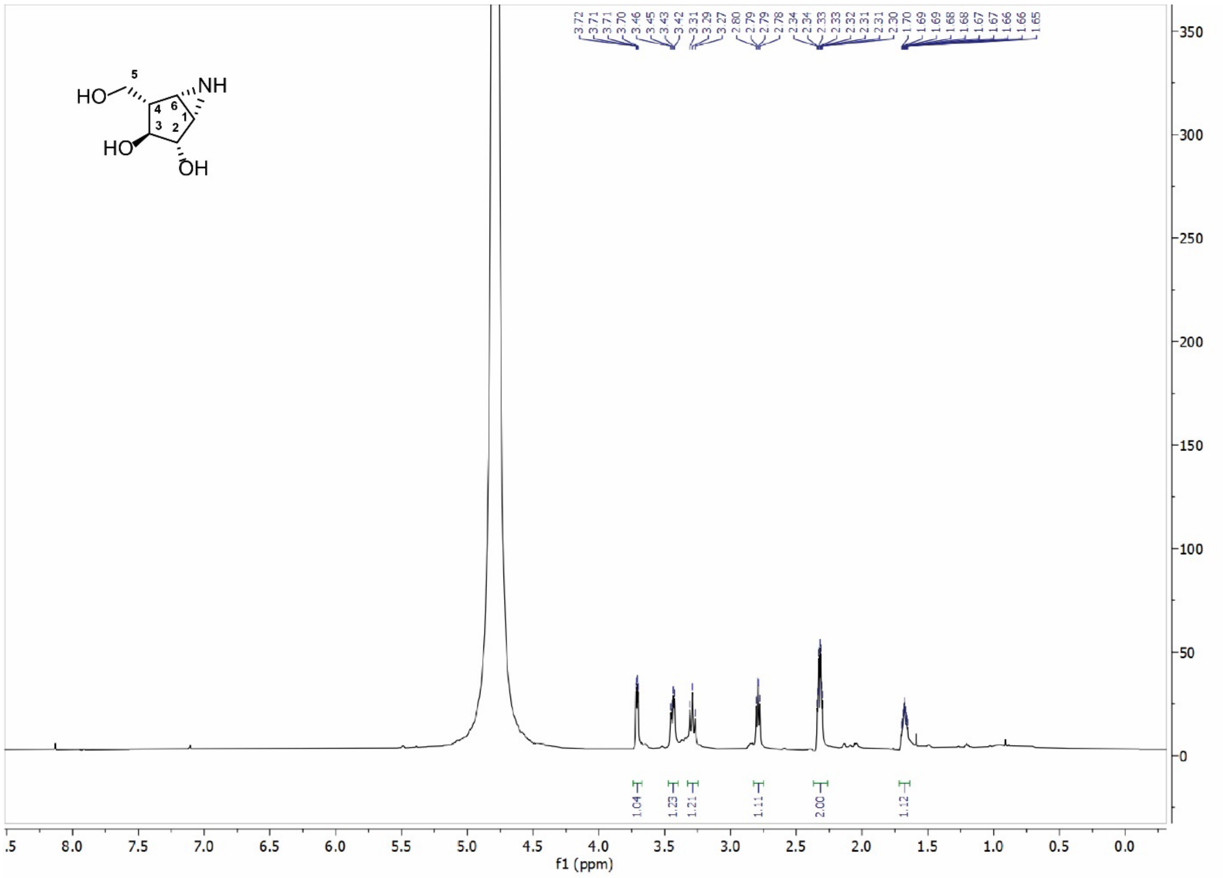


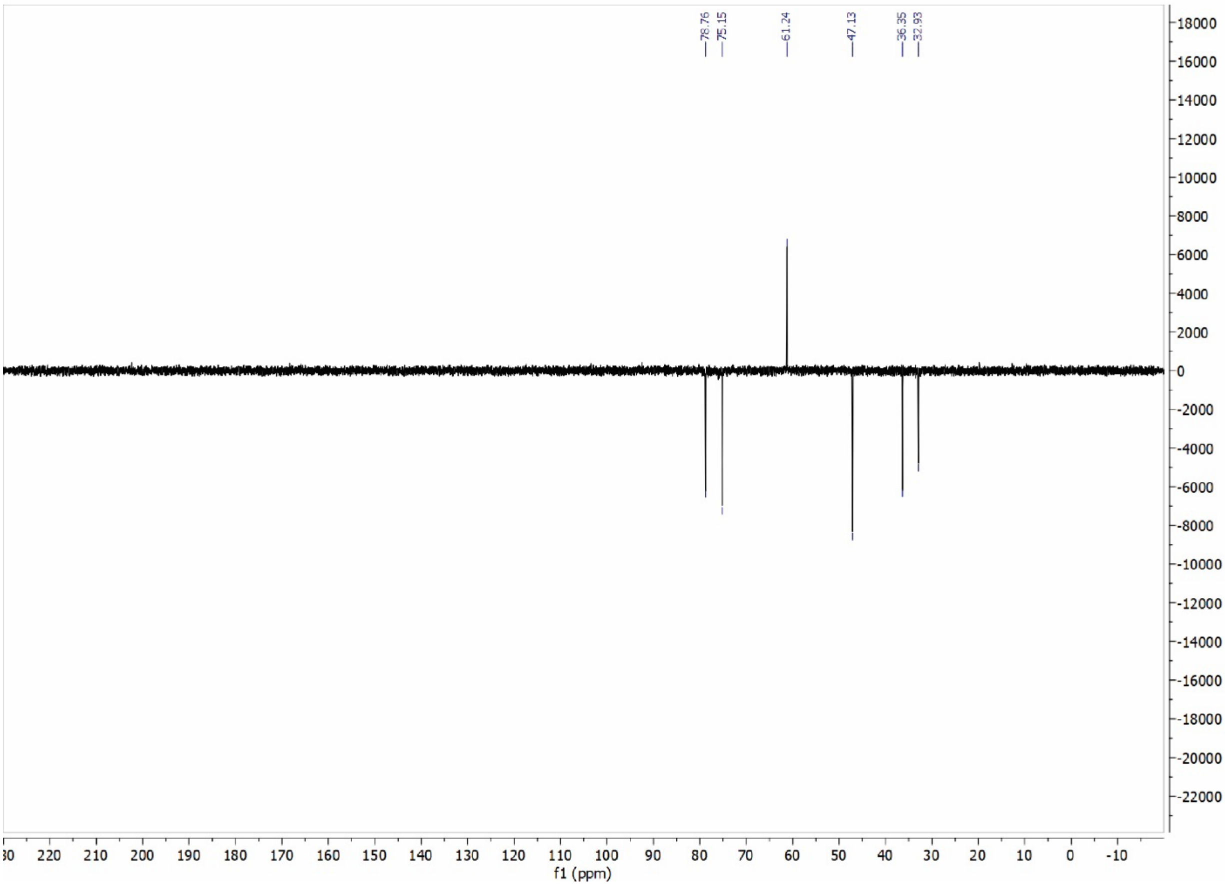

<sup></sup>

[1]H NMR and [13]C NMR of compound **14** in $CDCl_3$:

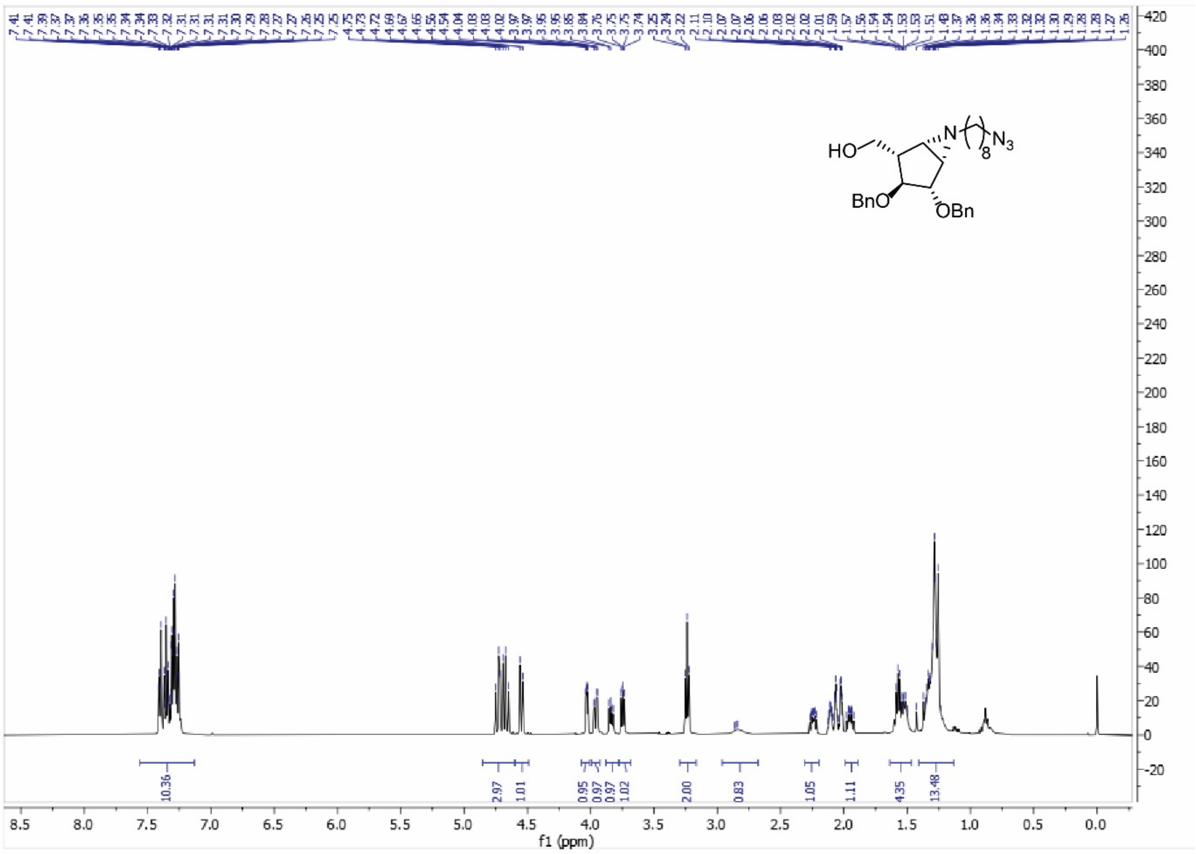

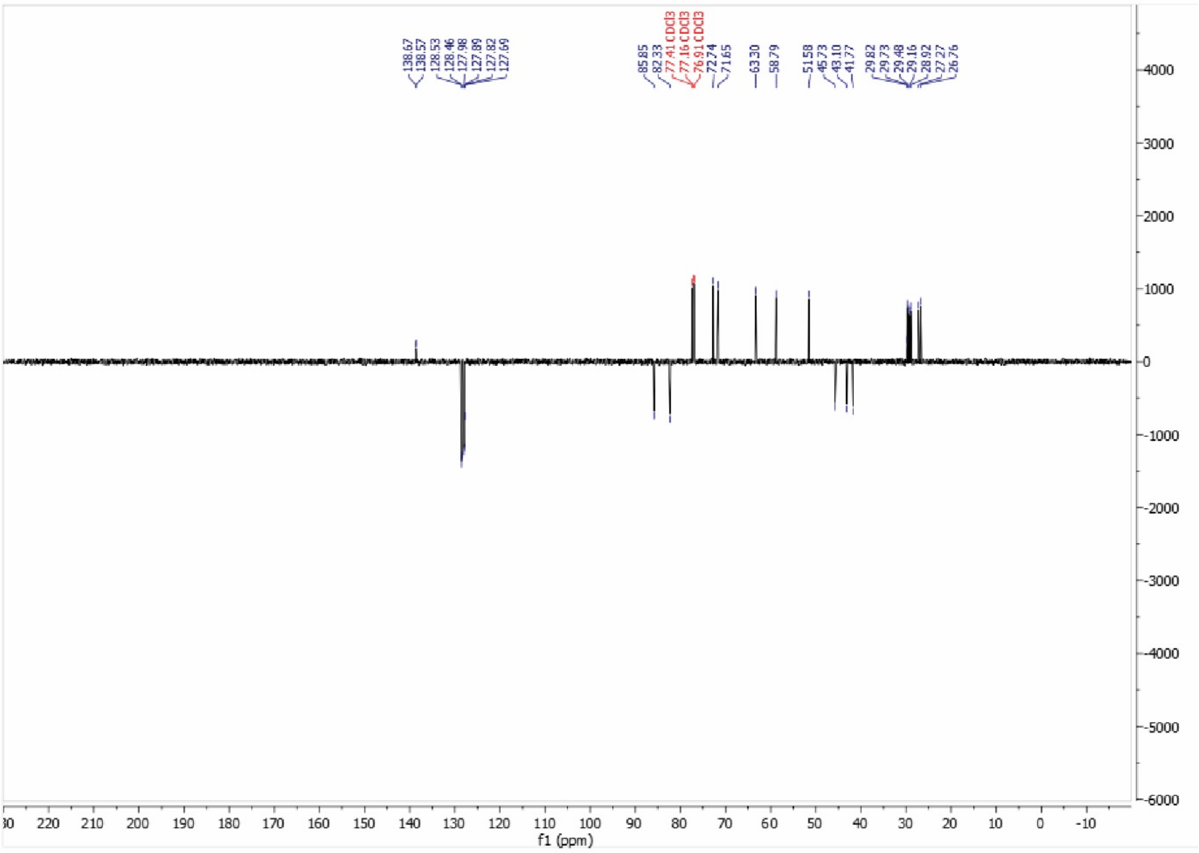

[1]H NMR and [13]C NMR of compound **7** in $D_2O$:

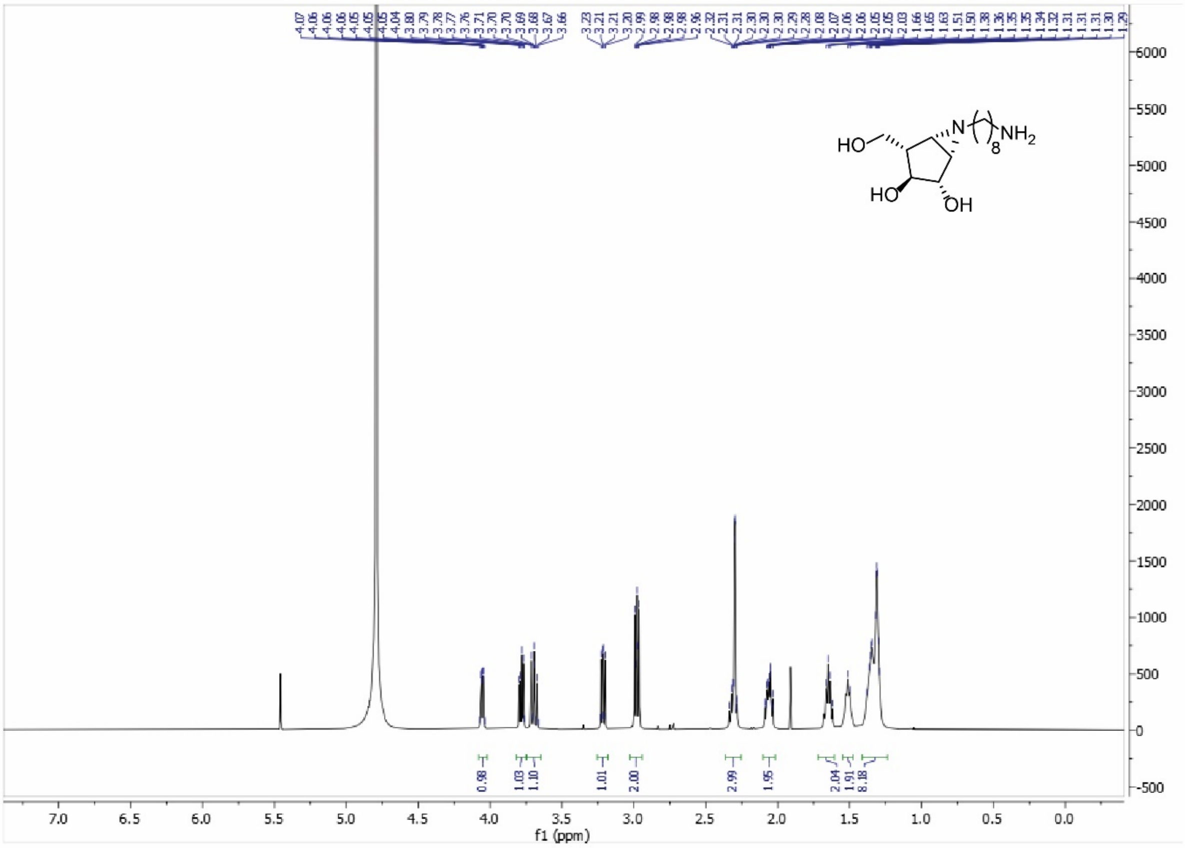

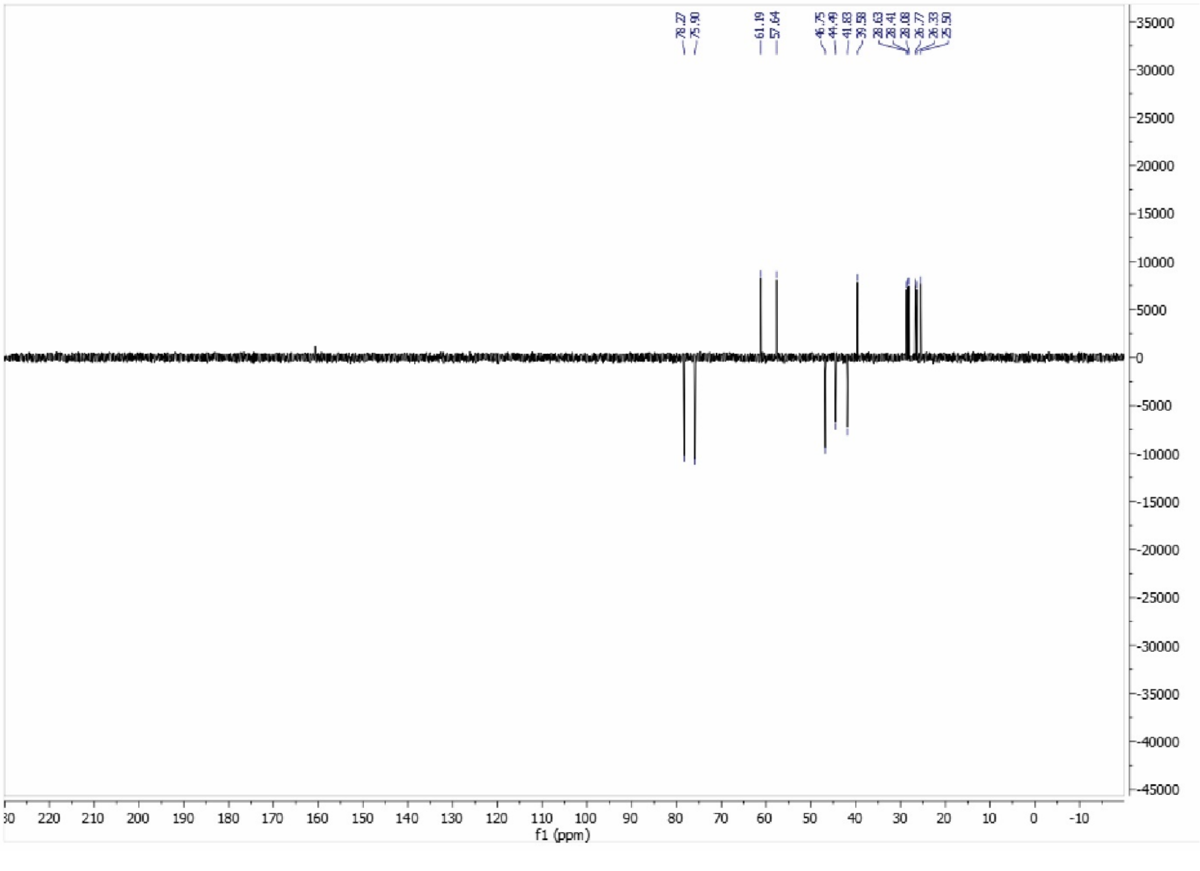

[1]H NMR and [13]C NMR of compound **8** in MeOD:

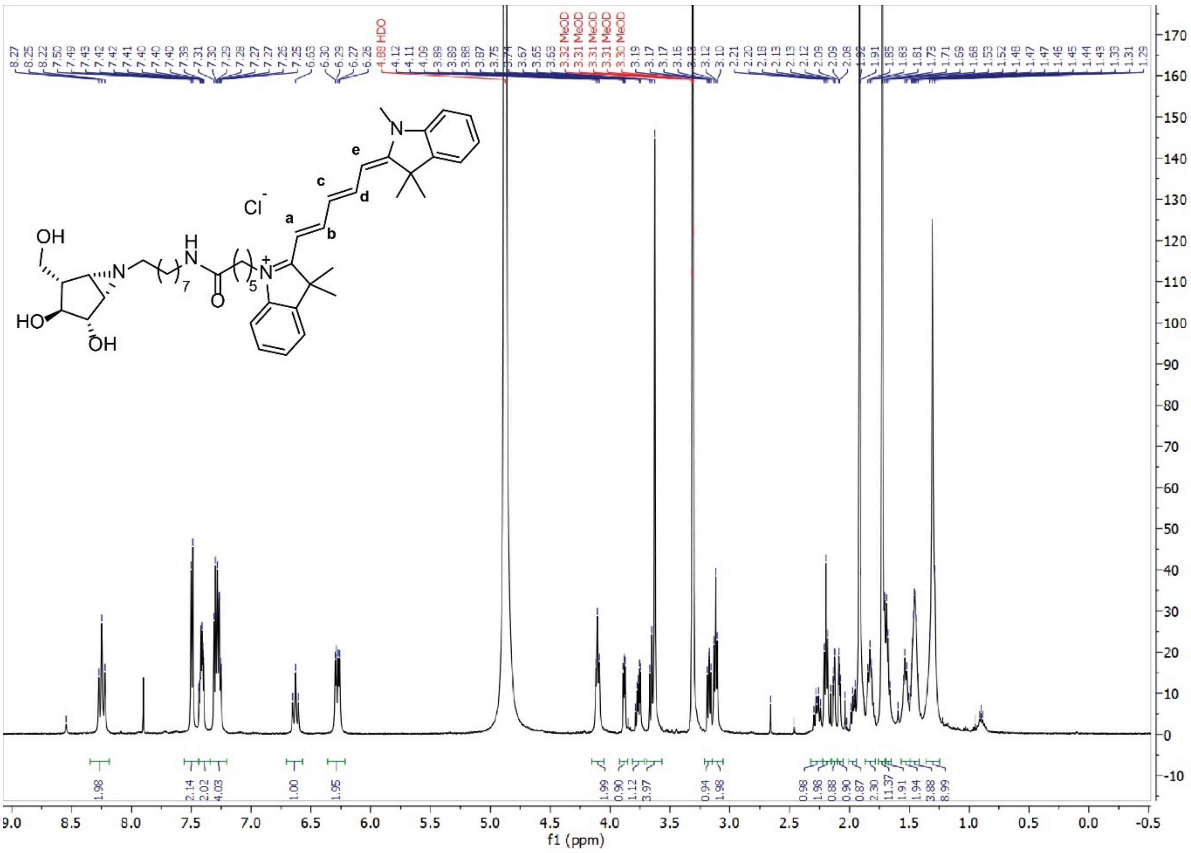

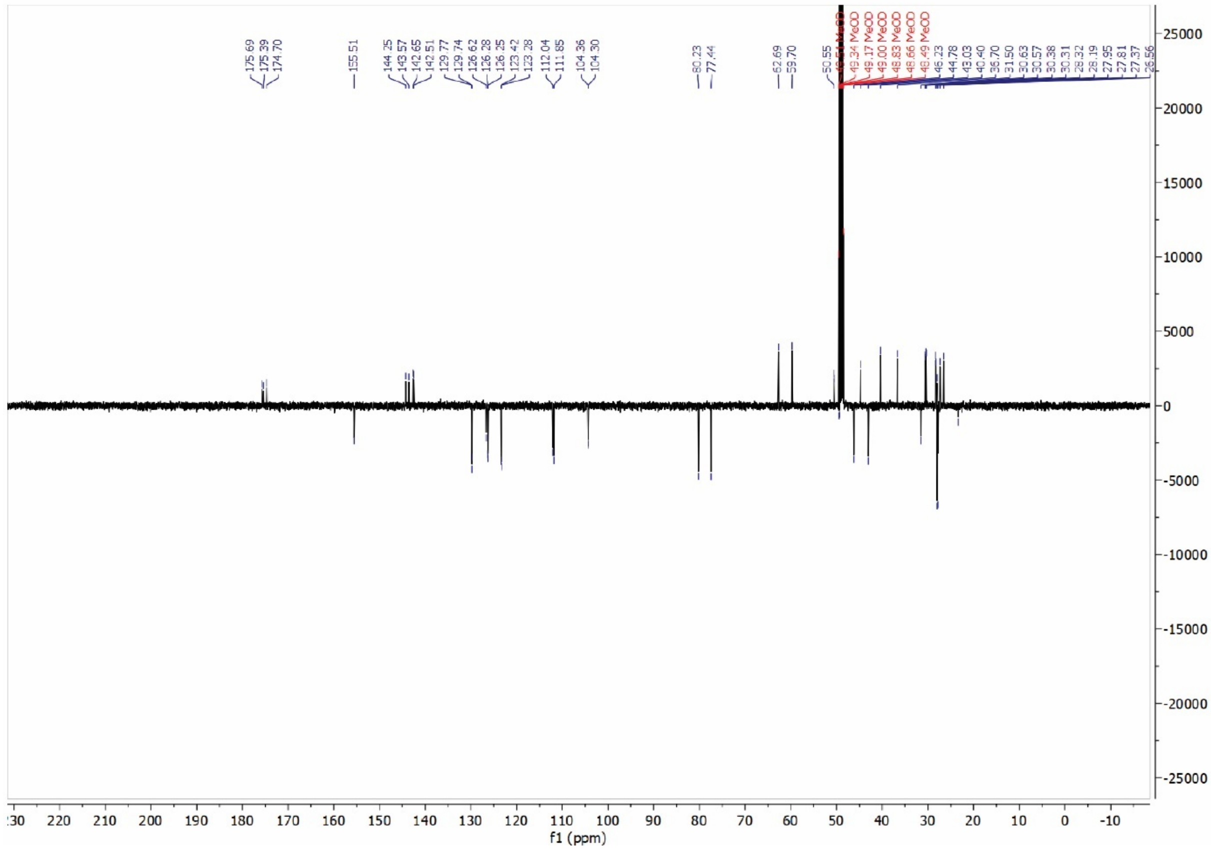

$^{1}$H NMR and $^{13}$C NMR of compound **9** in MeOD:

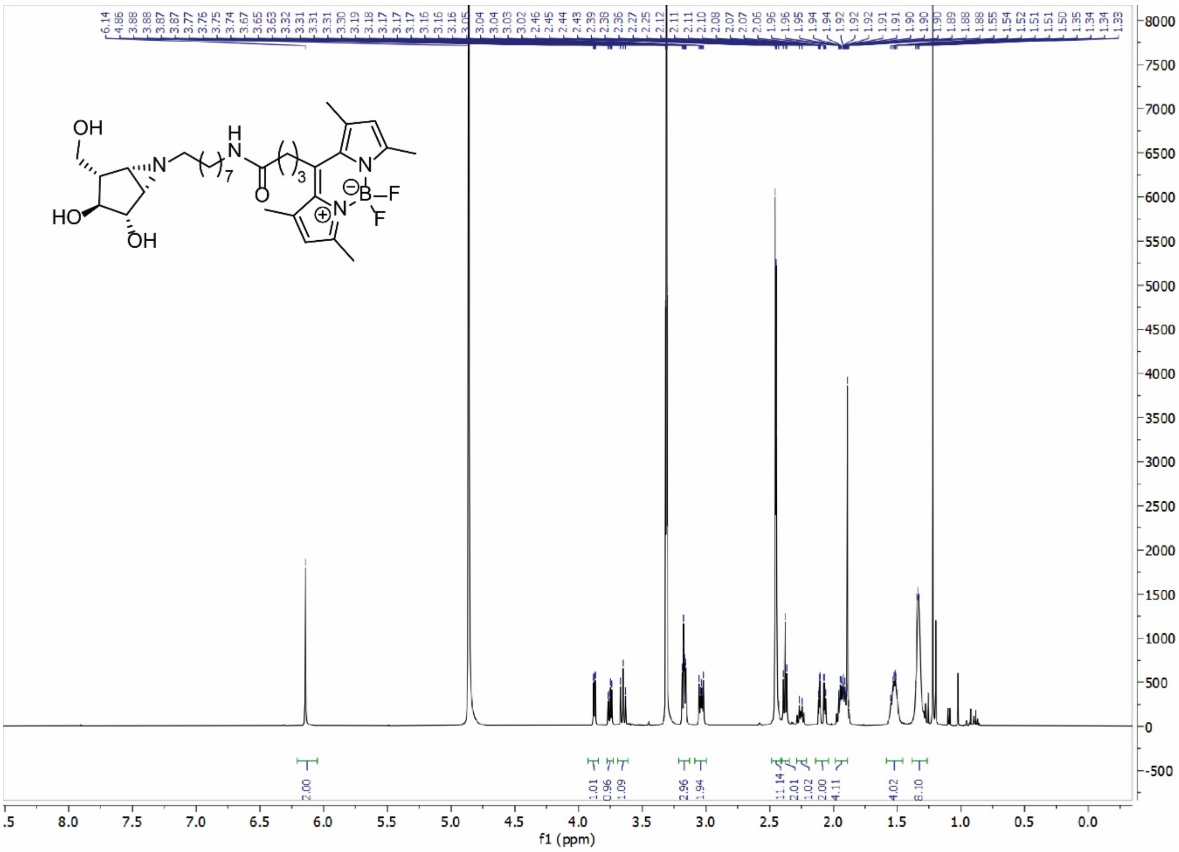


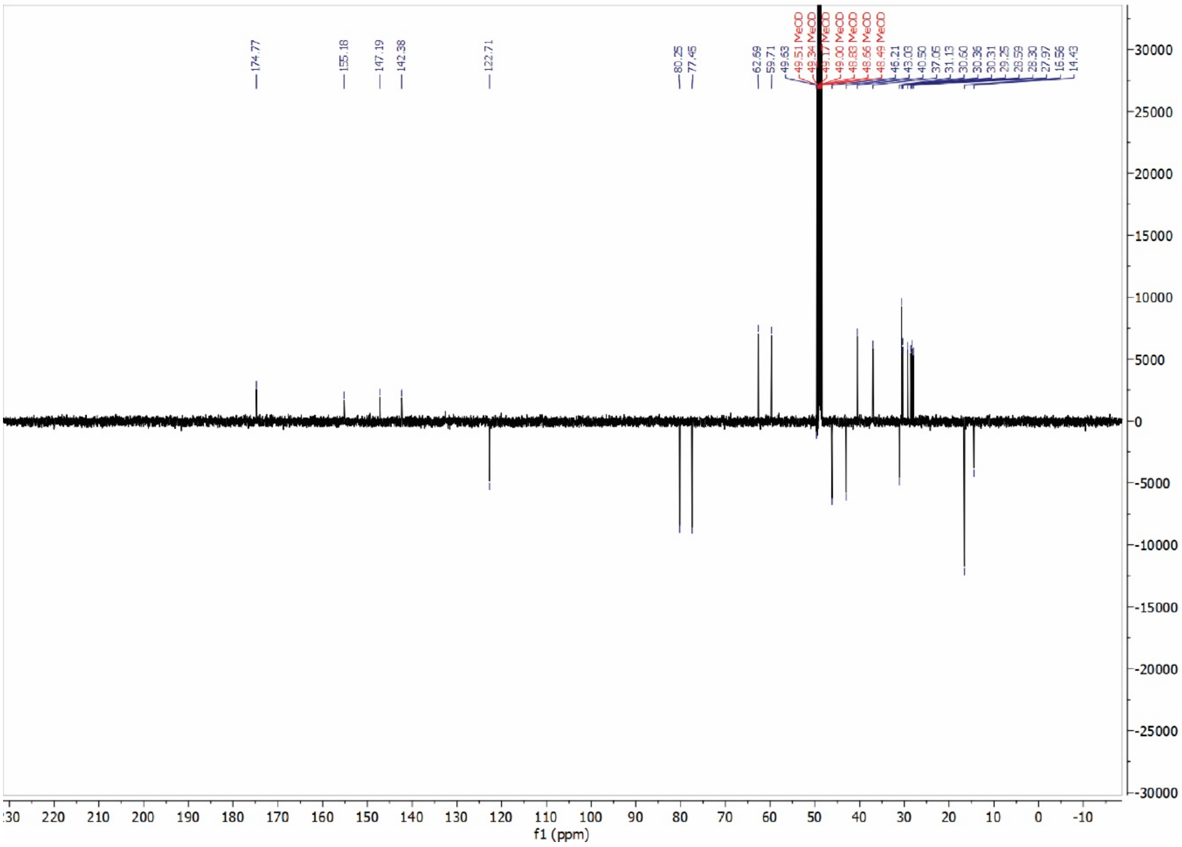

$^{1}$H NMR and $^{13}$C NMR of compound **10** in $D_2O$:

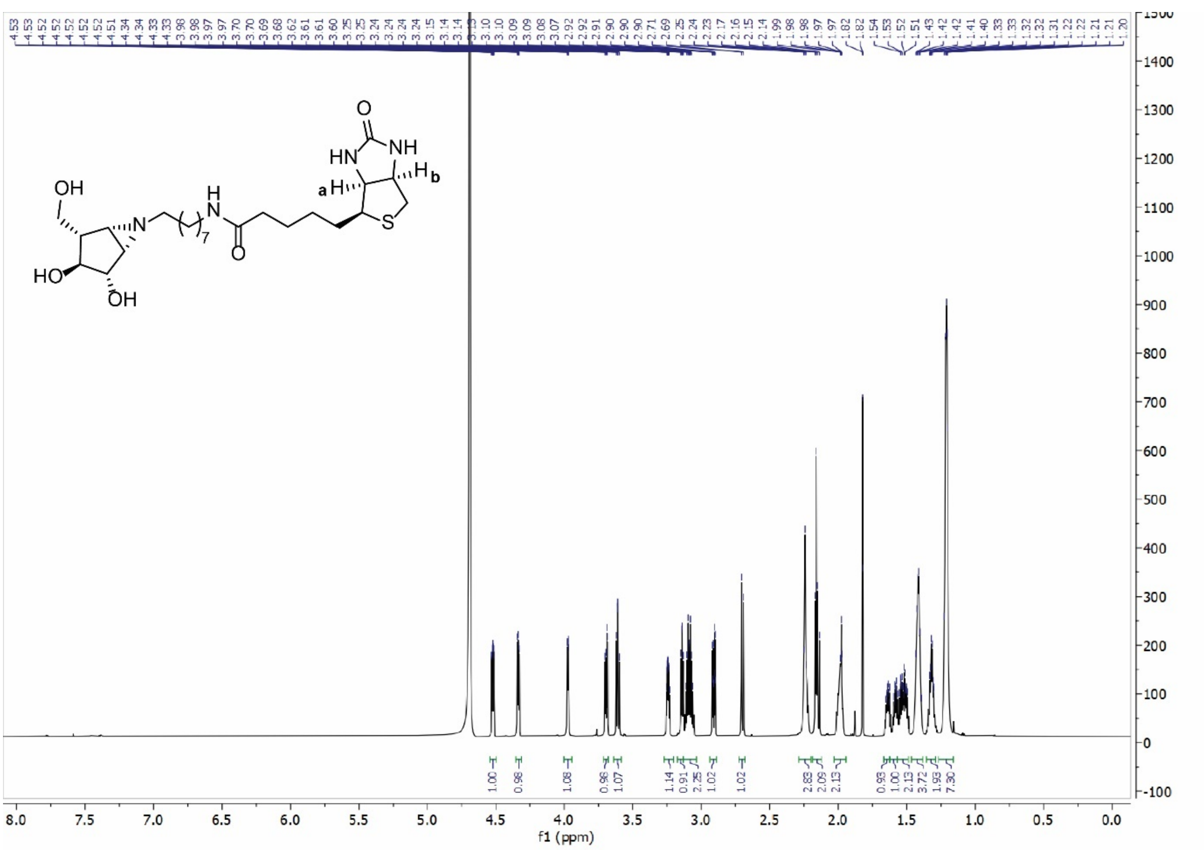

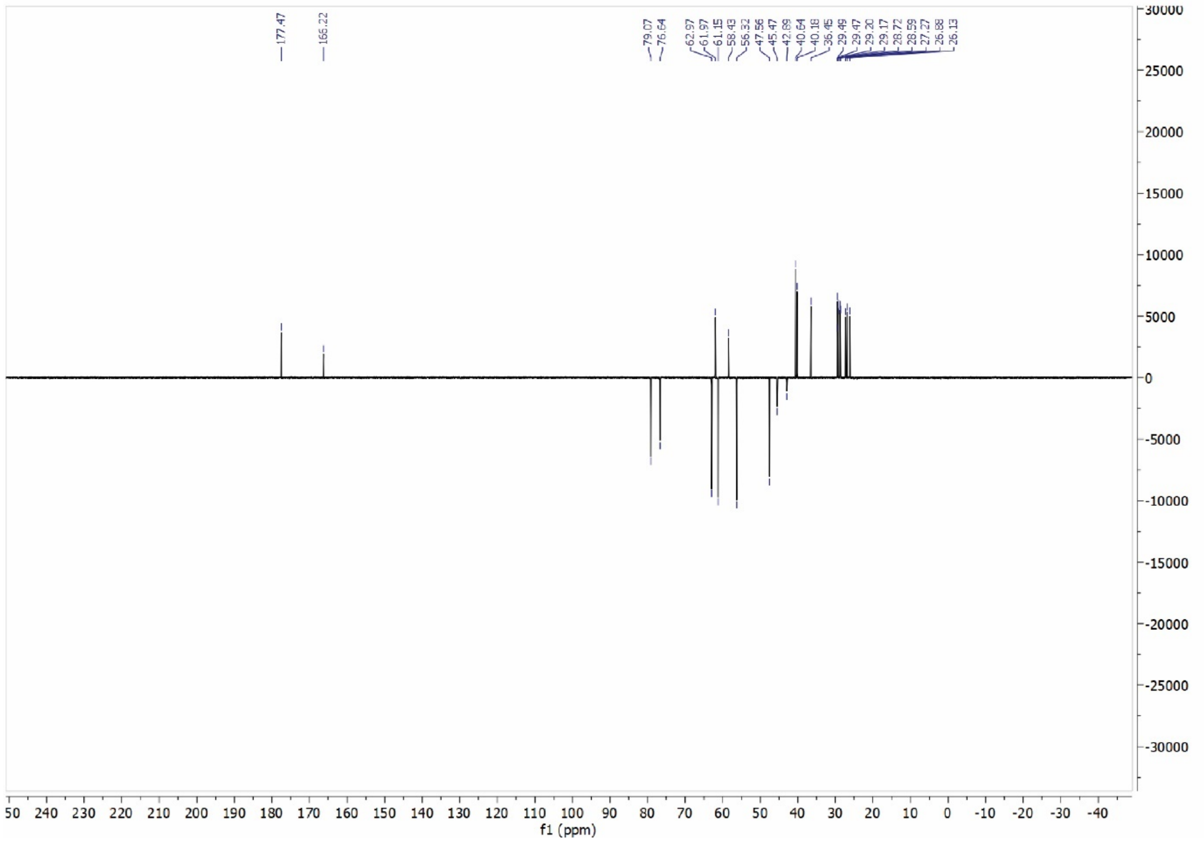

$^1H$ NMR and $^{13}C$ NMR of compound **16** in $CDCl_3$:

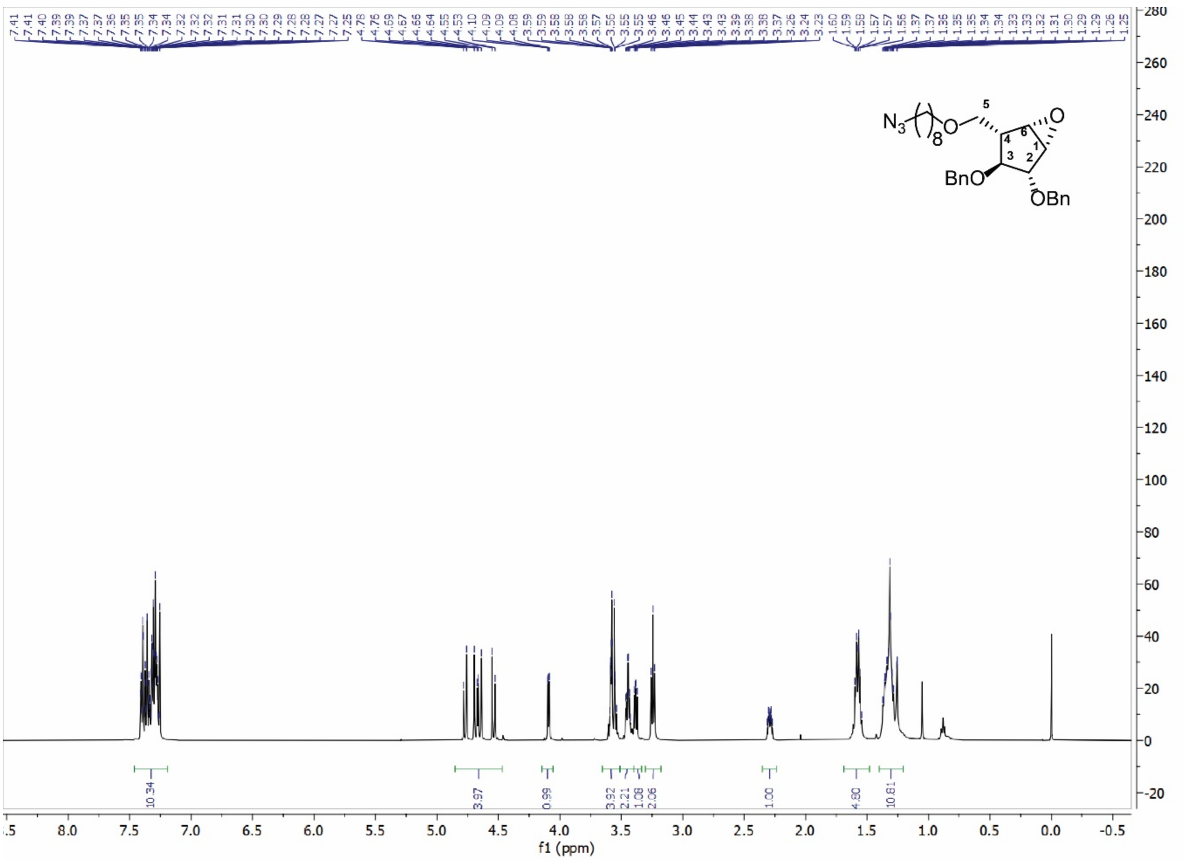

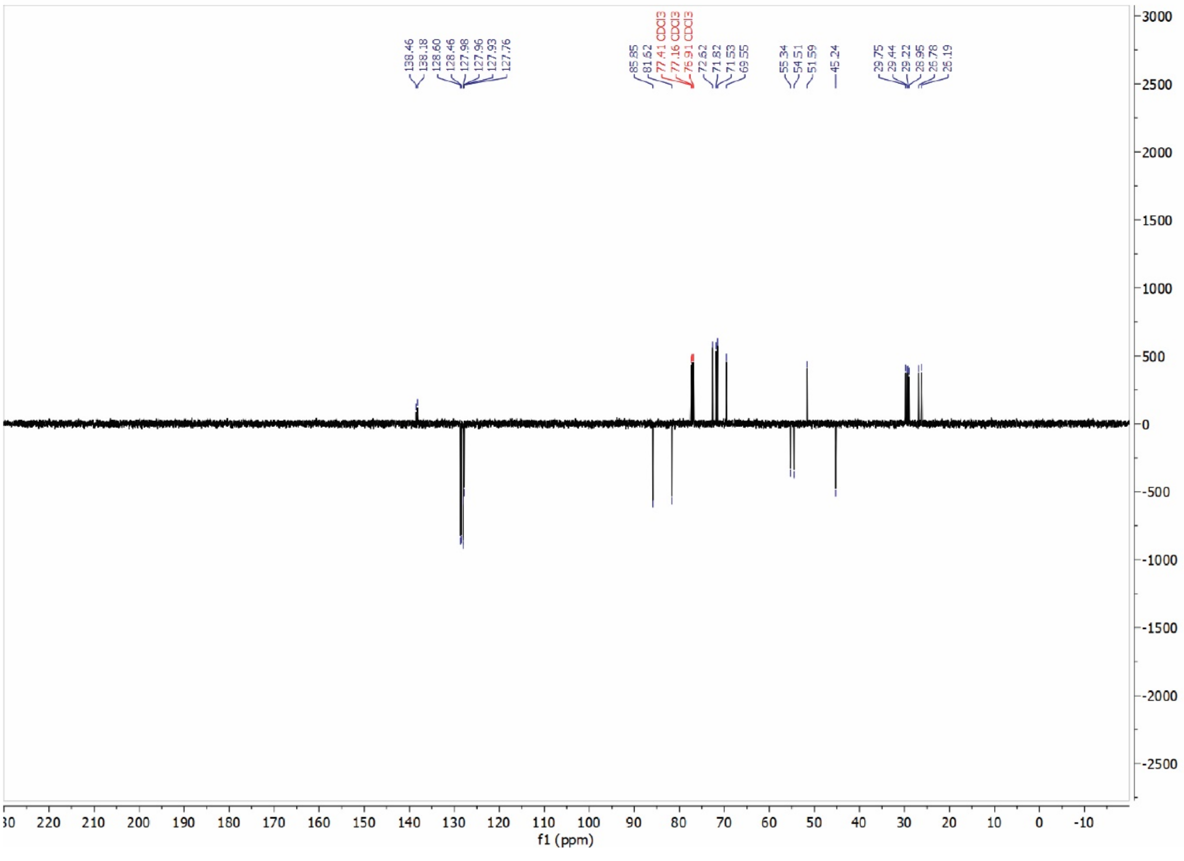

$^{1}H$ NMR and $^{13}C$ NMR of compound **17** in $CDCl_3$:

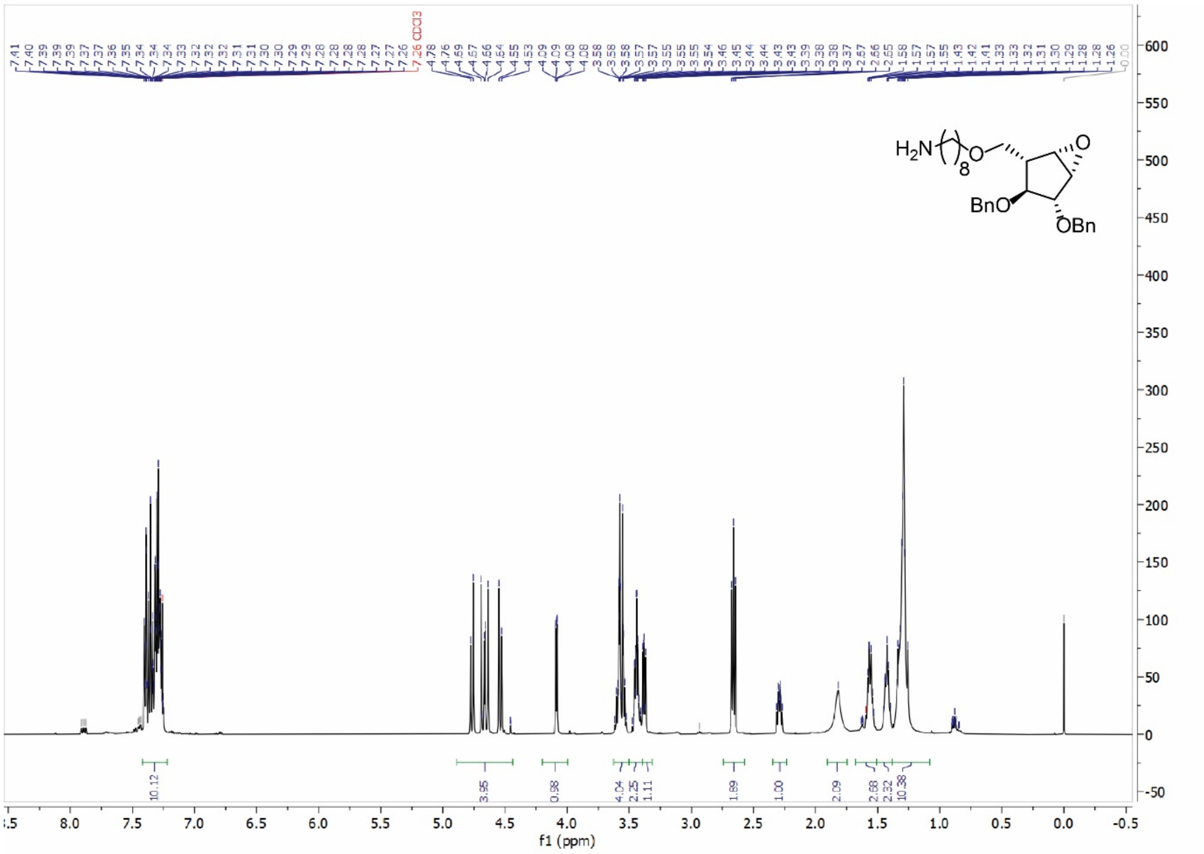

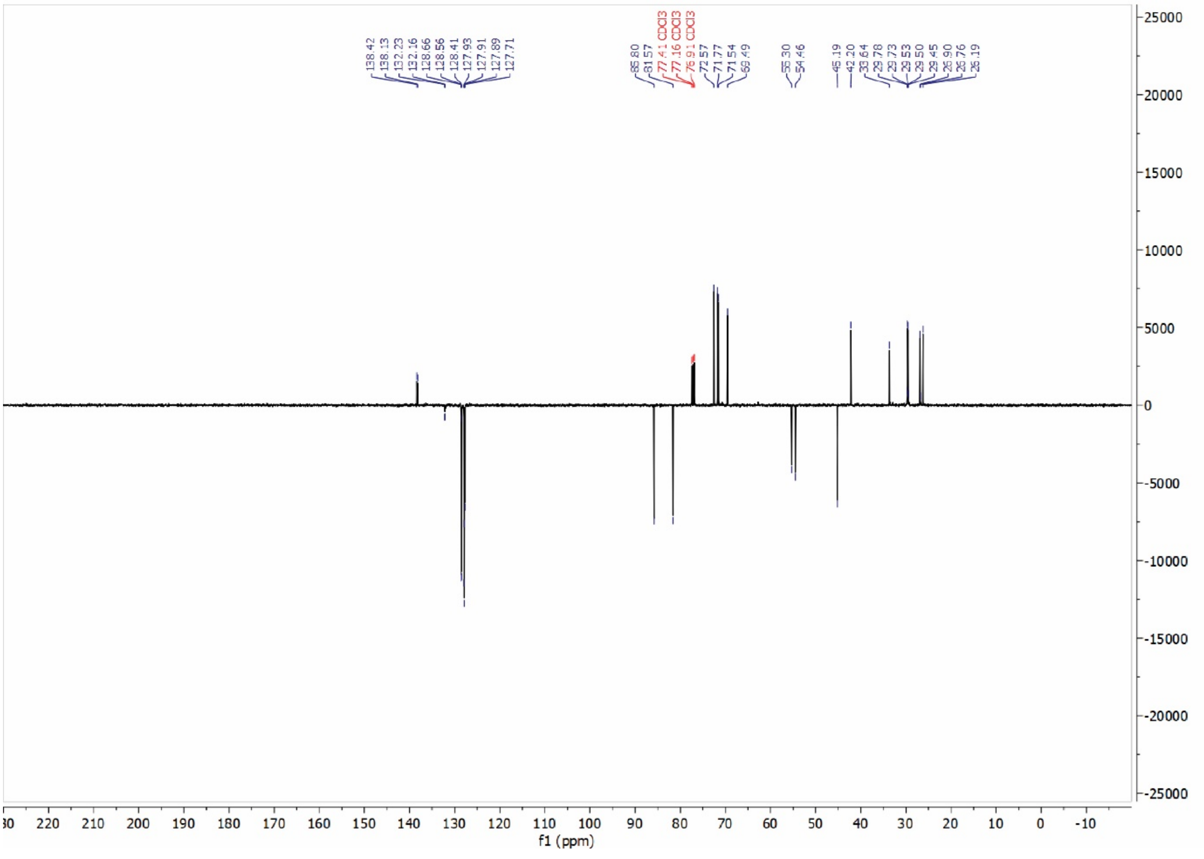

$^1H$ NMR and $^{13}C$ NMR of compound **3** in MeOD:

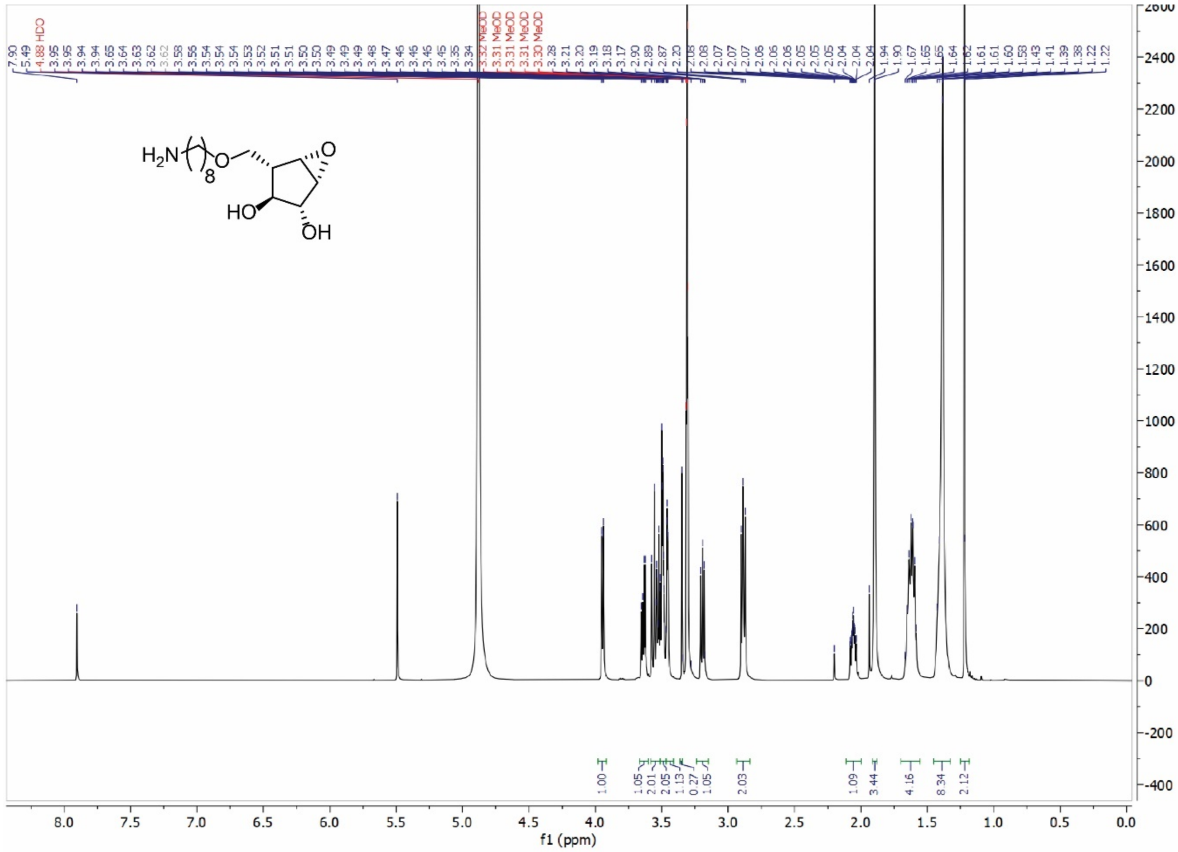


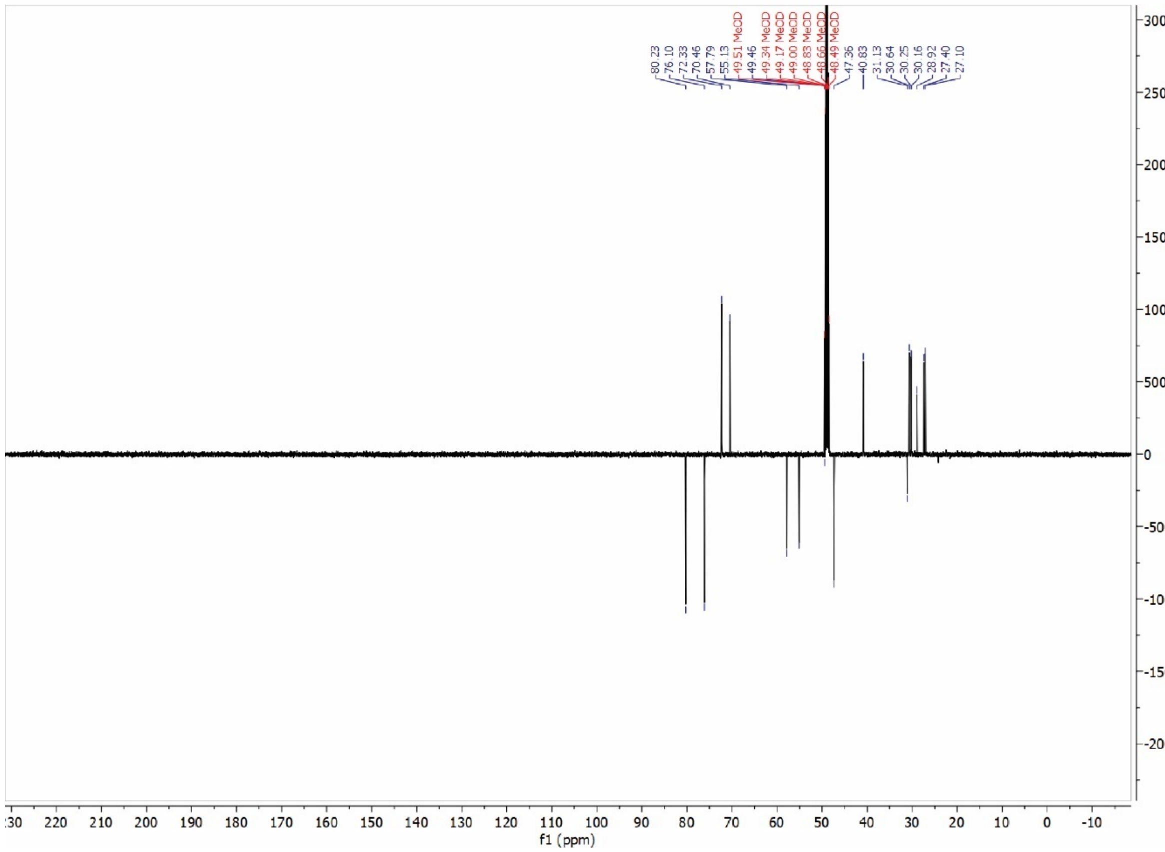

[1]H NMR and [13]C NMR of compound **4** in MeOD:

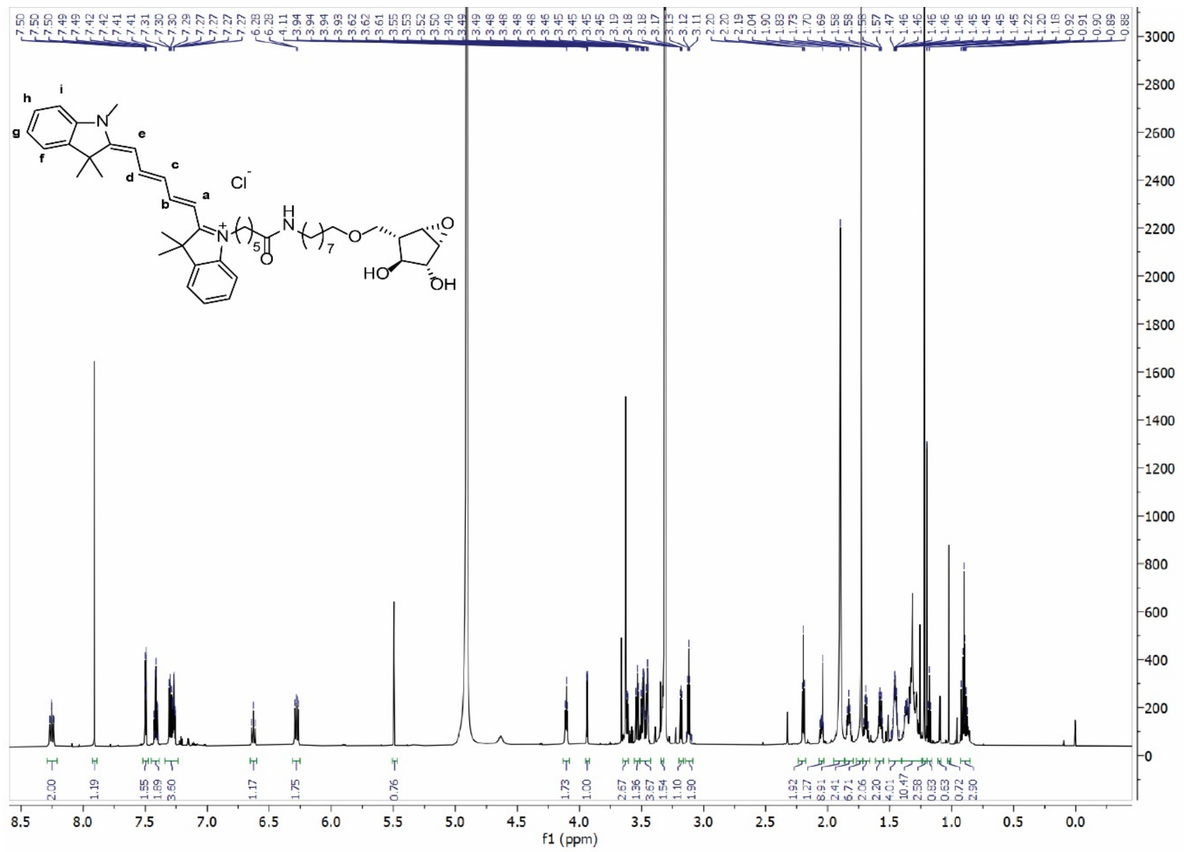


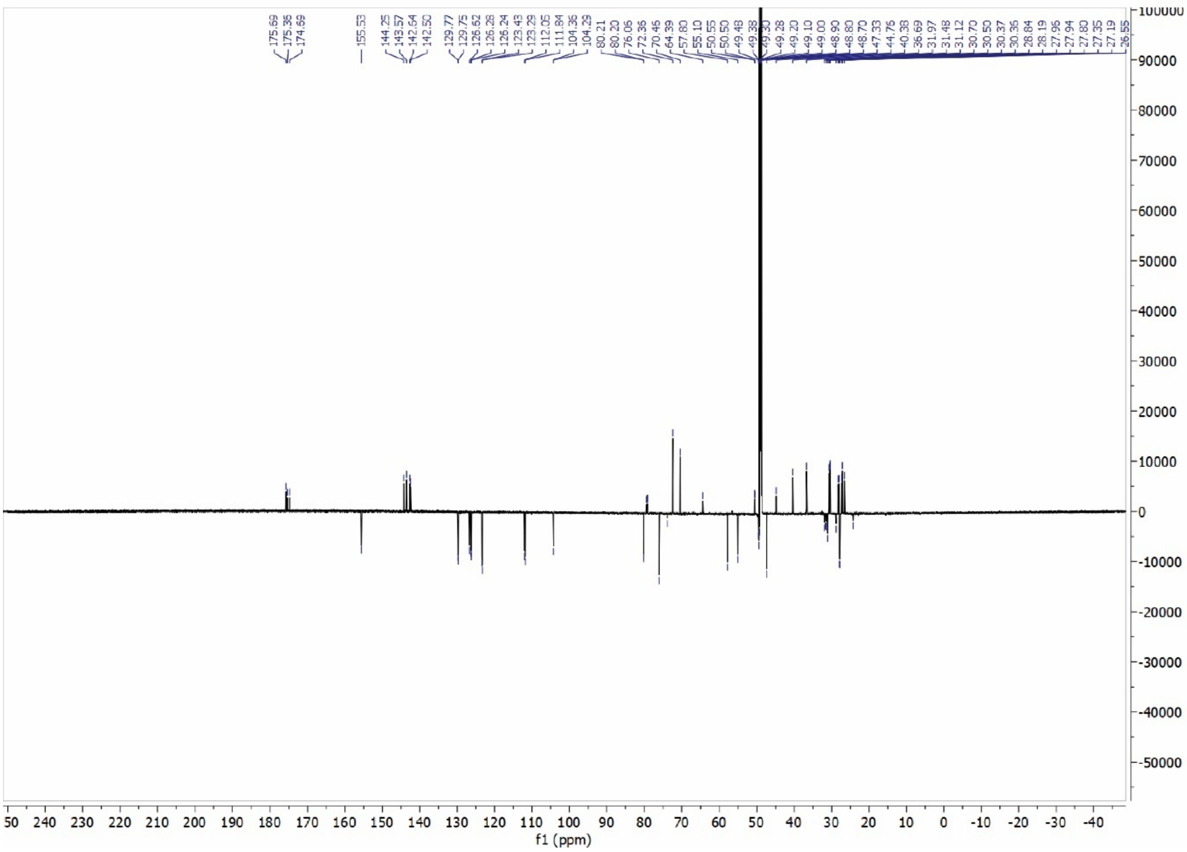

$^{1}H$ NMR and $^{13}C$ NMR of compound **5** in MeOD:

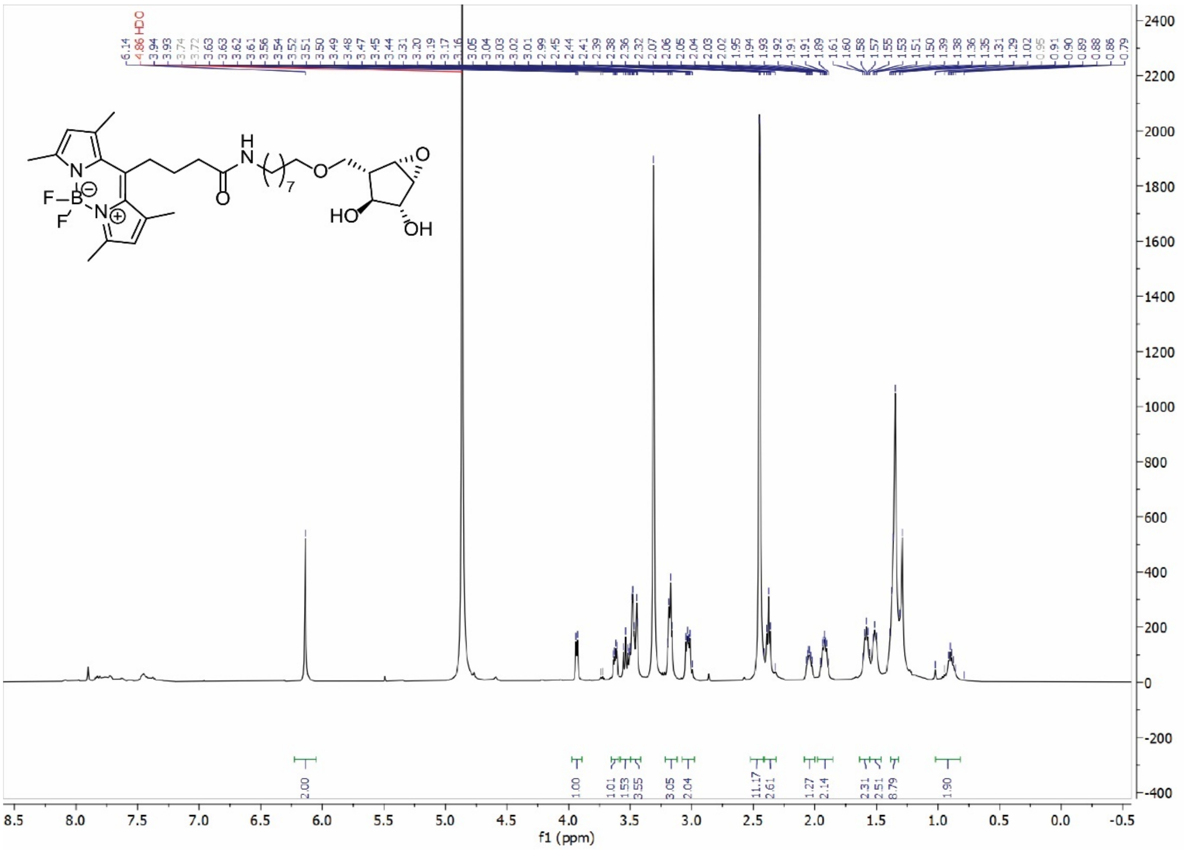

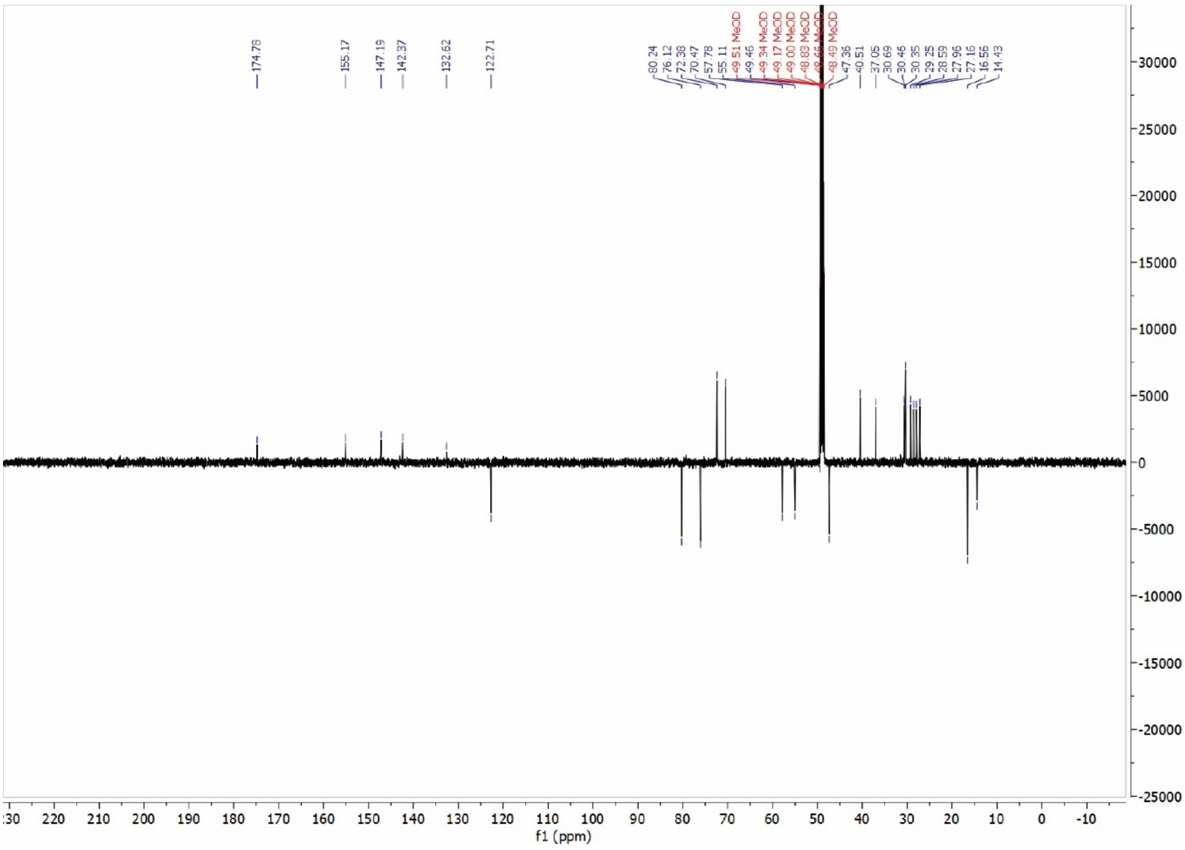

[1]H NMR and [13]C NMR of compound **6** in MeOD:

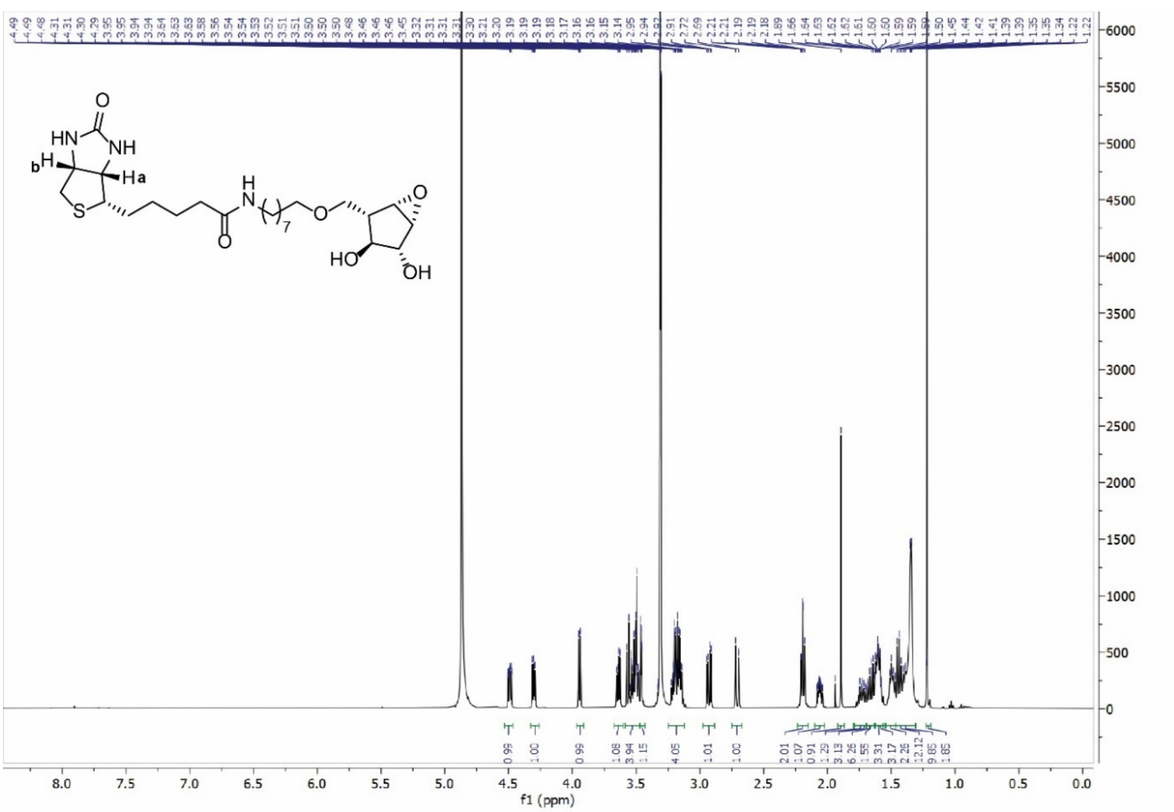

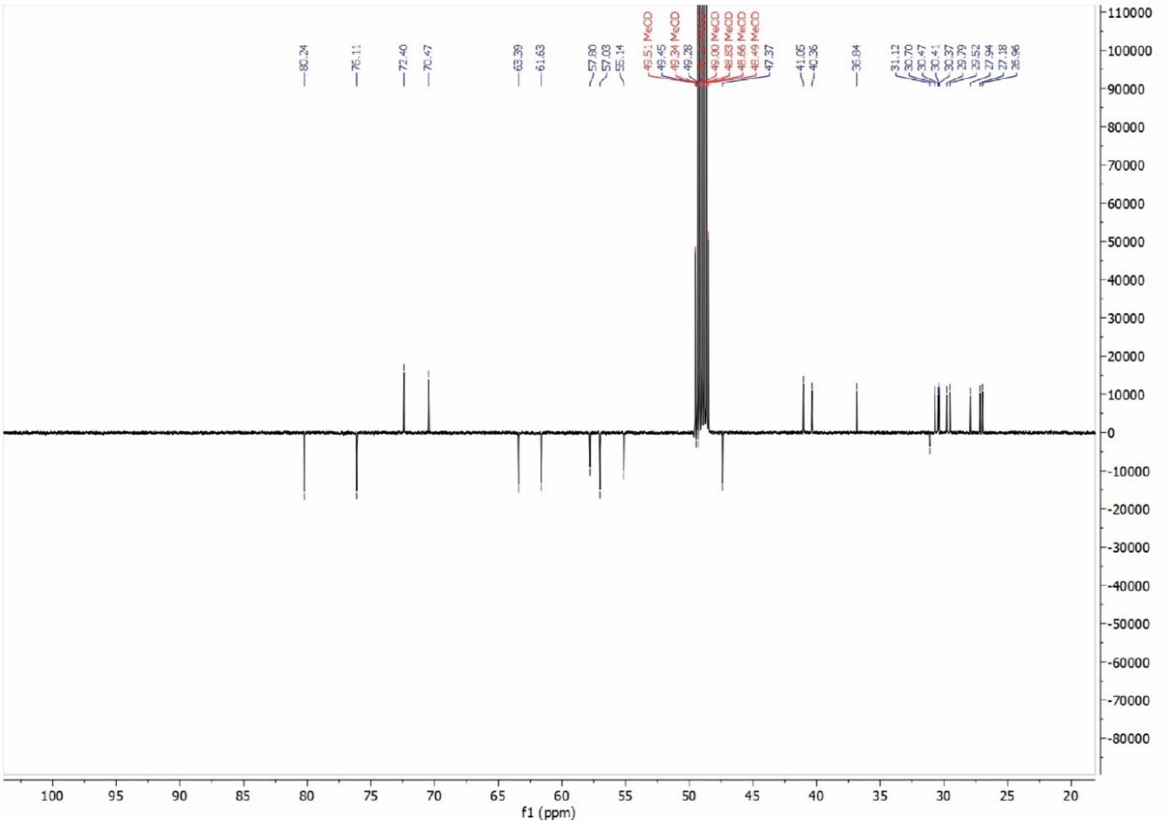

# 4. References


[1] N. G. S. McGregor, J. Coines, V. Borlandelli, S. Amaki, M. Artola, A. Nin‐Hill, D. Linzel, C. Yamada, T. Arakawa, A. Ishiwata, Y. Ito, G. A. van der Marel, J. D. C. Codée, S. Fushinobu, H. S. Overkleeft, C. Rovira, G. J. Davies, Cysteine Nucleophiles in Glycosidase Catalysis: Application of a Covalent β‐l‐Arabinofuranosidase Inhibitor, *Angew. Chem. Int. Ed.* **2021**, *60*, 5754-5758..

[2] S. P. Schröder, J. W. van de Sande, W. W. Kallemeijn, C.-L. Kuo, M. Artola, E. J. van Rooden, J. Jiang, T. J. M. Beenakker, B. I. Florea, W. A. Offen, G. J. Davies, A. J. Minnaard, J. M. F. G. Aerts, J. D. C. Codée, G. A. van der Marel, H. S. Overkleeft, , Synthesis of cyclophellitol, cyclophellitol aziridine, and their tagged derivatives, *Chem. Commun.* **2017**, *53*, 12528–12531.

[3] G. N. Murshudov, P. Skubák, A. A. Lebedev, N. S. Pannu, R. A. Steiner, R. A. Nicholls, M. D. Winn, F. Long, A. A. Vagin, REFMAC5 for the refinement of macromolecular crystal structures, *Acta Crystallogr. Sect. D Biol. Crystallogr.* **2011**, *67*, 355–367.

[4] A. Vagin, A. Teplyakov, Molecular replacement with MOLREP, *Acta Crystallogr. Sect. D Biol. Crystallogr.* **2010**, *66*, 22–25.

[5] R. J. Gildea, J. Beilsten-Edmands, D. Axford, S. Horrell, P. Aller, J. Sandy, J. Sanchez-Weatherby, C. D. Owen, P. Lukacik, C. Strain-Damerell, R. L. Owen, M. A. Walsh, G. Winter, xia2.multiplex: a multi-crystal data-analysis pipeline, *Acta Crystallogr. Sect. D Struct. Biol.* **2022**, *78*, 752-769.

[6] W. Kabsch, XDS, *Acta Cryst.* **2010**, *D66*, 125-132.

[7] R. J. Gildea, J. Beilsten-Edmands, D. Axford, S. Horrell, P. Aller, J. Sandy, J. Sanchez-Weatherby, C. D. Owen, P. Lukacik, C. Strain-Damerell, R. L. Owen, M. A. Walsh, G. Winter, xia2. multiplex: a multi-crystal data-analysis pipeline, *Acta Crystallogr. Sect. D Struct. Biol.* **2022**, *78*, 752-769.

[8] G. Winter, C. M. C. Lobley, S. M. Prince, Decision making in xia2, *Acta Crystallogr. Sect. D Biol. Crystallogr.* **2013**, *69*, 1260–1273.

[9] M. D. Winn, C. C. Ballard, K. D. Cowtan, E. J. Dodson, P. Emsley, P. R. Evans, R. M. Keegan, E. B. Krissinel, A. G. W. Leslie, A. McCoy, S. J. McNicholas, G. N. Murshudov, N. S. Pannu, E. A. Potterton, H. R. Powell, R. J. Read, A. Vagin, K. S. Wilson, Overview of the CCP4 suite and current developments, *Acta Crystallogr. Sect. D* **2011**, *67*, 235-242.

[10] E. Krissinel, A. A. Lebedev, V. Uski, C. B. Ballard, R. M. Keegan, O. Kovalevskiy, R. A. Nicholls, N. S. Pannu, P. Skubák, J. Berrisford, M. Fando, B. Lohkamp, M. Wojdyr, A. J. Simpkin, J. M. H. Thomas, C. Oliver, C. Vonrhein, G. Chojnowski, A. Basle, A. Purkiss, M. N. Isupov, S. McNicholas, E. Lowe, J. Triviño, K. Cowtan, J. Agirre, D. J. Rigden, I. Uson, V. Lamzin, I. Tews, G. Bricogne, A. G. W. Leslie, D. G. Brown, CCP4 Cloud for structure determination and project management in macromolecular crystallography, *Acta Crystallogr. Sect. D Struct. Biol.* **2022**, *78*, 1079-1089.

[11] A. Vagin, A. Teplyakov, Molecular replacement with MOLREP, *Acta Crystallogr. Sect. D Biol. Crystallogr.* **2010**, *66*, 22–25.

[12] G. N. Murshudov, P. Skubák, A. A. Lebedev, N. S. Pannu, R. A. Steiner, R. A. Nicholls, M. D. Winn, F. Long, A. A. Vagin, REFMAC5 for the refinement of macromolecular crystal structures, *Acta Crystallogr. Sect. D Biol. Crystallogr.* **2011**, *67*, 355–367.

[13] P. Emsley, K. Cowtan, Coot: model-building tools for molecular graphics, *Acta Crystallogr Sect. D Biol. Crystallogr.* **2004**, *60*, 2126-2132.

[14] A. A. Lebedev, P. Young, M. N. Isupov, O. V. Moroz, A. A. Vagin, G. N. Murshudov, JLigand: a graphical tool for the CCP4 template-restraint library, *Acta Crystallogr. Sect. D Biol. Crystallogr.* **2012**, *68*, 431–440.

[15] F. Long, R. A. Nicholls, P. Emsley, S. Graǽulis, A. Merkys, A. Vaitkus, G. N. Murshudov, AceDRG: a stereochemical description generator for ligands, *Acta Crystallogr. Sect. D Struct. Biol.* **2017**, *73*, 112-122.

[16] L. Potterton, J. Agirre, C. Ballard, K. Cowtan, E. Dodson, P. R. Evans, H. T. Jenkins, R. Keegan, E. Krissinel, K. Stevenson, A. Lebedev, S. J. McNicholas, R. A. Nicholls, M. Noble, N. S. Pannu, C. Roth, G. Sheldrick, P. Skubak, J. Turkenburg, V. Uski, F. von Delft, D. Waterman, K. Wilson, M. Winn, M. Wojdyr, CCP4i2: the new graphical user interface to the CCP4 program suite, *Acta Crystallogr. Sect. D* **2018**, *74*, 68-84.
